# Asymptotic Analysis of Regular Sequences

**DOI:** 10.1007/s00453-019-00631-3

**Published:** 2019-10-25

**Authors:** Clemens Heuberger, Daniel Krenn

**Affiliations:** Institut für Mathematik, Alpen-Adria-Universität Klagenfurt, Universitätsstraße 65-67, 9020 Klagenfurt am Wörthersee, Austria

**Keywords:** Regular sequence, Mellin–Perron summation, Summatory function, Tauberian theorem, Transducer, Esthetic numbers, Pascal’s rhombus, 05A16, 11A63, 68Q45, 68R05

## Abstract

In this article, *q*-regular sequences in the sense of Allouche and Shallit are analysed asymptotically. It is shown that the summatory function of a regular sequence can asymptotically be decomposed as a finite sum of periodic fluctuations multiplied by a scaling factor. Each of these terms corresponds to an eigenvalue of the sum of matrices of a linear representation of the sequence; only the eigenvalues of absolute value larger than the joint spectral radius of the matrices contribute terms which grow faster than the error term. The paper has a particular focus on the Fourier coefficients of the periodic fluctuations: they are expressed as residues of the corresponding Dirichlet generating function. This makes it possible to compute them in an efficient way. The asymptotic analysis deals with Mellin–Perron summations and uses two arguments to overcome convergence issues, namely Hölder regularity of the fluctuations together with a pseudo-Tauberian argument. Apart from the very general result, three examples are discussed in more detail:

- sequences defined as the sum of outputs written by a transducer when reading a *q*-ary expansion of the input;
- the amount of esthetic numbers in the first *N* natural numbers; and
- the number of odd entries in the rows of Pascal’s rhombus.

For these examples, very precise asymptotic formulæ are presented. In the latter two examples, prior to this analysis only rough estimates were known.

## Part I: Introduction

## Synopsis: The Objects of Interest and the Result

In this paper, we study the asymptotic behaviour of the summatory function of a *q*-regular sequence *x*(*n*). At this point, we give a short overview of the notion of *q*-regular sequences^1^ and our main result.

One characterisation of a *q*-regular sequence is as follows: the sequence *x*(*n*) is said to be *q*-regular if there are square matrices $A_0, \ldots , A_{q-1}$ and a vector-valued sequence *v*(*n*) such that

$$\begin{aligned} v(qn+r)=A_r v(n)\qquad \text {for }0\le r<q\text { and }n\ge 0 \end{aligned}$$

and such that *x*(*n*) is the first component of *v*(*n*).

Regular sequences are intimately related to the *q*-ary expansion of their arguments. They have been introduced by Allouche and Shallit [2]; see also [3, Chapter 16]. Many special cases have been investigated in the literature; this is also due to their relation to divide-and-conquer algorithms. Moreover, every *q*-automatic sequence—those sequences are defined by finite automata—is *q*-regular as well. Take also a look at the book [3] for many examples.

Our main result is, roughly speaking, that the summatory function of a *q*-regular sequence *x*(*n*) has the asymptotic form

1.1 $$\begin{aligned} \sum _{n<N}x(n) = \sum _{j=1}^J N^{\log _q \lambda _j} \frac{(\log N)^{k_j}}{k_j!} \Phi _{k_j}(\{ \log _q N \}) + O(N^{\log _q R}) \end{aligned}$$

as $N\rightarrow \infty$ for a suitable positive integer *J*, suitable constants $\lambda _j\in {\mathbb {C}}$, suitable non-negative integers $k_j$, a suitable *R* and 1-periodic continuous functions $\Phi _{k_j}$. The $\lambda _j$ will turn out to be eigenvalues of $C :=A_0+\cdots +A_{q-1}$, the $k_j$ be related to the multiplicities of these eigenvalues and the constant *R* will be a bound for the joint spectral radius of the matrices $A_0, \ldots , A_{q-1}$.

While (1.1) gives the shape of the asymptotic form, gathering as much information as possible on the periodic fluctuations $\Phi _{k_j}$ is required to have a full picture. To this aim, we will give a description of the Fourier coefficients of the $\Phi _{k_j}$ which allows to compute them algorithmically and therefore to describe these periodic fluctuations with high precision. In particular, this allows to detect non-vanishing fluctuations. Code^2^ is provided to compute the Fourier coefficients.

We close this introductory section by noting that the normalized sum $\frac{1}{N} \sum _{n<N}x(n)$ enlightens us about the expectation of a random element of the sequence *x*(*n*) with respect to uniform distribution on the non-negative integers smaller than a certain *N*.

## How to Read This Paper

This is a long (and perhaps sometimes technical) paper and not all readers might find the time to read it from the very beginning to the very end. We therefore outline reading strategies for various interests.

For the reader who wants to *apply our results to a particular problem*: Read Sect. 3.1 on the definition of *q*-regular sequences and Sect. 3.2 containing the main result in a condensed version which should cover most applications. These two sections also have a simple, illustrative and well-known running example. If it turns out that the refined versions of the results are needed, follow the upcoming paragraph below.

For the reader who still wants to *apply our results to a particular problem* but finds the *condensed version insufficient*, turn to the overview of the results (Sect. 4.1) and then continue with Sect. 6 where the notations and results are stated in full generality. Formulating them will need quite a number of definitions provided in Sect. 6.2. In order to cut straight to the results themselves, we will refrain from motivations and comments on these definitions and postpone those comments to Sect. 7.

For the reader who wants to *determine the asympotics of a regular sequence* instead of determining the asymptotics of the summatory function of the regular sequence, advice is given in Sect. 3.3.

For the reader who wants to read more about *showcase applications* of our method yielding *new asymptotic results*, additionally to Sect. 3 read Sect. 5 where an overview of the examples in this paper is given and then Part II where these examples are discussed in detail. For many more examples to which the methods can be applied, read the original papers [2, 4] and the book by Allouche and Shallit [3] which contain many examples of *q*-regular sequences.

For the reader who wants to *compute the Fourier coefficients* for a particular application, use the provided code. Read Part IV for more details, in particular, see Sect. 19 for some comments on how to decide whether fluctuations are constant or even vanish.

Moreover, for the reader who is interested in the background on the *algorithmic aspects* and details of the implementation of the actual computation, we also refer to Part IV; this part will also be useful for the reader who wants to review the code written for SageMath.

For the reader who is interested in the *history of the problem*, we refer to Sect. 4.4.

For the reader who wants to see a *heuristic argument why everything works out*, there is Sect. 4.2 where it is shown that once one does not care about convergence issues, the Mellin–Perron summation formula of order zero explains the result.

For the reader who wants to understand the *idea of the proof*, there is Sect. 4.3 with a high level overview of the proof how the above mentioned convergence issues with the Mellin–Perron summation formula can be overcome by a pseudo-Tauberian argument.

For the reader who wants to *overcome convergence problems with the Mellin–Perron summation formula* in other contexts involving periodic fluctuations, we note that the pseudo-Tauberian argument (Proposition 14.1) is completely independent of our application to *q*-regular sequences; the only prerequisite is the knowledge on the existence of the fluctuation and sufficient knowledge on analyticity and growth of the Dirichlet generating function. As a consequence, Theorem E has been formulated as an independent result and provisions have been made for several applications of the pseudo-Tauberian argument.

Finally, for the reader who wants to *fully understand the proof*: We have no other advice than reading the whole introduction, the whole Sect. 6 on results and the whole Part III on the proofs starting with a very short Sect. 11 where a few notations used throughout the proofs are fixed.

## User-Friendly Main Result and a First Example Application

### *q*-Regular Sequences

We start by giving a definition of *q*-regular sequences; see Allouche and Shallit [2]. Let $q\ge 2$ be a fixed integer and *x* be a sequence on ${\mathbb {Z}}_{\ge 0}$.^3^ Then *x* is said to be $({\mathbb {C}}, q)$-*regular* (briefly: *q*-*regular* or simply *regular*) if the ${\mathbb {C}}$-vector space generated by its *q*-*kernel*has finite dimension. In other words, *x* is *q*-regular if there are an integer $D$ and sequences $x_1, \ldots , x_D$ such that for every $j\ge 0$ and $0\le r<q^j$ there exist complex numbers $c_1, \ldots , c_D$ with

$$\begin{aligned} x(q^j n+r) = c_1 x_1(n) + \cdots + c_Dx_D(n)\qquad {\text {for all }n\ge 0.} \end{aligned}$$

By Allouche and Shallit [2, Theorem 2.2], the sequence *x* is *q*-regular if and only if there exists a vector-valued sequence *v* whose first component coincides with *x* and there exist square matrices $A_0, \ldots , A_{q-1}\in {\mathbb {C}}^{d\times d}$ such that

3.1 $$\begin{aligned} v(qn+r) = A_r v(n)\qquad \text {for }0\le r<q\text { and }n\ge 0. \end{aligned}$$

This is called a *q*-*linear representation* of the sequence *x*.

The best-known example for a 2-regular function is the binary sum-of-digits function.

#### Example 3.1

For $n\ge 0$, let $x(n)=s(n)$ be the binary sum-of-digits of *n*. We clearly have

3.2 $$\begin{aligned} \begin{aligned} x(2n)&=x(n),\\ x(2n+1)&=x(n)+1 \end{aligned} \end{aligned}$$

for $n\ge 0$. Indeed, we have

$$\begin{aligned} x(2^j n+ r) = x(n) + x(r)\cdot 1 \end{aligned}$$

for integers $j\ge 0$, $0\le r <2^j$ and $n\ge 0$; i.e., the complex vector space generated by the 2-kernel is generated by *x* and the constant sequence $n \mapsto 1$.

Alternatively, we set $v=(x, n \mapsto 1)^\top$ and have

$$\begin{aligned} v(2n)&= \begin{pmatrix} x(n)\\ 1 \end{pmatrix}= \begin{pmatrix} 1&{}\quad 0\\ 0&{}\quad 1 \end{pmatrix}v(n),\\ v(2n+1)&= \begin{pmatrix} x(n)+1\\ 1 \end{pmatrix}= \begin{pmatrix} 1 &{}\quad 1\\ 0 &{}\quad 1 \end{pmatrix}v(n) \end{aligned}$$

for $n\ge 0$. Thus (3.1) holds with

$$\begin{aligned} A_0 = \begin{pmatrix} 1&{}\quad 0\\ 0&{}\quad 1 \end{pmatrix},\qquad A_1 = \begin{pmatrix} 1&{}\quad 1\\ 0&{}\quad 1 \end{pmatrix}. \end{aligned}$$

At this point, we note that a linear representation (3.1) immediately leads to an explicit expression for *x*(*n*) by induction.

#### Remark 3.2

Let $r_{\ell -1}\ldots r_0$ be the *q*-ary digit expansion^4^ of *n*. Then

$$\begin{aligned} x(n) = e_1 A_{r_0}\cdots A_{r_{\ell -1}}v(0) \end{aligned}$$

where $$e_1=\begin{pmatrix}1&\quad 0&\quad \dotsc&\quad 0\end{pmatrix}$$.

### Condensed Main Result

We are interested in the asymptotic behaviour of the summatory function $X(N)=\sum _{0\le n<N}x(n)$.

At this point, we give a simplified version of our results. We choose any vector norm $||{\cdot } ||$ on ${\mathbb {C}}^d$ and its induced matrix norm. We set $C:=\sum _{0 \le r < q} A_r$. We choose $R>0$ such that  holds for all $\ell \ge 0$ and $r_1, \ldots , r_{\ell } \in \{ 0,\ldots ,q-1 \}$. In other words, *R* is an upper bound for the joint spectral radius of $A_0, \ldots , A_{q-1}$. The spectrum of *C*, i.e., the set of eigenvalues of *C*, is denoted by $\sigma (C)$. For $\lambda \in {\mathbb {C}}$, let $m(\lambda )$ denote the size of the largest Jordan block of *C* associated with $\lambda$; in particular, $m(\lambda )=0$ if $\lambda \notin \sigma (C)$. Finally, we consider the scalar-valued Dirichlet series ${\mathcal {X}}$ and the vector-valued Dirichlet series ${\mathcal {V}}$ defined by^5^

$$\begin{aligned} {\mathcal {X}}(s) = \sum _{n\ge 1} n^{-s}x(n) \qquad \text {and}\qquad {\mathcal {V}}(s) = \sum _{n\ge 1} n^{-s}v(n) \end{aligned}$$

where *v*(*n*) is the vector-valued sequence defined in (3.1). Of course, ${\mathcal {X}}(s)$ is the first component of ${\mathcal {V}}(s)$. The principal value of the complex logarithm is denoted by $\log$. The fractional part of a real number *z* is denoted by $\{ z \}:=z-\lfloor z \rfloor$.

#### Theorem A

(User-friendly all-in-one theorem) With the notations above, we have3.3for suitable 1-periodic continuous functions $\Phi _{\lambda k}$. If there are no eigenvalues $\lambda \in \sigma (C)$ with $|\lambda |\le R$, the *O*-term can be omitted.

For $|\lambda |>R$ and $0\le k<m(\lambda )$, the function $\Phi _{\lambda k}$ is Hölder continuous with any exponent smaller than $\log _q(|\lambda |/R)$.

The Dirichlet series ${\mathcal {V}}(s)$ converges absolutely and uniformly on compact subsets of the half plane $\mathfrak {R}s>\log _q R +1$ and can be continued to a meromorphic function on the half plane $\mathfrak {R}s>\log _q R$. It satisfies the functional equation

3.4 $$\begin{aligned} \bigl (I-q^{-s}C\bigr ){\mathcal {V}}(s)= \sum _{1 \le n< q} n^{-s}v(n) + q^{-s}\sum _{0 \le r < q} A_r \sum _{k\ge 1}\left( {\begin{array}{c}-s\\ k\end{array}}\right) \Bigl (\frac{r}{q}\Bigr )^k {\mathcal {V}}(s+k)\qquad \end{aligned}$$

for $\mathfrak {R}s>\log _q R$. The right-hand side of (3.4) converges absolutely and uniformly on compact subsets of $\mathfrak {R}s>\log _q R$. In particular, ${\mathcal {V}}(s)$ can only have poles where $q^s\in \sigma (C)$.

For $\lambda \in \sigma (C)$ with $|\lambda |>R$, the Fourier series

$$\begin{aligned} \Phi _{\lambda k}(u) = \sum _{\ell \in {\mathbb {Z}}}\varphi _{\lambda k\ell }\exp (2\ell \pi i u) \end{aligned}$$

converges pointwise for $u\in {\mathbb {R}}$ where the Fourier coefficients $\varphi _{\lambda k\ell }$ are defined by the singular expansion^6^

3.5 $$\begin{aligned} \frac{x(0)+{\mathcal {X}}(s)}{s} \asymp \sum _{\begin{array}{c} \lambda \in \sigma (C)\\ |\lambda |>R \end{array}}\sum _{\ell \in {\mathbb {Z}}}\sum _{0\le k<m(\lambda )} \frac{\varphi _{\lambda k\ell }}{\bigl (s-\log _q \lambda -\frac{2\ell \pi i}{\log q}\bigr )^{k+1}} \end{aligned}$$

for $$\mathfrak {R}s>\log _q R$$.

This theorem is proved in Sect. 15. We note:

- We write $\Phi _{\lambda k}(\{ \log _q N \})$ to optically emphasise the 1-periodicity; technically, we have $\Phi _{\lambda k}(\{ \log _q N \})=\Phi _{\lambda k}(\log _q N)$.
- The arguments in the proof could be used to meromophically continue the Dirichlet series to the complex plane, but we do not need this result for our purposes. See [1] for the corresponding argument for automatic sequences.
- Sometimes, it will be convenient to write (3.5) in the equivalent explicit formulation 3.6 In particular, this can be used to algorithmically compute the $\varphi _{\lambda k \ell }$.
- Computing the Fourier coefficients $\varphi _{\lambda k \ell }$ via the explicit formulation (3.6) by reliable numerical arithmetic (see Part IV for details) enables us to detect the non-vanishing of a fluctuation; see also the example below and in Sect. 8 (on sequences defined by transducers) for examples where the fluctuation of the leading term is in fact constant. There, additional arguments are required to actually prove this fact; see Sect. 19 for more details.

We come back to the binary sum of digits.

#### Example 3.3

(Continuation of Example 3.1) We have $$C=A_0+A_1=\bigl ( {\begin{matrix} 2&{}1\\ 0&{}2 \end{matrix}}\bigr ) $$. As $A_0$ is the identity matrix, any product $A_{r_1}\cdots A_{r_\ell }$ has the shape $$A_1^k=\bigl ( {\begin{matrix} 1&{}k\\ 0&{}1 \end{matrix}}\bigr ) $$ where *k* is the number of factors $A_1$ in the product. This implies that *R* with  may be chosen to be any number greater than 1. As *C* is a Jordan block itself, we simply read off that the only eigenvalue of *C* is $\lambda =2$ with $m(2)=2$.

Thus Theorem A yieldsfor suitable 1-periodic continuous functions $\Phi _{21}$ and $\Phi _{20}$.

In principle, we can now use the functional equation (3.4) to obtain the Dirichlet series ${\mathcal {X}}$. Due to the fact that one component of *v* is the constant sequence where everything is known, it is more efficient to use an ad-hoc calculation for ${\mathcal {X}}$ by splitting the sum according to the parity of the index and using the recurrence relation (3.2) for *x*(*n*). We obtainwhere the Hurwitz zeta function  has been used. We get3.7As the sum of digits is bounded by the length of the expansion, we have . By combining this estimate withwe see that the sum in (3.7) converges absolutely for $\mathfrak {R}s>0$ and is therefore analytic for $\mathfrak {R}s>0$.

Therefore, the right-hand side of (3.7) is a meromorphic function for $\mathfrak {R}s>0$ whose only pole is simple and at $s=1$ which originates from . Thus, ${\mathcal {X}}(s)$ is a meromorphic function for $\mathfrak {R}s>0$ with a double pole at $s=1$ and simple poles at $1+\frac{2\ell \pi i}{\log 2}$ for $\ell \in {\mathbb {Z}}{\setminus }\{ 0 \}$.

This gives us3.8by (3.6) and (3.7).

We conclude thatWe will explain in Part IV how to compute rigorous numerical values for the Fourier coefficients, in our case those of the fluctuation $\Phi _{20}$ which can be deduced from (3.7). In this particular case of the binary sum-of-digits, simpler and even explicit expressions for the Fourier coefficients have been stated and derived by other authors: they can be obtained in our set-up by rewriting the residues of ${\mathcal {X}}(s)$ in terms of shifted residues of $\sum _{n\ge 1}\left( x(n)-x(n-1)\right) n^{-s}$ and by computing the latter explicitly; see [31, Proof of Corollary 2.5]. This yields the well-known result by Delange [9].

It will also turn out that (3.8) being a constant function is an immediate consequence of the fact that $$ \begin{pmatrix} 0&\quad 1 \end{pmatrix} $$ is a left eigenvector of both $A_0$ and $A_1$ associated with the eigenvalue 1; see Theorem B.

### Asymptotics of Regular Sequences

This article is written with a focus on the sequence of partial sums of a regular sequence. In this section, however, we explain how to use all material for the regular sequence itself.

Let *x*(*N*) be a *q*-regular sequence. We may rewrite it as a telescoping sum

3.9 $$\begin{aligned} x(N) = x(0) + \sum _{n<N} \bigl ( x(n+1) - x(n) \bigr ). \end{aligned}$$

By [2, Theorems 2.5 and 2.6], the sequence of differences $x(n+1) - x(n)$ is again *q*-regular. Conversely, it is also well-known that the summatory function of a *q*-regular sequence is itself *q*-regular. (This is an immediate consequence of [2, Theorem 3.1].)

Therefore, we might also start to analyse a regular sequence by considering it to be the summatory function of its sequence of differences as in (3.9). In this way, we can apply all of the machinery developed in this article.

We end this short section with some remarks on why focusing on the sequence of partial sums can be rewarding. When modelling a quantity by a regular sequences, its asymptotic behaviour is often not smooth, but the asymptotic behaviour of its summatory function is. Moreover, we will see throughout this work that from a technical perspective, considering partial sums is appropriate. Therefore, we adopt this point of view of summatory functions of *q*-regular sequences throughout this paper.

## Overview of the Full Results and Proofs

### Overview of the Results

We have already seen the main results collected in a user-friendly simplified version as Theorem A which was written down in a self-contained way in Sect. 3.2.

In Theorem B the assumptions are refined. In particular, this theorem uses the joint spectral radius *R* of the matrices in a linear representation of the sequence (instead of a suitable bound for this quantity in Theorem A). Theorem B states the contribution of each eigenvalue of the sum *C* of matrices of the linear representation—split into the three cases of smaller, equal and larger in absolute value than *R*, respectively. This is formulated in terms of generalised eigenvectors. As a consequence of this precise breakdown of contributions, Theorem C, which collects the different cases into one result, provides a condition on when the error term vanishes.

Theorem D brings up the full formulation of the functional equation of the Dirichlet series associated to our regular sequence. This is accompanied by a meromorphic continuation as well as bounds on the growth of the Dirichlet series along vertical lines (i.e., points with fixed real value). The analytic properties provided by Theorem D will be used to verify the assumptions of Theorem E.

Theorem E is in fact stated and proved very generally: it is not limited to Dirichlet series coming from matrix products and regular sequences, but it works for general Dirichlet series provided that periodicity and continuity properties of the result are known a priori. This theorem handles the Mellin–Perron summation and the theoretical foundations for the computation of the Fourier coefficients of the appearing fluctuations.

We want to point out that Theorem E can be viewed as a “successful” version of the Mellin–Perron summation formula of order zero. In fact, the theorem states sufficient conditions to provide the analytic justification for the zeroth order formula.

Note that there is another result shown in this article, namely a pseudo-Tauberian theorem for summing up periodic functions. This is formulated as Proposition 14.1, and all the details around this topic are collected in Sect. 14.1. This pseudo-Tauberian argument is an essential step in proving Theorem E.

### Heuristic Approach: Mellin–Perron Summation

The purpose of this section is to explain why the formula (3.5) for the Fourier coefficients is expected. The approach here is heuristic and non-rigorous because we do not have the required growth estimates. See also [10].

By the Mellin–Perron summation formula of order 0 (see, for example, [18, Theorem 2.1]), we have

$$\begin{aligned} \sum _{1\le n<N}x(n) + \frac{x(N)}{2} = \frac{1}{2\pi i}\int _{\max \{ \log _q R + 2,1 \} -i\infty }^{\max \{ \log _q R + 2,1 \} +i\infty } {\mathcal {X}}(s)\frac{N^s\,{\mathrm {d}}s}{s}. \end{aligned}$$

By Remark 3.2 and the definition of *R*, we have . Adding the summand *x*(0) to match our definition of *X*(*N*) amounts to adding . Shifting the line of integration to the left—we have *no analytic justification* that this is allowed—and using the location of the poles of ${\mathcal {X}}$ claimed in Theorem A yieldfor some $\varepsilon >0$. Expanding $N^s$ as

$$\begin{aligned} N^s = \sum _{k\ge 0} \frac{(\log N)^k}{k!} N^{\log _q \lambda + \frac{2\ell \pi i}{\log q}} \Bigl (s-\log _q \lambda -\frac{2\ell \pi i}{\log q}\Bigr )^k \end{aligned}$$

and assuming that the remainder integral converges absolutely yieldwhere $m_{\lambda \ell }$ denotes the order of the pole of ${\mathcal {X}}(s)/s$ at $\log _q\lambda + \frac{2\ell \pi i}{\log q}$ and $\varphi _{\lambda k \ell }$ is as in (3.5). (For $\lambda =1$ and $k=0$, the contribution of *x*(0) / *s* in (3.5) is absorbed by the error term  here.)

Summarising, this heuristic approach explains most of the formulæ in Theorem A. Some details (exact error term and order of the poles) are not explained by this approach. A result “repairing” the zeroth order Mellin–Perron formula is known as Landau’s theorem; see [5, Sect. 9]. It is not applicable to our situation due to multiple poles along vertical lines which then yield the periodic fluctuations. Instead, we present Theorem E which provides the required justification (not by estimating the relevant quantities, but by reducing the problem to higher order Mellin–Perron summation). The essential assumption is that the summatory function can be decomposed into fluctuations multiplied by some growth factors such as in (3.3).

### High Level Overview of the Proof

As we want to use Mellin–Perron summation in some form, we derive properties of the Dirichlet series associated to the regular sequence. In particular, we derive a functional equation which allows to compute the Dirichlet series and its residues with arbitrary precision (Theorem D).

We cannot directly use Mellin–Perron summation of order zero for computing the Fourier coefficients of the fluctuations of interest. As demonstrated in Sect. 4.2, however, our theorems coincide with the results which Mellin–Perron summation of order zero would give if the required growth estimates could be provided. Unfortunately, we are unable to prove these required growth estimates. Therefore, we have to circumvent the problem by applying a generalisation of the pseudo-Tauberian argument by Flajolet, Grabner, Kirschenhofer, Prodinger and Tichy [18].

In order to use this argument, we have to know that the asymptotic formula has the shape (3.3). Note that a successful application (not *directly* possible!) of Mellin–Perron summation of order zero would give this directly. Therefore, we first prove (3.3) and the existence of the fluctuations (Theorems B, C). To do so, we decompose the problem into contributions of the eigenspaces of the matrix $C=A_0+\cdots +A_{q-1}$. The regular sequence is then expressed as a matrix product. Next, we construct the fluctuations by elementary means: We replace finite sums occurring in the summatory functions by infinite sums involving digits using the factorisation as a matrix product.

Then the pseudo-Tauberian argument states that the summatory function of the fluctuation is again a fluctuation and there is a relation between the Fourier coefficients of these fluctuations. The Fourier coefficients of the summatory function of the fluctuation, however, can be computed by Mellin–Perron summation of order one, so the Fourier coefficients of the original fluctuation can be recovered; see Theorem E.

### Relation to Previous Work

The asymptotics of the summatory function of specific examples of regular sequences has been studied in [14, 23, 24]. There, various methods have been used to show that the fluctuations exist; then the original pseudo-Tauberian argument by Flajolet, Grabner, Kirschenhofer, Prodinger and Tichy [18] is used to compute the Fourier coefficients of the fluctuations.

The first version of the pseudo-Tauberian argument in Theorem E was provided in [18]: there, no logarithmic factors were allowed, only values $\gamma$ with $\mathfrak {R}\gamma >0$ were allowed and the result contained an error term of *o*(1) whereas we give a more precise error estimate in order to allow repeated application.

Dumas [12, 13] proved the first part of Theorem A using dilation equations. We re-prove it here in a self-contained way because we need more explicit results than obtained by Dumas (e.g., we need explicit expressions for the fluctuations) to explicitly get the precise structure depending on the eigenspaces (Theorem B). Notice that the order of factors in Dumas’ paper is inconsistent between his versions of (3.1) and Remark 3.2.

A functional equation for the Dirichlet series of an automatic sequence has been proved by Allouche, Mendès France and Peyrière [1].

In Sect. 8 we study transducers. The sequences there are defined as the output sum of transducer automata in the sense of [31]. They are a special case of regular sequences and are a generalisation of many previously studied concepts. In that case, much more is known (variance, limiting distribution, higher dimensional input); see [31] for references and results. A more detailed comparison can be found in Sect. 8. Divide and conquer recurrences (see [11, 32]) can also be seen as special cases of regular sequences.

The present article gives a unified approach which covers all cases of regular sequences. As long as the conditions on the joint spectral radius are met, the main asymptotic terms are not absorbed by the error terms. Otherwise, the regular sequence is so irregular that the summatory function is not smooth enough to allow a result of this shape.

## Overview of the Examples

We take a closer look at three particular examples. In this section, we provide an overview of these examples; all details can be found in Part II.

At first gance it seems that these examples are straight-forward applications of the results. However, we have to reformulate the relevant questions in terms of a *q*-regular sequence and will then provide shortcuts for the computation of the Fourier series. We put a special effort on the details which gives additional insights like dependencies on certain residue classes; see Sect. 5.3. Moreover, the study of these examples also encourages us to investigate symmetries in the eigenvalues; see Sect. 5.4 for an overview and Sect. 6.6 for general considerations.

We start with transducer automata. Transducers have been chosen in order to compare the results here with the previously available results [31]. In some sense, the results complement each other: while the results in [31] also contain information on the variance and the limiting distribution, our approach here yields more terms of the asymptotic expansion of the mean, at least in the general case. Also, it is a class of examples.

We then continue with esthetic numbers. These numbers are an example of an automatic sequence, therefore can be treated by a transducer. However, it turns out that the generic results (the results here and in [31]) degenerate: they are too weak to give a meaningful main term. Therefore a different effort is needed for esthetic numbers. No precise asymptotic results were known previously.

The example on Pascal’s Rhombus is a choice of a regular sequence where all components of the vector sequence have some combinatorial meaning. Again, no precise asymptotic results were known previously.

Section 5.6 contains further examples. Note that there are the two additional Sects. 5.3 and 5.4 pointing out phenomena appearing in the analysis of our examples.

### Transducers

The sum ${\mathcal {T}}(n)$ of the output labels of a complete deterministic finite transducer ${\mathcal {T}}$ when reading the *q*-ary expansion of an integer *n* has been investigated in [31]. As this can be seen as a *q*-regular sequence, we reconsider the problem in the light of our general results in this article; see Sect. 8. For the summatory function, the main terms corresponding to the eigenvalue *q* can be extracted by both results; if there are further eigenvalues larger than the joint spectral radius, our Corollary F allows to describe more asymptotic terms which are absorbed by the error term in [31]. Note, however, that our approach here does not give any readily available information on the variance (this could somehow be repaired for specific examples because regular sequences are known to form a ring) nor on the limiting distribution.

### Esthetic Numbers

In this article, we also contribute a precise asymptotic analysis of *q*-esthetic numbers; see De Koninck and Doyon [8]. These are numbers whose *q*-ary digit expansion satisfies the condition that neighboring digits differ by exactly one. The sequence of such numbers turns out to be *q*-automatic, thus are *q*-regular and can also be seen as an output sum of a transducer; see the first author’s joint work with Kropf and Prodinger [31] or Sect. 8. However, the asymptotics obtained by using the main result of [31] is degenerated in the sense that the provided main term and second order term both equal zero; only an error term remains. On the other hand, using a more direct approach via our main theorem brings up the actual main term and the fluctuation in this main term. We also explicitly compute the Fourier coefficients. The full theorem is formulated in Sect. 9. Prior to this precise analysis, the authors of [8] only performed an analysis of esthetic numbers by digit-length (and not by the number itself).

The approach used in the analysis of *q*-esthetic numbers can easily be adapted to numbers defined by other conditions on the word of digits of their *q*-ary expansion.

### Dependence on Residue Classes

The analysis of *q*-esthetic numbers also brings another aspect into the light of day, namely a quite interesting dependence of the behaviour with respect to *q* on different moduli:

- The dimensions in the matrix approach of [8] need to be increased for certain residue classes of *q* modulo 4 in order to get a formulation as a *q*-automatic and *q*-regular sequence, respectively.
- The main result in [8] already depends on the parity of *q* (i.e., on *q* modulo 2). This reflects our Corollary G by having 2-periodic fluctuations (in contrast to 1-periodic fluctuations in the main Theorem A).
- Surprisingly, the error term in the resulting formula of Corollary G depends on the residue class of *q* modulo 3. This can be seen in the spectrum of the matrix $C=\sum _{0 \le r < q} A_r$: there is an appearance of an eigenvalue 1 in certain cases.
- As an interesting side-note: In the spectrum of *C*, the algebraic multiplicity of the eigenvalue 0 changes again only modulo 2.

### Symmetrically Arranged Eigenvalues

Fluctuations with longer periods (like in the second of the four bullet points above) come from a particular configuration in the spectrum of *C*. Whenever eigenvalues are arranged as vertices of a regular polygon, then their influence can be collected; this results in periodic fluctuations with larger period than 1. We elaborate on the influence of such eigenvalues in Sect. 6.6. This is then used in the particular cases of esthetic numbers and in conjunction with the output sum of transducers. More specifically, in the latter example this yields the second order term in Corollary F; see also [31].

### Pascal’s Rhombus

Beside esthetic numbers, we perform an asymptotic analysis of the number of ones in the rows of Pascal’s rhombus. The rhombus is in some sense a variant of Pascal’s triangle—its recurrence is similar to that of Pascal’s triangle. It turns out that the number of ones in the rows of Pascal’s rhombus can be modelled by a 2-regular sequence.

The authors of [21] investigate this number of ones, but only for blocks whose number of rows is a power of 2. In the precise analysis in Sect. 10 we not only obtain the asymptotic formula, we also explicitly compute the Fourier coefficients.

### Further Examples

There are many further examples of specific *q*-regular sequences which await precise asymptotic analysis, for example the Stern–Brocot sequence [39, A002487], the denominators of Farey tree fractions [39, A007306], the number of unbordered factors of length *n* of the Thue–Morse sequence (see [22]).

The Stern–Brocot sequence is a typical example: it is defined by $x(0)=0$, $x(1)=1$ and

5.1 $$\begin{aligned} \begin{aligned} x(2n)&=x(n),\\ x(2n+1)&=x(n)+x(n+1), \end{aligned} \end{aligned}$$

i.e., the right-hand sides are linear combinations of shifted versions of the original sequence.

Note that recurrence relations like (5.1) are not proper linear representations of regular sequences in the sense of (3.1). The good news, however, is that in general, such a sequence is *q*-regular. The following remark formulates this more explicitly.

#### Remark 5.1

Let *x*(*n*) be a sequence such that there are fixed integers $\ell \le 0\le u$ and constants $c_{rk}$ for $0\le r<q$ and $\ell \le k\le u$ such that

$$\begin{aligned} x(qn+r) = \sum _{\ell \le k\le u} c_{rk}x(n+k) \end{aligned}$$

holds for $0\le r<q$ and $n\ge 0$. Then the sequence *x*(*n*) is *q*-regular with *q*-linear representation for $v(n)=\bigl (x(n+\ell '), \ldots , x(n), \ldots , x(n+u')\bigr )^\top$ where

$$\begin{aligned} \ell '=\left\lfloor {\frac{q\ell }{q-1}}\right\rfloor ,\qquad u'=\left\lceil {\frac{qu}{q-1}}\right\rceil . \end{aligned}$$

Note that if $\ell '<0$, then a simple permutation of the components of *v*(*n*) brings *x*(*n*) to its first component (so that the above is indeed a proper linear representation as defined in Sect. 3.1).

By using this remark on (5.1), we set $v(n)=\bigl (x(n), x(n+1), x(n+2)\bigr )^\top$ and obtain the 2-linear representation

$$\begin{aligned} v(2n)= \begin{pmatrix} 1&{}\quad 0&{}\quad 0\\ 1&{}\quad 1&{}\quad 0\\ 0&{}\quad 1&{}\quad 0 \end{pmatrix}v(n),\qquad v(2n+1)= \begin{pmatrix} 1&{}\quad 1&{}\quad 0\\ 0&{}\quad 1&{}\quad 0\\ 0&{}\quad 1&{}\quad 1 \end{pmatrix}v(n) \end{aligned}$$

for $n\ge 0$ for the Stern–Brocot sequence.

## Full Results

In this section, we fully formulate our results. As pointed out in Remark 3.2, regular sequences can essentially be seen as matrix products. Therefore, we will study these matrix products instead of regular sequences. Theorem A can then be proved as a simple corollary of the results for matrix products; see Sect. 15.

### Problem Statement

Let $q\ge 2$, $d\ge 1$ be fixed integers and $A_0, \ldots , A_{q-1}\in {\mathbb {C}}^{d\times d}$. We investigate the sequence *f* of $d\times d$ matrices such that

6.1 $$\begin{aligned} f(qn+r)=A_r f(n) \quad \text { for }0\le r<q, 0\le n\text { with }qn+r\ne 0 \end{aligned}$$

and $$f(0)=I$$.

Let *n* be an integer with *q*-ary expansion $r_{\ell -1}\ldots r_0$. Then it is easily seen that (6.1) implies that

6.2 $$\begin{aligned} f(n)=A_{r_0}\ldots A_{r_{\ell -1}}. \end{aligned}$$

We are interested in the asymptotic behaviour of $F(N):=\sum _{0\le n<N} f(n)$.

### Definitions and Notations

In this section, we give all definitions and notations which are required in order to state the results. For the sake of conciseness, we do not give any motivations for our definitions here; those are deferred to Sect. 7.

The following notations are essential:

- Let $||{\cdot } ||$ denote a fixed norm on ${\mathbb {C}}^d$ and its induced matrix norm on ${\mathbb {C}}^{d\times d}$.
- We set $B_r :=\sum _{0\le r'<r} A_{r'}$ for $0\le r<q$ and $C:=\sum _{0\le r<q} A_r$.
- The joint spectral radius of $A_0, \ldots , A_{q-1}$ is denoted by  If the set of matrices $A_0, \ldots , A_{q-1}$ has the *finiteness property*, i.e., there is an $\ell >0$ such that  then we set $R=\rho$. Otherwise, we choose $R>\rho$ in such a way that there is no eigenvalue $\lambda$ of *C* with $\rho <|\lambda |\le R$.
- The spectrum of *C*, i.e., the set of eigenvalues of *C*, is denoted by $\sigma (C)$.
- For a positive integer $n_0$, let ${\mathcal {F}}_{n_0}$ be the matrix-valued Dirichlet series defined by
  $$\begin{aligned} {\mathcal {F}}_{n_0}(s) :=\sum _{n\ge n_0} n^{-s}f(n) \end{aligned}$$
  for a complex variable *s*.
- Set $$\chi _k:=\frac{2\pi i k}{\log q}$$ for $$k\in {\mathbb {Z}}$$.

In the formulation of Theorems B and C, the following constants are needed additionally:

- Choose a regular matrix *T* such that $T C T^{-1}=: J$ is in Jordan form.
- Let *D* be the diagonal matrix whose *j*th diagonal element is 1 if the *j*th diagonal element of *J* is not equal to 1; otherwise the *j*th diagonal element of *D* is 0.
- Set $$C':=T^{-1}DJT$$.
- Set $$K:=T^{-1}DT(I-C')^{-1}(I-A_0)$$.
- For a $\lambda \in {\mathbb {C}}$, let $m(\lambda )$ be the size of the largest Jordan block associated with $\lambda$. In particular, $m(\lambda )=0$ if $\lambda \not \in \sigma (C)$.
- For $$m\ge 0$$, set
  $$\begin{aligned} \vartheta _m :=\frac{1}{m!}T^{-1}(I-D)T(C-I)^{m-1}(I-A_0); \end{aligned}$$
  here, $\vartheta _0$ remains undefined if $1\in \sigma (C)$.^7^
- Define $$\vartheta :=\vartheta _{m(1)}$$.

All implicit *O*-constants depend on *q*, *d*, the matrices $A_0, \ldots , A_{q-1}$ (and therefore on $\rho$), as well as on *R*.

### Decomposition into Periodic Fluctuations

Instead of considering *F*(*N*), it is certainly enough to consider *wF*(*N*) for all generalised left eigenvectors *w* of *C*, e.g., the rows of *T*. The result for *F*(*N*) then follows by taking appropriate linear combinations.

#### Theorem B

Let *w* be a generalised left eigenvector of rank *m* of *C* corresponding to the eigenvalue $\lambda$.

1. If $$|\lambda |<R$$, then
2. If $$|\lambda |=R$$, then
3. If $|\lambda |>R$, then there are 1-periodic continuous functions $\Phi _k:{\mathbb {R}}\rightarrow {\mathbb {C}}^d$, $0\le k<m$, such that
  $$\begin{aligned} wF(N)=wK + (\log _q N)^mw\vartheta _m + N^{\log _q\lambda } \sum _{0\le k<m}(\log _q N)^k\Phi _k(\{ \log _q N \}) \end{aligned}$$
  for $N\ge q^{m-1}$. The function $\Phi _k$ is Hölder continuous with any exponent smaller than $\log _q|\lambda |/R$.
  If, additionally, the left eigenvector $w(C-\lambda I)^{m-1}$ of *C* happens to be a left eigenvector to each matrix $A_0, \ldots , A_{q-1}$ associated with the eigenvalue 1, then
  $$\begin{aligned} \Phi _{m-1}(u)=\frac{1}{q^{m-1}(m-1)!}w(C-q I)^{m-1} \end{aligned}$$
  is constant.

Here, $wK=0$ for $\lambda =1$ and $w\vartheta _m=0$ for $\lambda \ne 1$.

This theorem is proved in Sect. 12. Note that in general, the three summands in the theorem have different growths: a constant, a logarithmic term and a term whose growth depends essentially on the joint spectral radius and the eigenvalues larger than the joint spectral radius, respectively. The vector *w* is not directly visible in front of the third summand; instead, the vectors of its Jordan chain are part of the function $\Phi _k$.

Expressing the identity matrix as linear combinations of generalised left eigenvalues and summing up the contributions of Theorem B essentially yields the following corollary.

#### Theorem C

With the notations above, we havefor suitable 1-periodic continuous functions $\Phi _{\lambda k}$. If 1 is not an eigenvalue of *C*, then $\vartheta =0$. If there are no eigenvalues $\lambda \in \sigma (C)$ with $|\lambda |\le \rho$, then the *O*-term can be omitted.

For $|\lambda |>R$, the function $\Phi _{\lambda k}$ is Hölder continuous with any exponent smaller than $\log _q(|\lambda |/R)$.

This theorem is proved in Sect. 12.4.

#### Remark 6.1

We want to point out that the condition $|\lambda |>R$ is inherent in the problem: Single summands *f*(*n*) might be as large as $n^{\log _q R}$ and must therefore be absorbed by the error term in any smooth asymptotic formula for the summatory function.

### Dirichlet Series

This section gives the required result on the Dirichlet series ${\mathcal {F}}_{n_0}$. For theoretical purposes, it is enough to study ${\mathcal {F}}:={\mathcal {F}}_1$; for numerical purposes, however, convergence improves for larger values of $n_0$. This is because for large $n_0$ and large $\mathfrak {R}s$, the value of ${\mathcal {F}}_{n_0}(s)$ is roughly $n_0^{-s} f(n_0)$; see also Part IV.

#### Theorem D

Let $n_0$ be a positive integer. Then the Dirichlet series ${\mathcal {F}}_{n_0}(s)$ converges absolutely and uniformly on compact subsets of the half plane $\mathfrak {R}s > \log _q \rho + 1$, thus is analytic there.

We have

6.3 $$\begin{aligned} \bigl (I-q^{-s}C\bigr ){\mathcal {F}}_{n_0}(s) = {\mathcal {G}}_{n_0}(s) \end{aligned}$$

for $$\mathfrak {R}s>\log _q \rho +1$$ with

6.4 $$\begin{aligned} {\mathcal {G}}_{n_0}(s) = \sum _{n_0 \le n< qn_0} n^{-s}f(n) + q^{-s} \sum _{0 \le r < q} A_r \sum _{k\ge 1} \left( {\begin{array}{c}-s\\ k\end{array}}\right) \Bigl (\frac{r}{q}\Bigr )^k {\mathcal {F}}_{n_0}(s+k). \end{aligned}$$

The series in (6.4) converge absolutely and uniformly on compact sets for $\mathfrak {R}s>\log _q \rho$. Thus (6.3) gives a meromorphic continuation of ${\mathcal {F}}_{n_0}(s)$ to the half plane $\mathfrak {R}s>\log _q \rho$ with possible poles at $s=\log _q \lambda + \chi _\ell$ for each $\lambda \in \sigma (C)$ with $|\lambda |>\rho$ and $\ell \in {\mathbb {Z}}$ whose pole order is at most $m(\lambda )$.

Let $\delta >0$. For real *z*, we set

$$\begin{aligned} \mu _\delta (z)= \max \{ 1 - (z-\log _q \rho -\delta ), 0 \}, \end{aligned}$$

i.e., the linear function on the interval $[\log _q\rho +\delta , \log _q\rho +\delta +1]$ with $\mu _\delta (\log _q\rho +\delta )=1$ and $\mu _\delta (\log _q\rho +\delta +1)=0$. Then6.5holds uniformly for $\log _q \rho +\delta \le \mathfrak {R}s$ and $|q^s-\lambda | \ge \delta$ for all eigenvalues $\lambda \in \sigma (C)$. Here, the implicit *O*-constant also depends on $\delta$.

Note that by the introductory remark on ${\mathcal {F}}_{n_0}(s)$, the infinite sum over *k* in (6.4) can be well approximated by a finite sum. Detailed error bounds are discussed in Part IV. Therefore the theorem allows to transfer the information on ${\mathcal {F}}_{n_0}(s)$ for large $\mathfrak {R}s$ where convergence is unproblematical to values of *s* where the convergence of the Dirichlet series ${\mathcal {F}}_{n_0}$ itself is bad.

#### Remark 6.2

By the identity theorem for analytic functions, the meromorphic continuation of ${\mathcal {F}}_{n_0}$ is unique on the domain given in the theorem. Therefore, the bound (6.5) does not depend on the particular expression for the meromorphic continuation given in (6.3) and (6.4).

Theorem D is proved in Sect. 13. In the proof we translate the linear representation of *f* into a system of equations involving ${\mathcal {F}}_{n_0}(s)$ and shifted versions like $\sum _{n\ge n_0}f(n)(n+\beta )^{-s}$. We will have to bound the difference between the shifted and unshifted versions of the Dirichlet series. These bounds are provided by the following lemma. It will turn out to be useful to have it as a result listed in this section and not buried in the proofs sections.

#### Lemma 6.3

Let ${\mathcal {D}}(s) = \sum _{n \ge n_0} d(n)/n^s$ be a Dirichlet series with coefficients  for all $R'>\rho$. Let $\beta \in {\mathbb {C}}$ with $|\beta |<n_0$ and $\delta >0$. SetThenwhere the series converges absolutely and uniformly on compact sets for $\mathfrak {R}s>\log _q \rho$, thus  is analytic there. Moreover, with $\mu _\delta$ as in Theorem D,as $|\mathfrak {I}s |\rightarrow \infty$ holds uniformly for $\log _q \rho + \delta \le \mathfrak {R}s\le \log _q \rho +\delta +1$.

### Fourier Coefficients

As discussed in Sect. 4.2, we would like to apply the zeroth order Mellin–Perron summation formula but need analytic justification. In the following theorem we prove that whenever it is known that the result is a periodic fluctuation, the use of zeroth order Mellin–Perron summation can be justified. In contrast to the remaining parts of the paper, this theorem does *not* assume that *f*(*n*) is a matrix product.

#### Theorem E

Let *f* be a sequence on ${\mathbb {Z}}_{>0}$, let $\gamma _0\in {\mathbb {R}}{\setminus } {\mathbb {Z}}_{\le 0}$ and $\gamma \in {\mathbb {C}}$ with $\mathfrak {R}\gamma > \gamma _0$, $\delta >0$, $q>1$ be real numbers with $\delta \le \pi /(\log q)$ and $\delta < \mathfrak {R}\gamma -\gamma _0$, and let *m* be a positive integer. Moreover, let $\Phi _j$ be Hölder continuous (with exponent $\alpha$ with $\mathfrak {R}\gamma -\gamma _0<\alpha \le 1$) 1-periodic functions for $0\le j<m$ such that6.6for integers $N\rightarrow \infty$.

For the Dirichlet series ${\mathcal {F}}(s):=\sum _{n\ge 1}n^{-s}f(n)$ assume that

- there is some real number $\sigma _{\mathrm {abs}}\ge \mathfrak {R}\gamma$ such that ${\mathcal {F}}(s)$ converges absolutely for $\mathfrak {R}s>\sigma _{\mathrm {abs}}$;
- the function ${\mathcal {F}}(s)/s$ can be continued to a meromorphic function for $\mathfrak {R}s > \gamma _0-\delta$ such that poles can only occur at $\gamma +\chi _\ell$ for $\ell \in {\mathbb {Z}}$ and such that these poles have order at most *m* and a possible pole at 0; the local expansions are written as
  6.7 $$\begin{aligned} \frac{{\mathcal {F}}(s)}{s}=\frac{1}{(s-\gamma -\chi _\ell )^m}\sum _{j\ge 0}\varphi _{j\ell }(s-\gamma -\chi _\ell )^j \end{aligned}$$
  with suitable constants $\varphi _{j\ell }$ for *j*, $\ell \in {\mathbb {Z}}$;
- there is some real number $\eta >0$ such that for $\gamma _0 \le \mathfrak {R}s \le \sigma _{\mathrm {abs}}$ and $|s-\gamma -\chi _\ell |\ge \delta$ for all $\ell \in {\mathbb {Z}}$, we have 6.8 for $|\mathfrak {I}s |\rightarrow \infty$.

All implicit *O*-constants may depend on *f*, *q*, *m*, $\gamma$, $\gamma _0$, $\alpha$, $\delta$, $\sigma _{\mathrm {abs}}$ and $\eta$.

Then

6.9 $$\begin{aligned} \Phi _j(u) = \sum _{\ell \in {\mathbb {Z}}}\varphi _{j\ell }\exp (2\ell \pi i u) \end{aligned}$$

for $$u\in {\mathbb {R}}$$, $$\ell \in {\mathbb {Z}}$$ and $$0\le j<m$$.

If $\gamma _0<0$ and $\gamma \notin \frac{2\pi i}{\log q}{\mathbb {Z}}$, then ${\mathcal {F}}(0)=0$.

This theorem is proved in Sect. 14. The theorem is more general than necessary for *q*-regular sequences because Theorem D shows that we could use some $0<\eta <1$. However, it might be applicable in other cases, so we prefer to state it in this more general form.

### Fluctuations of Symmetrically Arranged Eigenvalues

In our main results, the occurring fluctuations are always 1-periodic functions. However, if eigenvalues of the sum of matrices of the linear representation are arranged in a symmetric way, then we can combine summands and get fluctuations with longer periods. This is in particular true if all vertices of a regular polygon (with center 0) are eigenvalues.

#### Proposition 6.4

Let $\lambda \in {\mathbb {C}}$, and let $k\ge 0$ and $p>0$ be integers. Denote by $U_p$ the set of *p*th roots of unity. Suppose for each $\zeta \in U_p$ we have a continuous 1-periodic function

$$\begin{aligned} \Phi _{(\zeta \lambda )}(u) = \sum _{\ell \in {\mathbb {Z}}}\varphi _{(\zeta \lambda )\ell }\exp (2\ell \pi i u) \end{aligned}$$

whose Fourier coefficients arefor a suitable function ${\mathcal {D}}$.

Then

6.10 $$\begin{aligned} \sum _{\zeta \in U_p} N^{\log _q (\zeta \lambda )} (\log _q N)^k \Phi _{(\zeta \lambda )}(\{ \log _q N \}) = N^{\log _q \lambda } (\log _q N)^k \Phi (p\{ \log _{q^p} N \})\nonumber \\ \end{aligned}$$

with a continuous *p*-periodic functionwhose Fourier coefficients are

Note that we again write $\Phi (p\{ \log _{q^p} N \})$ to optically emphasise the *p*-periodicity. Moreover, the factor $(\log _q N)^k$ in (6.10) could be cancelled, however it is there to optically highlight the similarities to the main results (e.g.  Theorem A). The proof of Proposition 6.4 can be found in Sect. 16.

The above proposition will be used for proving Corollary F which deals with transducer automata; there, the second order term exhibits a fluctuation with possible period larger than 1. We will also use the proposition for the analysis of esthetic numbers in Sect. 9.

#### Remark 6.5

We can view Proposition 6.4 from a different perspective: A *q*-regular sequence is $q^p$-regular as well (by [2, Theorem 2.9]). Then, all eigenvalues $\zeta \lambda$ of the original sequence become eigenvalues $\lambda ^p$ whose algebraic multiplicity is the sum of the individual multiplicities but the sizes of the corresponding Jordan blocks do not change. Moreover, the joint spectral radius is also taken to the *p*th power. We apply, for example, Theorem A in our $q^p$-world and get again 1-period fluctuations. Note that for actually computing the Fourier coefficients, the approach presented in the proposition seems to be more suitable.

## Remarks on the Definitions

In this section, we give some motivation for and comments on the definitions listed in Sect. 6.2.

### *q*-Regular Sequences Versus Matrix Products

We note one significant difference between the study of *q*-regular sequences as in (3.1) and the study of matrix products (6.2). The recurrence (3.1) is supposed to hold for $qn+r=0$, too; i.e. $v(0)=A_0v(0)$. This implies that *v*(0) is either the zero vector (which is not interesting at all) or that *v*(0) is a right eigenvector of $A_0$ associated with the eigenvalue 1.

We do not want to impose this condition in the study of the matrix product (6.2). Therefore, we exclude the case $qn+r=0$ in (6.1). This comes at the price of the terms *K*, $\vartheta _m$, $\vartheta$ in Theorem B which vanish if multiplied by a right eigenvector to the eigenvalue 1 of $A_0$ from the right. This is the reason why Theorem A has simpler expressions than those encountered in Theorem B.

### Joint Spectral Radius

LetThen the submultiplicativity of the norm and Fekete’s subadditivity lemma [15] imply that $\lim _{\ell \rightarrow \infty }\rho _\ell =\inf _{\ell >0}\rho _{\ell }=\rho$; cf. [37]. In view of equivalence of norms, this shows that the joint spectral radius does not depend on the chosen norm. For our purposes, the important point is that the choice of *R* ensures that there is an $\ell _0>0$ such that $\rho _{\ell _0}\le R$, i.e., $||A_{r_1}\ldots A_{r_{\ell _0}} ||\le R^{\ell _0}$ for all $r_j\in \{ 0,\ldots , q-1 \}$. For any $\ell >0$, we use long division to write $\ell =s\ell _0+r$, and by submultiplicativity of the norm, we get $||A_{r_1}\ldots A_{r_\ell } ||\le R^{s\ell _0} \rho _{r}^r$ and thus7.1for all $r_j\in \{ 0,\ldots ,q-1 \}$ and $\ell \rightarrow \infty$. We will only use (7.1) and no further properties of the joint spectral radius. Note that (6.2) and (7.1) imply thatfor $n\rightarrow \infty$.

As mentioned, we say that the set of matrices $A_0, \ldots , A_{q-1}$, has the *finiteness property* if there is an $\ell >0$ with $\rho _\ell =\rho$; see [34, 35].

### Constants for Theorem B

In contrast to usual conventions, we write matrix representations of endomorphisms as multiplications $x\mapsto xM$ where *x* is a (row) vector in ${\mathbb {C}}^d$ and *M* is a matrix. Note that we usually denote this endomorphism by the corresponding calligraphic letter, for example, the endomorphism represented by the matrix *M* is denoted by ${\mathcal {M}}$.

Consider the endomorphism ${\mathcal {C}}$ which maps a row vector $x\in {\mathbb {C}}^d$ to *xC* and its generalised eigenspaces $W_\lambda$ for $\lambda \in {\mathbb {C}}$. (These are the generalised left eigenspaces of *C*. If $\lambda \notin \sigma (C)$, then $W_\lambda =\{ 0 \}$.) Then it is well-known that ${\mathcal {C}}|_{W_\lambda }$ is an endomorphism of $W_\lambda$ and that ${\mathbb {C}}^d=\bigoplus _{\lambda \in \sigma (C)}W_\lambda$. Let ${\mathcal {T}}$ be the basis formed by the rows of *T*. Then the matrix representation of ${\mathcal {C}}$ with respect to ${\mathcal {T}}$ is *J*.

Let now ${\mathcal {D}}$ be the endomorphism of ${\mathbb {C}}^d$ which acts as identity on $W_\lambda$ for $\lambda \ne 1$ and as zero on $W_1$. Its matrix representation with respect to the basis ${\mathcal {T}}$ is *D*; its matrix representation with respect to the standard basis is $T^{-1}DT$.

Finally, let ${\mathcal {C}}'$ be the endomorphism ${\mathcal {C}}'={\mathcal {C}}\circ {\mathcal {D}}$. As ${\mathcal {C}}$ and ${\mathcal {D}}$ decompose along ${\mathbb {C}}^d=\bigoplus _{\lambda \in \sigma (C)}W_\lambda$ and ${\mathcal {D}}$ commutes with every other endomorphism on $W_\lambda$ for all $\lambda$, we clearly also have ${\mathcal {C}}'={\mathcal {D}}\circ {\mathcal {C}}$. Thus the matrix representation of ${\mathcal {C}}'$ with respect to ${\mathcal {T}}$ is $DJ=JD$; its matrix representation with respect to the standard basis is $T^{-1}DJT=C'$.

Now consider a generalised left eigenvector *w* of *C*. If it is associated to the eigenvalue 1, then $w T^{-1}DT={\mathcal {D}}(w)=0$, $wK=0$ and $wC'={\mathcal {C}}'(w)=0$. Otherwise, that is, if *w* is associated to an eigenvalue not equal to 1, we have $wT^{-1}DT={\mathcal {D}}(w)=w$, $wC'={\mathcal {C}}'(w)={\mathcal {C}}(w)=wC$, $w{C'}^j={{\mathcal {C}}'}^j(w)={\mathcal {C}}^j(w)=wC^j$ for $j\ge 0$ and $w\vartheta _m=0$. Also note that 1 is not an eigenvalue of $C'$, thus $I-C'$ is indeed regular. If 1 is not an eigenvalue of *C*, then everything is simpler: *D* is the identity matrix, $C'=C$, $K=(I-C)^{-1}(I-A_0)$ and $\vartheta =0$.

## Part II: Examples

In this part we investigate three examples in-depth. For an overview, we refer to Sect. 5 where some of the appearing phenomena are discussed as well. Further examples are also mentioned there.

## Sequences Defined by Transducer Automata

We discuss the asymptotic analysis related to transducers; see also Sect. 5.1 for an overview.

### Transducer and Automata

Let us start with two paragraphs recalling some notions around transducer automata. A *transducer automaton* has a finite set of *states* together with *transitions* (directed edges) between these states. Each transition has an *input label* and an *output label* out of the *input alphabet* and the *output alphabet*, respectively. A transducer is said to be *deterministic* and *complete* if for every state and every letter of the input alphabet, there is exactly one transition starting in this state with this input label.

A deterministic and complete transducer processes a word (over the input alphabet) in the following way:

- It starts at its unique initial state.
- Then the transducer reads the word letter by letter and for each letter
  - takes the transition with matching input label,
  - the output label is written, and
  - we proceed to the next state (according to the end of the transition).
- Each state has a *final output label* that is written when we *halt* in this final state; we call a transducer with this property a *subsequential transducer*.

We refer to [6, Chapter 1] for a more detailed introduction to transducers and automata.

Now we are ready to start with the set-up for our example.

### Sums of Output Labels

Let $q\ge 2$ be a positive integer. We consider a complete deterministic subsequential transducer ${\mathcal {T}}$ with input alphabet $\{ 0, \ldots , q-1 \}$ and output alphabet ${\mathbb {C}}$; see [31]. For a non-negative integer *n*, let ${\mathcal {T}}(n)$ be the sum of the output labels (including the final output label) encountered when the transducer reads the *q*-ary expansion of *n*. Therefore, letters of the input alphabet will from now on be called digits.

This concept has been thoroughly studied in [31]: there, ${\mathcal {T}}(n)$ is considered as a random variable defined on the probability space $\{ 0, \ldots , N-1 \}$ equipped with uniform distribution. The expectation in this model corresponds (up to a factor of *N*) to our summatory function $\sum _{0\le n<N}{\mathcal {T}}(n)$. We remark that in [31], the variance and limiting distribution of the random variable ${\mathcal {T}}(n)$ have also been investigated. Most of the results there are also valid for higher dimensional input.

The purpose of this section is to show that ${\mathcal {T}}(n)$ is a *q*-regular sequence and to see that the corresponding results in [31] also follow from our more general framework here. We note that the binary sum of digits considered in Example 3.1 is the special case of $q=2$ and the transducer consisting of a single state which implements the identity map. For additional special cases of this concept; see [31]. Note that our result here for the summatory function contains (fluctuating) terms for all eigenvalues $\lambda$ of the adjacency matrix of the underlying digraph with $|\lambda |>1$ whereas in [31] only contributions of those eigenvalues $\lambda$ with $|\lambda |=q$ are available, all other contributions are absorbed by the error term there.

### Some Perron–Frobenius Theory

We will need the following consequence of Perron–Frobenius theory. By a *component* of a digraph we always mean a strongly connected component. We call a component *final* if there are no arcs leaving the component. The *period* of a component is the greatest common divisor of its cycle lengths. The *final period* of a digraph is the least common multiple of the periods of its final components.

#### Lemma 8.1

Let *D* be a directed graph where each vertex has outdegree *q*. Let *M* be its adjacency matrix and *p* be its final period. Then *M* has spectral radius *q*, *q* is an eigenvalue of *M* and for all eigenvalues $\lambda$ of *M* of modulus *q*, the algebraic and geometric multiplicities coincide and $\lambda = q\zeta$ for some *p*th root of unity $\zeta$.

This lemma follows from setting $t=0$ in [31, Lemma 2.3]. As [31, Lemma 2.3] proves more than we need here and depends on the notions of that article, we extract the relevant parts of [31] to provide a self-contained (apart from Perron–Frobenius theorem) proof of Lemma 8.1.

#### Proof

As usual, the condensation of *D* is the graph resulting from contracting each component of the original digraph to a single new vertex. By construction, the condensation is acyclic.

We choose a refinement of the partial order of the components given by the successor relation in the condensation to a linear order in such a way that the final components come last. Note that this implies that if there is an arc from one component to another, the former component comes before the latter component in our linear order. We then denote the components by ${\mathcal {C}}_1, \ldots , {\mathcal {C}}_k$, ${\mathcal {C}}_{k+1}, \ldots , {\mathcal {C}}_{k+\ell }$ where the first *k* components are non-final and the last $\ell$ are final. Without loss of generality, we assume that the vertices of the original digraph *D* are labeled such that vertices within a component get successive labels and such that the linear order of the components established above is respected.

Therefore, the adjacency matrix *M* is an upper block triagonal matrix of the shapewhere $M_j$ is the adjacency matrix of the component ${\mathcal {C}}_j$.

Each row of the non-negative square matrix *M* has sum *q* by construction. Thus  and therefore the spectral radius of *M* is bounded from above by *q*. As the all ones vector is obviously a right eigenvector associated with the eigenvalue *q* of *M*, the spectral radius of *M* equals *q*. The same argument applies to $M_{k+1}, \ldots , M_{k+\ell }$.

By construction, the matrices $M_{k+1}, \ldots , M_{k+\ell }$ are irreducible. For $1\le j\le \ell$ all eigenvalues $\lambda$ of $M_{k+j}$ of modulus *q* have algebraic and geometric multiplicities 1 by Perron–Frobenius theory and $\lambda = q \zeta$ for some $p_{k+j}$th root of unity $\zeta$ where $p_{k+j}$ is the period of ${\mathcal {C}}_{k+j}$.

By construction, the vertices of the components ${\mathcal {C}}_j$ for $1\le j\le k$ have out-degree at most *q*. We add loops to these vertices to increase their out-degree to *q*, resulting in ${\widetilde{{\mathcal {C}}}}_j$. The corresponding adjacency matrices are denoted by ${\widetilde{M}}_j$. By the above argument, ${\widetilde{M}}_j$ has spectral radius *q* for $1\le j\le k$. As $M_j\le {\widetilde{M}}_j$ (component-wise) and $M_j\ne {\widetilde{M}}_j$ by construction, the spectral radius of $M_j$ is strictly less than *q* by [20, Theorem 8.8.1].

A left eigenvector $v_j$ of $M_{k+j}$ for $1\le j\le \ell$ can easily be extended to a left eigenvector $(0, \ldots , 0, v_j, 0, \ldots , 0)$ of *M*. This observation shows that the geometric multiplicity of any eigenvalue of *M* of modulus *q* is at least its algebraic multiplicity. This concludes the proof. $\square$

### Analysis of Output Sums of Transducers

We consider the states of ${\mathcal {T}}$ to be numbered by $\{ 1, \ldots , d \}$ for some positive integer $d\ge 1$ such that the initial state is state 1. We set ${\mathcal {T}}_j(n)$ to be the sum of the output labels (including the final output label) encountered when the transducer reads the *q*-ary expansion of *n* when starting in state *j*. By construction, we have ${\mathcal {T}}(n)={\mathcal {T}}_1(n)$ and ${\mathcal {T}}_j(0)$ is the final output label of state *j*. We set $y(n)=\bigl ({\mathcal {T}}_1(n), \ldots , {\mathcal {T}}_d(n)\bigr )$. For $0\le r<q$, we define the $d\times d$-dimensional $\{ 0, 1 \}$-matrix $P_r$ in such a way that there is a one in row *j*, column *k* if and only if there is a transition from state *j* to state *k* with input label *r*. The vector $o_r$ is defined by setting its *j*th coordinate to be the output label of the transition from state *j* with input label *r*.

For $n_0\ge 1$, we set

$$\begin{aligned} {\mathcal {X}}(s)=\sum _{n\ge 1}n^{-s}{\mathcal {T}}(n),\qquad {\mathcal {Y}}_{n_0}(s)=\sum _{n\ge n_0}n^{-s}y(n),\qquad \zeta _{n_0}(s, \alpha )=\sum _{n\ge n_0}(n+\alpha )^{-s}. \end{aligned}$$

The last Dirichlet series is a truncated version of the Hurwitz zeta function.

#### Corollary F

Let ${\mathcal {T}}$ be a transducer as described at the beginning of this section. Let *M* be the adjacency matrix and *p* be the final period of the underlying digraph. For $\lambda \in {\mathbb {C}}$ let $m(\lambda )$ be the size of the largest Jordan block associated with the eigenvalue $\lambda$ of *M*.

Then the sequence $n\mapsto {\mathcal {T}}(n)$ is a *q*-regular sequence and8.1for some continuous *p*-periodic function $\Phi$, some continuous 1-periodic functions $\Phi _{\lambda k}$ for $\lambda \in \sigma (M)$ with $1<|\lambda |<q$ and $0\le k<m(\lambda )$ and some constant $e_{\mathcal {T}}$.

Furthermore,

$$\begin{aligned} \Phi (u)=\sum _{\ell \in {\mathbb {Z}}}\varphi _\ell \exp \Bigl (\frac{2\ell \pi i}{p}u\Bigr ) \end{aligned}$$

withfor $\ell \in {\mathbb {Z}}$. The Fourier series expansion of $\Phi _{\lambda k}$ for $\lambda \in \sigma (M)$ with $1<|\lambda |<q$ is given in Theorem A.

The Dirichlet series ${\mathcal {Y}}_{n_0}$ satisfies the functional equation8.2

Note that the functional equation (8.2) is preferrable over the functional equation given in Theorem D for the generic case of a regular sequence: the generic functional equation suggests a double pole at $s=1+\chi _\ell$ for all $\ell \in {\mathbb {Z}}$ whereas the occurrence of the Hurwitz zeta function in (8.2) shows that there is a double pole $s=1$ but single poles at $s=1+\chi _\ell$ for all $\ell \in {\mathbb {Z}}{\setminus }\{0\}$. Numerically, the same occurrence of the Hurwitz zeta function is also advantageous because it allows to decouple the problem.

### Proof of Corollary F

#### Proof of Corollary F

The proof is split into several steps.

*Recursive Description.* We set $v(n)=\bigl ({\mathcal {T}}_1(n), \ldots , {\mathcal {T}}_d(n), 1\bigr )^\top$. For $1\le j\le d$ and $0\le r<q$, we define *t*(*j*, *r*) and *o*(*j*, *r*) to be the target state and output label of the unique transition from state *j* with input label *r*, respectively. Therefore,

8.3 $$\begin{aligned} {\mathcal {T}}_j(qn+r) = {\mathcal {T}}_{t(j, r)}(n) + o(j, r) \end{aligned}$$

for $$1\le j\le d$$, $$n\ge 0$$, $$0\le r<q$$ with $$qn+r>0$$.

For $0\le r<q$, define $A_r=(a_{rjk})_{1\le j,\, k\le d+1}$ by

$$\begin{aligned} a_{rjk} = \left\{ \begin{array}{l@{\quad }l} {[}{t(j, r) = k}]&{} \quad \text {if }j, k\le d,\\ o(j, r)&{} \quad \text {if }j\le d, k=d+1,\\ {[}{k=d+1}]&{} \quad \text {if }j=d+1. \end{array}\right. \end{aligned}$$

Then (8.3) is equivalent to

$$\begin{aligned} v(qn+r) = A_r v(n) \end{aligned}$$

for $n\ge 0$, $0\le r<q$ with $qn+r>0$. Defining *f*(*n*) as in (6.1) for these $A_r$, we see that $v(n)=f(n)v(0)$.

*q*-*Regular Sequence.* If we insist on a proper formulation as a regular sequence, we rewrite (8.3) to

8.4 $$\begin{aligned} {\mathcal {T}}_j(qn+r)= {\mathcal {T}}_{t(j,r)}(n) + o(j, r) + [ r=0 ][ n=0 ]\bigl ({\mathcal {T}}_j(0)-{\mathcal {T}}_{t(j,0)}(0)-o(j, 0)\bigr ) \end{aligned}$$

for $1\le j\le d$, $n\ge 0$, $0\le r<q$. Setting ${\widetilde{v}}(n)=\bigl ({\mathcal {T}}_1(n), \ldots , {\mathcal {T}}_d(n), 1, [ n=0 ]\bigr )$ and ${\widetilde{A}}_r=({\widetilde{a}}_{rjk})_{1\le j,\, k\le d+2}$ with

$$\begin{aligned} {\widetilde{a}}_{rjk} = \left\{ \begin{array}{l@{\quad }l} {[}{t(j, r) = k}]&{} \text {if }j, k\le d,\\ o(j, r)&{} \text {if }j\le d, k=d+1,\\ {[}{r=0}]\bigl ({\mathcal {T}}_j(0)-{\mathcal {T}}_{t(j,0)}(0)-o(j, 0)\bigr )&{} \text {if }j\le d, k=d+2,\\ {[}{k=d+1}]&{} \text {if }j=d+1,\\ {[}{k=d+2}][{r=0}]&{} \text {if }j=d+2, \end{array}\right. \end{aligned}$$

the system (8.4) is equivalent to

$$\begin{aligned} {\widetilde{v}}(qn+r) = {\widetilde{A}}_r {\widetilde{v}}(n) \end{aligned}$$

for $$n\ge 0$$, $$0\le r<q$$.

*Eigenvalue* 1. By construction, the matrices $A_r$ have the shape

$$\begin{aligned} A_r = \left( \begin{array}{c|c} P_r&{}o_r\\ \hline 0&{}1 \end{array} \right) . \end{aligned}$$

It is clear that $(0, \ldots , 0, 1)$ is a left eigenvector of $A_r$ associated with the eigenvalue 1.

*Joint Spectral Radius.* We claim that $A_0, \ldots , A_{q-1}$ have joint spectral radius 1. Let  denote the maximum norm of complex vectors as well as the induced matrix norm, i.e., the maximum row sum norm. Let $j_1, \ldots , j_\ell \in \{ 0,\ldots , q-1 \}$. It is easily shown by induction on $\ell$ that

$$\begin{aligned} A_{j_1}\cdots A_{j_\ell }=\left( \begin{array}{c|c} P&{}b_P\\ \hline 0&{}1 \end{array} \right) \end{aligned}$$

for some $P\in {\mathbb {C}}^{d\times d}$ and $b_P\in {\mathbb {C}}^d$ with  and . Thus, we obtainAs 1 is an eigenvalue of each matrix $A_r$ for $0\le r<q$, the joint spectral radius equals 1, which proves the claim.

*Eigenvectors and Asymptotics*. We now consider $C=\sum _{0\le r<q}A_r$. It has the shape

$$\begin{aligned} C = \left( \begin{array}{c|c} M&{}b_M\\ \hline 0&{}q \end{array} \right) \end{aligned}$$

where $b_M$ is some complex vector.

Let $w_1, \ldots , w_\ell$ be a linearly independent system of left eigenvectors of *M* associated with the eigenvector *q*. If $w_j b_M=0$ for $1\le j\le \ell$, then $(w_1, 0), \ldots , (w_\ell , 0), (0, 1)$ is a linearly independent system of left eigenvectors of *C* associated with the eigenvalue *q*. In that case and because of Lemma 8.1, algebraic and geometric multiplicities of *q* as an eigenvalue of *C* are both equal to $\ell +1$.

Otherwise, assume without loss of generality that $w_1 b_M=1$. Then

$$\begin{aligned} \bigl (w_2 - (w_2 b_M)w_1, 0\bigr ),\, \ldots ,\, \bigl (w_\ell - (w_\ell b_M)w_1, 0\bigr ),\, \bigl (0, 1\bigr ) \end{aligned}$$

is a linearly independent system of left eigenvectors of *C* associated with the eigenvalue *q*. Additionally, $(w_1, 0)$ is a generalised left eigenvector of rank 2 of *C* associated with the eigenvalue *q* with $(w_1, 0)(C-qI)=(0, 1)$. As noted above, the vector (0, 1) is a left eigenvector to each matrix $A_0, \ldots , A_{q-1}$.

Similarly, it is easily seen that any left eigenvector of *M* associated with some eigenvalue $\lambda \ne q$ can be extended uniquely to a left eigenvector of *C* associated with the same eigenvalue. The same is true for chains of generalised left eigenvectors associated with $\lambda \ne q$.

Therefore, in both of the above cases, Theorem B yieldsfor some constant $e_{\mathcal {T}}$ (which vanishes in the first case) and some 1-periodic continuous functions $\Phi _{(q\zeta )}$ and $\Phi _{\lambda k}$ where $\zeta$ runs through the *p*th roots of unity $U_p$ and $\lambda$ through the eigenvalues of *M* with $1<|\lambda |<q$ and $0\le k<m(\lambda )$.

Proposition 6.4 leads to (8.1).

*Fourier Coefficients.* By Theorem A, we have

$$\begin{aligned} \Phi _{(q\zeta )}(u)=\sum _{\ell \in {\mathbb {Z}}}\varphi _{(q\zeta )\ell }\exp (2\ell \pi i u) \end{aligned}$$

withfor a *p*th root of unity $\zeta \in U_p$ and $\ell \in {\mathbb {Z}}$. Therefore and by noting that ${\mathcal {T}}(0)$ does not contribute to the residue, Proposition 6.4 leads to the Fourier series given in the corollary.

*Functional Equation.* By (8.3), we have

$$\begin{aligned} {\mathcal {Y}}_{n_0}(s)&= \sum _{n_0\le n<qn_0} n^{-s}y(n) + \sum _{n\ge n_0}\sum _{0\le r<q}(qn+r)^{-s}y(qn+r)\\&= \sum _{n_0\le n<qn_0} n^{-s}y(n) + \sum _{n\ge n_0}\sum _{0\le r<q}(qn+r)^{-s}\bigl (P_r y(n) + o_r\bigr )\\&= \sum _{n_0\le n<qn_0} n^{-s}y(n) + q^{-s}\sum _{0\le r<q}P_r \sum _{n\ge n_0}\Bigl (n+\frac{r}{q}\Bigr )^{-s}y(n) \\&\quad + q^{-s}\sum _{0\le r<q}\zeta _{n_0}\bigl (s, \tfrac{r}{q}\bigr )o_r. \end{aligned}$$

Using Lemma 6.3 yields the result. $\square$

## Esthetic Numbers

We discuss the asymptotic analysis of esthetic numbers; see also Sect. 5.2 for an overview.

Let again be $q\ge 2$ a fixed integer. We call a non-negative integer *n* a *q-esthetic number* (or simply an *esthetic number*) if its *q*-ary digit expansion $r_{\ell -1} \ldots r_0$ satisfies $|r_j - r_{j-1} | = 1$ for all $j\in \{ 1,\ldots ,\ell -1 \}$; see De Koninck and Doyon [8].

In [8] the authors count *q*-esthetic numbers with a given length of their *q*-ary digit expansion. They provide an explicit (in form of a sum of *q* summands) as well as an asymptotic formula for these counts. We aim for a more precise analysis and head for an asymptotic description of the amount of *q*-esthetic numbers up the an arbitrary value *N* (in contrast to only powers of *q* in [8]).

**Fig. 1:**
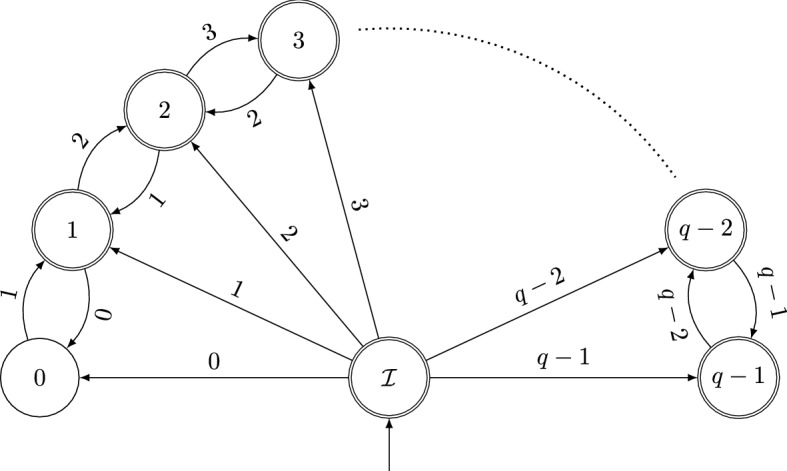
Automaton $\mathcal {A}$ recognizing esthetic numbers

### A *q*-Linear Representation

The language consisting of the *q*-ary digit expansions (seen as words of digits) which are *q*-esthetic is a regular language, because it is recognized by the automaton $\mathcal {A}$ in Fig. 1. Therefore, the indicator sequence of this language, i.e., the *n*th entry is 1 if *n* is *q*-esthetic and 0 otherwise, is a *q*-automatic sequence and therefore also *q*-regular. Let us name this sequence *x*(*n*).

Let $A_0, \ldots , A_{q-1}$ be the transition matrices of the automaton $\mathcal {A}$, i.e., $A_r$ is the adjacency matrix of the directed graph induced by a transition with digit *r*. To make this more explicit, we have the following $(q+1)$-dimensional square matrices: Each row and column corresponds to the states $0, 1,\ldots , q-1$, $\mathcal {I}$. In matrix $A_r$, the only non-zero entries are in column $r\in \{ 0,1,\ldots ,q-1 \}$, namely 1 in the rows $r-1$ and $r+1$ (if available) and in row $\mathcal {I}$ as there are transitions from these states to state *r* in the automaton $\mathcal {A}$.

Let us make this more concrete by considering $q=4$. We obtain the matrices

$$\begin{aligned} A_0&= \begin{pmatrix} 0 &{}\quad 0 &{}\quad 0 &{}\quad 0 &{}\quad 0 \\ 1 &{}\quad 0 &{}\quad 0 &{}\quad 0 &{}\quad 0 \\ 0 &{}\quad 0 &{}\quad 0 &{}\quad 0 &{}\quad 0 \\ 0 &{}\quad 0 &{}\quad 0 &{}\quad 0 &{}\quad 0 \\ 1 &{}\quad 0 &{}\quad 0 &{}\quad 0 &{}\quad 0 \end{pmatrix},\quad A_1 = \begin{pmatrix} 0 &{}\quad 1 &{}\quad 0 &{}\quad 0 &{}\quad 0 \\ 0 &{}\quad 0 &{}\quad 0 &{}\quad 0 &{}\quad 0 \\ 0 &{}\quad 1 &{}\quad 0 &{}\quad 0 &{}\quad 0 \\ 0 &{}\quad 0 &{}\quad 0 &{}\quad 0 &{}\quad 0 \\ 0 &{}\quad 1 &{}\quad 0 &{}\quad 0 &{}\quad 0 \end{pmatrix}, \\ A_2&= \begin{pmatrix} 0 &{}\quad 0 &{}\quad 0 &{}\quad 0 &{}\quad 0 \\ 0 &{}\quad 0 &{}\quad 1 &{}\quad 0 &{}\quad 0 \\ 0 &{}\quad 0 &{}\quad 0 &{}\quad 0 &{}\quad 0 \\ 0 &{}\quad 0 &{}\quad 1 &{}\quad 0 &{}\quad 0 \\ 0 &{}\quad 0 &{}\quad 1 &{}\quad 0 &{}\quad 0 \end{pmatrix},\quad A_3 = \begin{pmatrix} 0 &{}\quad 0 &{}\quad 0 &{}\quad 0 &{}\quad 0 \\ 0 &{}\quad 0 &{}\quad 0 &{}\quad 0 &{}\quad 0 \\ 0 &{}\quad 0 &{}\quad 0 &{}\quad 1 &{}\quad 0 \\ 0 &{}\quad 0 &{}\quad 0 &{}\quad 0 &{}\quad 0 \\ 0 &{}\quad 0 &{}\quad 0 &{}\quad 1 &{}\quad 0 \end{pmatrix}. \end{aligned}$$

We are almost at a *q*-linear representation of our sequence; we still need vectors on both sides of the matrix products. We have

$$\begin{aligned} x(n) = e_{q+1}\, A_{r_0} \cdots A_{r_{\ell -1}} v(0) \end{aligned}$$

for $r_{\ell -1} \dots r_0$ being the *q*-ary expansion of *n* and vectors $$e_{q+1}=\begin{pmatrix}0&\quad \dotsc&\quad 0&\quad 1\end{pmatrix}$$ and $$v(0)=\begin{pmatrix}0&\quad 1&\quad \dotsc&\quad 1\end{pmatrix}^\top $$. As $A_0 v(0)=0\ne v(0)$, this is not a linear representation of a regular sequence. Thus we cannot use Theorem A, but need to use Theorem B. However, the difference is slight: we simply cannot omit the contributions of the constant vector *Kv*(0). However, it will turn out that the joint spectral radius is 1, so the contribution will be absorbed by the error term anyway.

To see that the above holds, we have two different interpretations: the first is that the row vector

$$\begin{aligned} w(n) = e_{q+1}\, A_{r_0} \cdots A_{r_{\ell -1}} \end{aligned}$$

is the unit vector corresponding to the most significant digit of the *q*-ary expansion of *n* or, in view of the automaton $\mathcal {A}$, corresponding to the final state. Note that we read the digit expansion from the least significant digit to the most significant one (although it would be possible the other way round as well). We have $w(0)=e_{q+1}$ which corresponds to the empty word and being in the initial state $\mathcal {I}$ in the automaton. The vector *v*(0) corresponds to the fact that all states of $\mathcal {A}$ except 0 are accepting.

The other interpretation is: the *r*th component of the column vector

$$\begin{aligned} v(n) = A_{r_0} \cdots A_{r_{\ell -1}} v(0) \end{aligned}$$

has the following two meanings:

- In the automaton $\mathcal {A}$, we start in state *r* and then read the digit expansion of *n*. The *r*th component is then the indicator function whether we remain esthetic, i.e., end in an accepting state.
- To a word ending with *r* we append the digit expansion of *n*. The *r*th component is then the indicator function whether the result is an esthetic word.

At first glance, our problem here seems to be a special case of the transducers studied in Sect. 8. However, the automaton $\mathcal {A}$ is not complete. Adding a sink to have a formally complete automaton, however, adds an eigenvalue *q* and thus a much larger dominant asymptotic term, which would then be multiplied by 0. Therefore, the results of [31] do not apply to this case here.

### Full Asymptotics

We now formulate our main result for the amount of esthetic numbers smaller than a given integer *N*. We abbreviate this amount by

$$\begin{aligned} X(N) = \sum _{0 \le n < N} x(n) \end{aligned}$$

and have the following corollary.

#### Corollary G

Fix an integer $q\ge 2$. Then the number *X*(*N*) of *q*-esthetic numbers smaller than *N* is9.1with 2-periodic continuous functions $\Phi _{j}$. Moreover, we can effectively compute the Fourier coefficients of each $\Phi _{j}$ (as explained in Part IV). If *q* is even, then the functions $\Phi _{j}$ are actually 1-periodic. If *q* is odd, then the functions $\Phi _j$ for even *j* vanish.

If $q=2$, then the corollary results in . However, for each length, the only word of digits satisfying the esthetic number condition has alternating digits 0 and 1, starting with 1 at its most significant digit. The corresponding numbers *n* form the so-called Lichtenberg sequence [39, A000975].

**Fig. 2:**
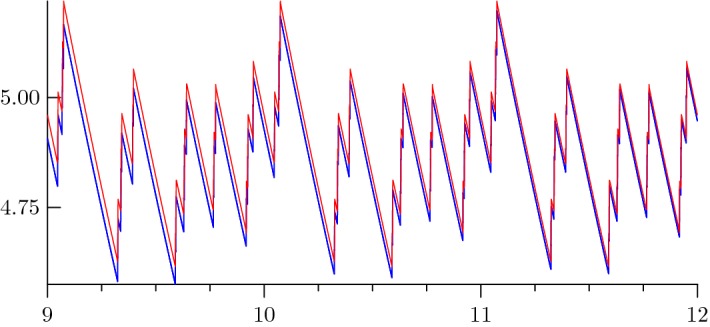
Fluctuation in the main term of the asymptotic expansion of *X*(*N*) for $q=4$. The figure shows  (red) approximated by its trigonometric polynomial of degree 1999 as well as $X(4^u) / N^{u(\log _4(\sqrt{5} + 1) - \frac{1}{2})}$ (blue) (Color figure online)

Back to a general *q*: For the asymptotics, the main quantities influencing the growth are the eigenvalues of the matrix $C = A_0+\cdots +A_{q-1}$. Continuing our example $q=4$ above, this matrix is

$$\begin{aligned} C = A_0 + A_1 + A_2 + A_3 = \begin{pmatrix} 0 &{}\quad 1 &{}\quad 0 &{}\quad 0 &{}\quad 0 \\ 1 &{}\quad 0 &{}\quad 1 &{}\quad 0 &{}\quad 0 \\ 0 &{}\quad 1 &{}\quad 0 &{}\quad 1 &{}\quad 0 \\ 0 &{}\quad 0 &{}\quad 1 &{}\quad 0 &{}\quad 0 \\ 1 &{}\quad 1 &{}\quad 1 &{}\quad 1 &{}\quad 0 \end{pmatrix}, \end{aligned}$$

and its eigenvalues are $\pm \,2\cos (\frac{\pi }{5})=\pm \frac{1}{2}\bigl (\sqrt{5} + 1\bigr ) = \pm 1.618\ldots$, $\pm \, 2\cos (\frac{2\pi }{5})=\pm \frac{1}{2}\bigl (\sqrt{5} - 1\bigr ) = \pm 0.618\ldots$ and 0, all with algebraic and geometric multiplicity 1. Therefore it turns out that the growth of the main term is $N^{\log _4(\sqrt{5} + 1) - \frac{1}{2}}=N^{0.347\ldots }$, see Fig. 2. The first few Fourier coefficients are shown in Table 1.

**Table 1:** Fourier coefficients of $\Phi _1$ for $q=4$ (Corollary G)

$\ell$	$\varphi _{1\ell }$
0	$4.886821584515$
1	$0.036565359077 - 0.012421753685i$
2	$0.0131103199420 - 0.017152133508i$
3	$-\,0.0023895069366 - 0.0506880727105i$
4	$-\,0.017328669452 + 0.025036392542i$
5	$0.011186380630 - 0.0066357472861i$
6	$0.0086354015002 + 0.018593736873i$
7	$-\,0.014899262928 + 0.0297436287202i$
8	$-\,0.003867454968 + 0.0064534688733i$
9	$0.0033747695643 + 0.006159612843i$
10	$-\,0.002149675882 + 0.006474570022i$

All stated digits are correct; see also Part IV

### Eigenvectors

Before proving Corollary G, we collect information on the eigenvalues of *C*.

The matrix $C = A_0+\cdots +A_{q-1}$ has a block decomposition into

$$\begin{aligned} C = \left( \begin{array}{c|c} M &{} \quad \mathbf {0} \\ \hline \mathbf {1} &{}\quad 0 \end{array}\right) \end{aligned}$$

for vectors $\mathbf {0}$ (vector of zeros) and $\mathbf {1}$ (vector of ones) of suitable dimension. Therefore, one eigenvalue of *C* is 0 and the others are the eigenvalues of *M*.

In contrast to [8, Sects. 4 and 5], we use the Chebyshev polynomials^8^$$^{,}$$^9^$$U_n$$ of the second kind defined by

$$\begin{aligned} U_0(X)&= 1,&U_1(X)&=2X,&U_{n+1}(X)=2X\,U_n(X)-U_{n-1}(X) \end{aligned}$$

for $n\ge 1$. It is well-known that

9.2 $$\begin{aligned} U_n(\cos \varphi )=\frac{\sin ((n+1)\varphi )}{\sin (\varphi )} \end{aligned}$$

and, as a consequence, the roots of $U_n$ are given by

$$\begin{aligned} \cos \Bigl (\frac{k\pi }{n+1}\Bigr ), \qquad 1\le k\le n, \end{aligned}$$

for $$n\ge 1$$.

The following lemma is similar to [8, Proposition 3].

#### Lemma 9.1

Let $v\ne 0$ be a vector and $\lambda \in {\mathbb {C}}$.

Then *v* is an eigenvector to the eigenvalue $\lambda$ of *M* if and only if $\lambda = 2\cos (\frac{k\pi }{q+1})$ for some $1\le k\le q$ and

$$\begin{aligned} v=\Bigl (U_j\Bigl (\frac{\lambda }{2}\Bigl )\Bigr )_{0\le j<q} \end{aligned}$$

(up to a scalar factor).

In particular, 0 is an eigenvalue of *M* if and only if *q* is odd.

#### Proof

See the statement and the proof of [8, Proposition 3].$\square$

#### Lemma 9.2

Let $1\le k\le q$, $\lambda =2\cos (k\pi /(q+1))$ and *v* be an eigenvector of *M* to $\lambda$. Then $\langle \mathbf {1}, v\rangle = 0$ holds if and only if *k* is even.

#### Proof

We write $\varphi :=k\pi /(q+1)$. By Lemma 9.1 and (9.2) and a summation similar to the Dirichlet kernel, we have

$$\begin{aligned} \langle \mathbf {1}, v\rangle&=\sum _{0\le j<q}U_j(\cos \varphi )\\&=\frac{1}{\sin \varphi }\sum _{0\le j<q}\sin ((j+1)\varphi )\\&=\frac{1}{\sin \varphi }\mathfrak {I}\sum _{0\le j<q}\exp (i\varphi )^{j+1}\\&=\frac{1}{\sin \varphi } \mathfrak {I}\Bigl (\exp (i\varphi )\frac{1-\exp (iq\varphi )}{1-\exp (i\varphi )}\Bigr )\\&=\frac{1}{\sin \varphi } \mathfrak {I}\Bigl (\exp \Bigl (\frac{i(q+1)\varphi }{2}\Bigr )\frac{\exp \bigl (-\frac{iq\varphi }{2}\bigr )-\exp \bigl (\frac{iq\varphi }{2}\bigr )}{\exp \bigl (-\frac{i\varphi }{2}\bigr )-\exp \bigl (\frac{i\varphi }{2}\bigr )}\Bigr )\\&=\frac{\sin \bigl (\frac{q\varphi }{2}\bigr )}{\sin \varphi \sin \bigl (\frac{\varphi }{2}\bigr )} \mathfrak {I}\exp \Bigl (\frac{i(q+1)\varphi }{2}\Bigr )\\&=\frac{\sin \bigl (\frac{q\varphi }{2}\bigr )\sin \bigl (\frac{(q+1)\varphi }{2}\bigr )}{\sin \varphi \sin \bigl (\frac{\varphi }{2}\bigr )}. \end{aligned}$$

Inserting the value of $\varphi$ leads to

$$\begin{aligned} \langle \mathbf {1}, v\rangle =\frac{\sin \bigl (\frac{qk\pi }{2(q+1)}\bigr )\sin \bigl (\frac{k\pi }{2}\bigr )}{\sin \bigl (\frac{k\pi }{q+1}\bigr )\sin \bigl (\frac{k\pi }{2(q+1)}\bigr )}. \end{aligned}$$

For $1\le k\le q$, it is clear that $0<k\pi /(q+1)<\pi$ and $0<k\pi /(2(q+1))<\pi$, so the denominator of this fraction is non-zero. We also claim that $\sin \bigl (\frac{qk\pi }{2(q+1)}\bigr )\ne 0$: Otherwise, we have $2(q+1)\mid qk$, hence $q+1 \mid qk$, which implies that $q+1\mid k$ because $\gcd (q, q+1)=1$. However, it cannot be that $q+1\mid k$ because $1\le k\le q$.

As a consequence, $\langle \mathbf {1}, v\rangle =0$ if and only if *k* / 2 is an integer. $\square$

#### Lemma 9.3

The characteristic polynomial of *C* is

$$\begin{aligned} X\prod _{1\le k\le q}\Bigl (X-2\cos \Bigl (\frac{k\pi }{q+1}\Bigr )\Bigr ). \end{aligned}$$

In particular, all eigenvalues of *M* apart from 0 are eigenvalues of *C* with algebraic multiplicity 1. If *q* is even, then 0 has algebraic multiplicity 1 as an eigenvalue of *C*; if *q* is odd, then 0 has algebraic multiplicity 2 as an eigenvalue of *C*.

#### Proof

The matrix *C* is a block lower triangular matrix, so the characteristic polynomial is the product of the characteristic polynomials of the matrices *M* and 0.

The statement on the algebraic multiplicities follows from Lemma 9.1. $\square$

We can summarise our findings on the eigenvectors and eigenvalues of *C* as follows.

#### Proposition 9.4

Let $v\in {\mathbb {C}}^{q}$, $w\in {\mathbb {C}}$, not both 0, and let $\lambda \in {\mathbb {C}}$.

Then $$\bigl ({\begin{matrix}v\\ w\end{matrix}}\bigr )\ne 0$$ is an eigenvector of *C* to the eigenvalue $\lambda$ if and only if one of the following conditions hold:

1. $0\ne \lambda = 2\cos \bigl (\frac{k\pi }{q+1}\bigr )$ for some $1\le k\le q$ and $k\ne \frac{q+1}{2}$, *v* is an eigenvector of *M* to $\lambda$, and $w=0$ if *k* is even and $\lambda w=\langle \mathbf {1}, v\rangle \ne 0$ if *k* is odd;
2. $$\lambda =0$$, $$v=0$$, $$w\ne 0$$;
3. $\lambda =0$, $q\equiv 3\pmod 4$, *v* is an eigenvector of *M* and $w=0$.

In particular, the eigenvalue $\lambda =0$ of *C* has

- algebraic and geometric multiplicity 2 if $q\equiv 3\pmod 4$,
- algebraic multiplicity 2 and geometric multiplicity 1 if $q\equiv 1\pmod 4$, and
- algebraic and geometric multiplicity 1 for even *q*.

#### Proof

The vector $$\bigl ({\begin{matrix}v\\ w\end{matrix}}\bigr )$$ is an eigenvector if and only if

$$\begin{aligned} Mv&=\lambda v,\\ \langle \mathbf {1}, v\rangle&=\lambda w. \end{aligned}$$

First assume that $\lambda \ne 0$. Then $v=0$ leads to $w=0$, contradiction. Therefore, *v* is an eigenvector of *M* to the eigenvalue $\lambda$ and $\lambda =2\cos \bigl (\frac{k\pi }{q+1}\bigr )$ for some $1\le k\le q$ by Lemma 9.1. Then $w=0$ if and only if *k* is even by Lemma 9.2.

Now assume that $\lambda =0$ and *q* is even. Then 0 is not an eigenvalue of *M* by Lemma 9.1. Thus $v=0$ and $w\ne 0$.

Now, assume that $\lambda =0$ and $q\equiv 3\pmod 4$. Then $\lambda =2\cos \bigl (\frac{\pi }{2}\bigr )=2\cos \bigl (\frac{\frac{q+1}{2}\pi }{q+1}\bigr )$. By Lemma 9.2, the eigenvector *v* of *M* leads to an eigenvector $$\bigl ({\begin{matrix}v\\ 0\end{matrix}}\bigr )$$ of *C*; and there is an additional eigenvector $$\bigl ({\begin{matrix}0\\ w\end{matrix}}\bigr ) \ne 0$$.

Finally, assume that $\lambda =0$ and $q\equiv 1\pmod 4$. In this case, by Lemma 9.2, it cannot be that $v\ne 0$ is an eigenvector of *M* because this would lead to $0\ne \langle \mathbf {1}, v\rangle =\lambda w=0$, a contradiction. Thus the only eigenvector is $$\bigl ({\begin{matrix}0\\ w\end{matrix}}\bigr ) \ne 0$$. $\square$

### Proof of the Asymptotic Result

#### Proof of Corollary G

We work out the conditions and parameters for using Theorem A.

*Joint Spectral Radius*. As all the square matrices $A_0, \ldots , A_{q-1}$ have a maximum absolute row sum norm equal to 1, the joint spectral radius of these matrices is bounded by 1.

Let $r\in \{ 1,\ldots ,q-1 \}$. Then any product with alternating factors $A_{r-1}$ and $A_r$, i.e., a finite product $A_{r-1}A_rA_{r-1}\cdots$, has absolute row sum norm at least 1 as the word $(r-1)r(r-1)\ldots$ is *q*-esthetic. Therefore the joint spectral radius of $A_{r-1}$ and $A_r$ is at least 1. Consequently, the joint spectral radius of $A_0, \ldots , A_{q-1}$ equals 1.

*Asymptotics.* We apply our Theorem A. We have $\lambda _j=-\lambda _{q+1-j}$, so we combine our approach with Proposition 6.4. Moreover, we have $\lambda _j>1$ iff $\frac{j}{q+1}<\frac{1}{3}$ iff $j\le \lceil \frac{q-2}{3} \rceil$. This results in (9.1).

We now assume that *q* is even. In this case, we still have to show that the functions $\Phi _j$ are actually 1-periodic. We now need to use Theorem B. Let $w_1$, $w_2, \ldots , w_{q-1}$, $w_q$ be the rows of *T* where the order is chosen in such a way that

$$\begin{aligned} J={{\,\mathrm{diag}\,}}\Bigl (2\cos \Bigl (\frac{\pi }{q+1}\Bigr ), \ldots , 2\cos \Bigl (\frac{q\pi }{q+1}\Bigr ), 0\Bigr ). \end{aligned}$$

We write $e_{q+1}=\sum _{k=1}^q c_k w_k$ for suitable $c_k\in {\mathbb {R}}$. Setting $$c:=\begin{pmatrix}c_1&\quad c_2&\quad \cdots&\quad c_q\end{pmatrix}$$, this means that $e_{q+1}=cT$, or equivalently, $c=e_{q+1} T^{-1}$. The columns of $T^{-1}$ are the right eigenvectors of *C* described in Proposition 9.4. Then Proposition 9.4 (1) implies that $c_k=0$ for even *k* with $1\le k\le q$. This means that all fluctuations corresponding to eigenvalues $2\cos (k\pi /(q+1))$ for even *k* with $1\le k\le q$ are multiplied by 0 and do not contribute to the result. As $|\cos (\frac{q+1-k}{q+1}\pi ) |=|\cos (\frac{k}{q+1}\pi ) |$, but $q+1-k$ and *k* have different parities, there is no need to use Proposition 6.4 and all fluctuations are 1-periodic.

The same argument can be used for the case of odd *q*, but in this case, $q+1-k$ and *k* have the same parity. So Proposition 6.4 is used for odd *k*, and fluctuations to both eigenvalues $2\cos (k\pi /(q+1))$ and $2\cos ((q+1-k)\pi /(q+1))$ vanish for even *k*.

*Fourier Coefficients.* We can compute the Fourier coefficients according to Theorem A and Proposition 6.4; see also Part IV. $\square$

## Pascal’s Rhombus

We discuss the asymptotic analysis of odd entries in Pascal’s rhombus; see also Sect. 5.5 for an overview.

We consider Pascal’s rhombus $\mathfrak {R}$ which is, for integers $i\ge 0$ and *j*, the array with entries $r_{i,j}$, where

- $$r_{0,j} = 0$$ all *j*,
- $r_{1,0}=1$ and $r_{1,j}=0$ for all $j\ne 0$,
- and
  $$\begin{aligned} r_{i,j} = r_{i-1,j-1} + r_{i-1,j} + r_{i-1,j+1} + r_{i-2,j} \end{aligned}$$
  for $$i \ge 1$$.

**Fig. 3:**
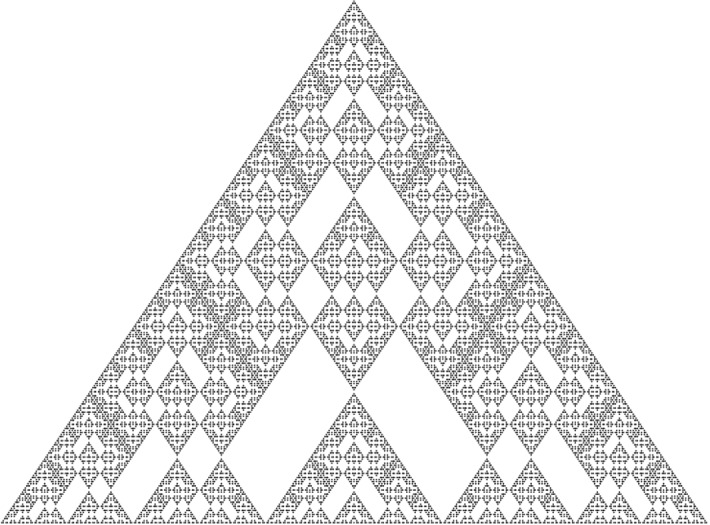
Pascal’s rhombus modulo 2

We are interested in the number of odd entries in the first *N* rows of this rhombus. In [21] the authors investigate this quantity for *N* being a power of 2. We again aim for a more precise analysis and asymptotic description.

So, let $\mathfrak {X}$ be equal to $\mathfrak {R}$ but with entries taken modulo 2; see also Fig. 3. We partition $\mathfrak {X}$ into the four sub-arrays

- $\mathfrak {E}$ consisting only of the rows and columns of $\mathfrak {X}$ with even indices, i.e., the entries $r_{2i, 2j}$,
- $\mathfrak {Y}$ consisting only of the rows with odd indices and columns with even indices, i.e., the entries $r_{2i-1, 2j}$,
- $\mathfrak {Z}$ consisting only of the rows with even indices and columns with odd indices, i.e., the entries $r_{2i, 2j-1}$, and
- $\mathfrak {N}$ consisting only of the rows and columns with odd indices, i.e., the entries $r_{2i-1, 2j-1}$.

Note that $\mathfrak {E} = \mathfrak {X}$ and $\mathfrak {N}=0$; see [21].

### Recurrence Relations and 2-Regular Sequences

Let *X*(*N*), *Y*(*N*) and *Z*(*N*) be the number of ones in the first *N* rows (starting with row index 1) of $\mathfrak {X}$, $\mathfrak {Y}$ and $\mathfrak {Z}$, respectively.

Goldwasser, Klostermeyer, Mays and Trapp [21, (12)–(14)] get the recurrence relations

$$\begin{aligned} X(N)&= X(\lfloor \tfrac{N}{2} \rfloor ) + Y(\lceil \tfrac{N}{2} \rceil ) + Z(\lfloor \tfrac{N}{2} \rfloor ), \\ Y(N)&= X(\lceil \tfrac{N}{2} \rceil ) + X(\lfloor \tfrac{N}{2} \rfloor -1) + Z(\lfloor \tfrac{N}{2} \rfloor ) + Z(\lceil \tfrac{N}{2} \rceil -1), \\ Z(N)&= 2 X(\lfloor \tfrac{N}{2} \rfloor ) + 2 Y(\lceil \tfrac{N}{2} \rceil ) \end{aligned}$$

for $N\ge 2$, and $X(0)=Y(0)=Z(0)=0$, $X(1)=1$, $Y(1)=1$ and $Z(1)=2$ (cf. [21, Figs. 2 and 3]). Distinguishing between even and odd indices gives

$$\begin{aligned} X(2N)&= X(N) + Y(N) + Z(N), \\ X(2N+1)&= X(N) + Y(N+1) + Z(N), \\ Y(2N)&= X(N) + X(N-1) + Z(N) + Z(N-1), \\ Y(2N+1)&= X(N+1) + X(N-1) + 2Z(N), \\ Z(2N)&= 2X(N) + 2Y(N), \\ Z(2N+1)&= 2X(N) + 2Y(N+1) \end{aligned}$$

for all $N\ge 1$. Now we build the backward differences $x(n) = X(n) - X(n-1)$, $y(n) = Y(n) - Y(n-1)$ and $z(n) = Z(n) - Z(n-1)$. These *x*(*n*), *y*(*n*) and *z*(*n*) are the number of ones in the *n*th row of $\mathfrak {X}$, $\mathfrak {Y}$ and $\mathfrak {Z}$, respectively, and clearly

$$\begin{aligned} X(N) = \sum _{1\le n \le N} x(n), \qquad Y(N) = \sum _{1\le n \le N} y(n), \qquad Z(N) = \sum _{1\le n \le N} z(n) \end{aligned}$$

holds. We obtain

10.1a $$\begin{aligned} x(2n)&=x(n)+z(n),&x(2n+1)&=y(n+1), \end{aligned}$$

10.1b $$\begin{aligned} y(2n)&= x(n-1)+z(n),&y(2n+1)&=x(n+1) +z(n), \end{aligned}$$

10.1c $$\begin{aligned} z(2n)&= 2x(n),&z(2n+1)&=2y(n+1) \end{aligned}$$

for $n\ge 1$, and $x(0)=y(0)=z(0)=0$, $x(1)=1$, $y(1)=1$ and $z(1)=2$.

Let us write our coefficients as the vector

10.2 $$\begin{aligned} v(n) = \bigl (x(n), x(n+1), y(n+1), z(n), z(n+1)\bigr )^\top . \end{aligned}$$

It turns out that the components included into *v*(*n*) are sufficient for a self-contained linear representation of *v*(*n*). In particular, it is not necessary to include *y*(*n*). By using the recurrences (10.1), we find that

$$\begin{aligned} v(2n) = A_0 v(n) \qquad \text {and}\qquad v(2n+1) = A_1 v(n) \end{aligned}$$

for all^10^$n\ge 0$ with the matrices

$$\begin{aligned} A_0 = \begin{pmatrix} 1 &{}\quad 0 &{}\quad 0 &{}\quad 1 &{}\quad 0 \\ 0 &{}\quad 0 &{}\quad 1 &{}\quad 0 &{}\quad 0 \\ 0 &{}\quad 1 &{}\quad 0 &{}\quad 1 &{}\quad 0 \\ 2 &{}\quad 0 &{}\quad 0 &{}\quad 0 &{}\quad 0 \\ 0 &{}\quad 0 &{}\quad 2 &{}\quad 0 &{}\quad 0 \end{pmatrix} \quad \text {and}\quad A_1 = \begin{pmatrix} 0 &{}\quad 0 &{}\quad 1 &{}\quad 0 &{}\quad 0 \\ 0 &{}\quad 1 &{}\quad 0 &{}\quad 0 &{}\quad 1 \\ 1 &{}\quad 0 &{}\quad 0 &{}\quad 0 &{}\quad 1 \\ 0 &{}\quad 0 &{}\quad 2 &{}\quad 0 &{}\quad 0 \\ 0 &{}\quad 2 &{}\quad 0 &{}\quad 0 &{}\quad 0 \end{pmatrix}, \end{aligned}$$

and with $v(0) = (0,1,1,0,2)^\top$. Therefore, the sequences *x*(*n*), *y*(*n*) and *z*(*n*) are 2-regular.

### Full Asymptotics

#### Corollary H

We have10.3with $\kappa = \log _2 \bigl (3+\sqrt{17}\,\bigr )-1 = 1.83250638358045\ldots$ and a 1-periodic function $\Phi$ which is Hölder continuous with any exponent smaller than $\kappa -1$.

Moreover, we can effectively compute the Fourier coefficients of $\Phi$ (as explained in Part IV).

**Fig. 4:**
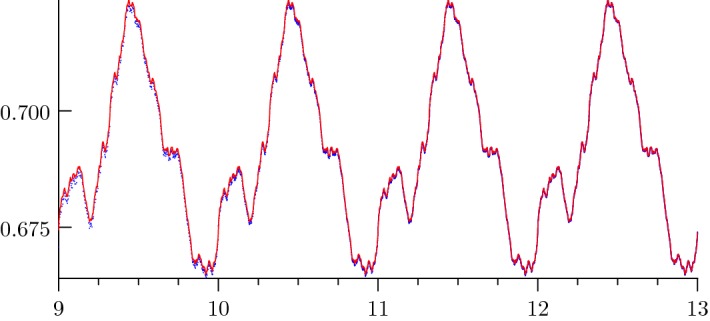
Fluctuation in the main term of the asymptotic expansion of *X*(*N*). The figure shows  (red) approximated by its trigonometric polynomial of degree 1999 as well as $X(2^u) / 2^{u\kappa }$ (blue) (Color figure online)

We get analogous results for the sequences *Y*(*N*) and *Z*(*N*) (each with its own periodic function $\Phi$, but the same exponent $\kappa$). The fluctuation $\Phi$ of *X*(*N*) is visualized in Fig. 4 and its first few Fourier coefficients are shown in Table 2.

**Table 2:** Fourier coefficients of $\Phi$ (Corollary H)

$\ell$	$\varphi _\ell$
0	0.6911615112341912755021246
1	$-0.01079216311240407872950510 - 0.0023421761940286789685827i$
2	$0.00279378637350495172116712 - 0.00066736128659728911347756i$
3	$-0.00020078258323645842522640 - 0.0031973663977645462669373i$
4	$0.00024944678921746747281338 - 0.0005912995467076061497650i$
5	$$ -0.0003886698612765803447578 + 0.00006723866319930148568431i$$
6	$$ -0.0006223575988893574655258 + 0.00043217220614939859781542i$$
7	$$ 0.00023034317364181383130476 - 0.00058663168772856091427688i$$
8	$$ 0.0005339060804798716172593 - 0.0002119380802590974909465i$$
9	$$ 0.0000678898389770175928529 - 0.00038307823285486235280185i$$
10	$$ -0.00019981745997355255061991 - 0.00031394569060142799808175i$$

All stated digits are correct; see also Part IV

### Proof of the Asymptotic Result

At this point, we only prove (10.3) of Corollary H. We deal with the Fourier coefficients in Sect. 10.5. As in the introductory example of the binary sum-of-digits functions (Example 3.1), we could get Fourier coefficients by Theorem A and the 2-linear representation of Sect. 10.1 directly. However, the information in the vector *v*(*n*) [see (10.2)] is redundant with respect to the asymptotic main term as it contains *x*(*n*) and *z*(*n*) as well as $x(n+1)$ and $z(n+1)$; both pairs are asymptotically equal in the sense of (10.3). Therefore, we head for an only 3-dimensional functional system of equations for our Dirichlet series of *x*(*n*), *y*(*n*) and *z*(*n*) (instead of a 5-dimensional system).

#### Proof of (10.3)

We use Theorem A.

*Joint Spectral Radius.* First we compute the joint spectral radius $\rho$ of $A_0$ and $A_1$. Both matrices have a maximum absolute row sum equal to 2, thus $\rho \le 2$, and both matrices have 2 as an eigenvalue. Therefore we obtain $\rho =2$. Moreover, the finiteness property of the linear representation is satisfied by considering only products with exactly one matrix factor $A_0$ or $A_1$.

Thus, we have $R=\rho =2$.

*Eigenvalues.* Next, we compute the spectrum $\sigma (C)$ of $C=A_0+A_1$. The matrix *C* has the eigenvalues $\lambda _1=\bigl (3+\sqrt{17}\,\bigr )/2=3.5615528128088\ldots$, $\lambda _2=2$, $\lambda _3=-2$, $\lambda _4=-1$ and $\lambda _5=\bigl (3-\sqrt{17}\,\bigr )/2=-0.5615528128088\ldots$ (each with multiplicity one). Note that $\lambda _1$ and $\lambda _5$ are the zeros of the polynomial $U^2-3U-2$.

*Asymptotics.* By using Theorem A, we obtain an asymptotic formula for $X(N-1)$. Shifting from $N-1$ to *N* does not change this asymptotic formula, as this shift is absorbed by the error term . $\square$

### Dirichlet Series and Meromorphic Continuation

In the lemma below, we provide the functional equation (10.4) as a system of three equations. This is in contrast to the generic functional equation provided by Theorem D which is a system of five equations.

Let $n_0\ge 2$ be an integer and define

#### Lemma 10.1

Set

$$\begin{aligned} M = I - \begin{pmatrix} 2^{-s} &{}\quad 2^{-s} &{}\quad 2^{-s} \\ 2^{1-s} &{}\quad 0 &{}\quad 2^{1-s} \\ 2^{1-s} &{}\quad 2^{1-s} &{}\quad 0 \\ \end{pmatrix}. \end{aligned}$$

Then10.4wherewith the notion of $\Sigma$ as in Lemma 6.3, provides meromorphic continuations of the Dirichlet series , , and  for $\mathfrak {R}s > \kappa _0=1$ with the only possible poles at $\kappa + \chi _\ell$ for $\ell \in {\mathbb {Z}}$, all of which are simple poles.

#### Proof

We split the proof into several steps.

*Functional Equation.* From (10.1b) we obtain10.5The second row of (10.4) follows. Similarly, (10.1a) and (10.1c) yield the first and third rows of (10.4), respectively.

*Determinant and Zeros.* The determinant of *M* isIt is an entire function.

All zeros of $\Delta$ are simple zeros. In particular, solving  gives $2^s = 3/2 \pm \sqrt{17}/2$ (the two zeros of $U^2-3U-U$) and $2^s = -2$. A solution  implies that $s_0 + 2\pi i \ell /\log 2$ with $\ell \in {\mathbb {Z}}$ satisfies the same equation as well.

Moreover, set $\kappa =\log _2 \bigl (3+\sqrt{17}\,\bigr ) - 1 = 1.8325063835804\ldots$. Then the only zeros with $\mathfrak {R}s > \kappa _0=1$ are at $\kappa + \chi _\ell$ with $\chi _\ell = 2\pi i \ell / \log 2$ for $\ell \in {\mathbb {Z}}$.

It is no surprise that the $\kappa$ of this lemma and the $\kappa$ in the proof of Corollary H which comes from the 2-linear representation of Sect. 10.1 coincide.

*Meromorphic Continuation.* Let ${\mathcal {D}}_{n_0}\in \{ {\mathcal {X}}_{n_0},{\mathcal {Y}}_{n_0},{\mathcal {Z}}_{n_0} \}$. The Dirichlet series  is analytic for $\mathfrak {R}s > 2 = \log _2 \rho + 1$ with $\rho =2$ being the joint spectral radius by Theorem D. We use the functional equation (10.4) which provides the continuation, as we write  in terms of ,  and . By Lemma 6.3, these three functions are analytic for $\mathfrak {R}s > 1$.

The zeros (all are simple zeros) of the denominator  are the only possibilities for the poles of  for $\mathfrak {R}s > 1$. $\square$

### Fourier Coefficients

We are now ready to prove the rest of Corollary H.

#### Proof of Corollary H

We verify that we can apply Theorem E.

The steps of this proof in Sect. 10.2 provided us already with an asymptotic expansion (10.3). Lemma 10.1 gives us the meromorphic function for $\mathfrak {R}s>\kappa _0=1$ which comes from the Dirichlet series . It can only have poles (all simple) at $s=\kappa + \chi _\ell$ for $\ell \in {\mathbb {Z}}$ and satisfies the assumptions in Theorem E by Theorem D and Remark 6.2.

Therefore a computation of the Fourier coefficients via computing residues [see (3.6)] is possible by Theorem E, and this residue may be computed from (10.4) via Cramer’s rule. $\square$

We refer to Part IV for details on the actual computation of the Fourier coefficients.

## Part III: Proofs

Before reading this part on the collected proofs, it is recommended to recall the definitions and notations of Sect. 6.2. Some additional notations which are only used in the proofs are introduced in the following section.

## Additional Notations

We use Iverson’s convention $[ \textit{expr} ]=1$ if $\textit{expr}$ is true and 0 otherwise, which was popularised by Graham, Knuth and Patashnik [26]. We use the notation $z^{\underline{\ell }}:=z(z-1)\cdots (z-\ell +1)$ for falling factorials. We use $$\left( {\begin{array}{c}n\\ k_1,\ldots c, k_r\end{array}}\right) $$ for multinomial coefficients. We sometimes write a binomial coefficient $$\left( {\begin{array}{c}n\\ a\end{array}}\right) $$ as $$\left( {\begin{array}{c}n\\ a, b\end{array}}\right) $$ with $a+b=n$ when we want to emphasise the symmetry and analogy to a multinomial coefficient.

## Decomposition into Periodic Fluctuations: Proof of Theorem B

We first give an overview over the proof.

### Overview of the Proof of Theorem B

The first step will be to express the summatory function *F* in terms of the matrices *C*, $B_r$ and $A_r$. Essentially, this corresponds to the fact that the summatory function of a *q*-regular function is again *q*-regular. This expression of *F* will consist of two terms: the first is a sum over $0\le j<\log _q N$ involving a *j*th power of *C* and matrices $B_r$ and $A_r$ depending on the $\ell -j$ most significant digits of *N*. The second term is again a sum, but does not depend on the digits of *N*; it only encodes the fact that $f(0)=A_0 f(0)$ may not hold. The fact that we are interested in *wF*(*N*) for the generalised left eigenvector *w* corresponding to the eigenvalue $\lambda$ allows to express $wC^j$ in terms of $w\lambda ^j$ (plus some other terms if *w* is not an eigenvector).

The second term can be disposed of by elementary observations using a geometric series. We reverse the order of summation in the first summand and extend it to an infinite sum. The infinite sum is written in terms of periodic fluctuations; the difference between the infinite sum and the finite sum is absorbed by the error term. In order not to have to deal with ambiguities due to non-unique *q*-ary expansions of real numbers, we define the fluctuations on an infinite product space instead of the unit interval. $\square$

### Upper Bound for Eigenvalues of *C*

We start with an upper bound for the eigenvalues of *C* in terms of the joint spectral radius.

#### Lemma 12.1

Let $$\lambda \in \sigma (C)$$. Then $$|\lambda |\le q\rho $$.

#### Proof

For $\ell \rightarrow \infty$, we haveandby (7.1). Taking $\ell$th roots and the limit $\ell \rightarrow \infty$ yields $|\lambda |\le qR$. This last inequality does not depend on our particular (cf.  Sect. 6.2) choice of $R>\rho$, so the inequality is valid for all $R>\rho$, and we get the result. $\square$

### Explicit Expression for the Summatory Function

In this section, we give an explicit formula for $F(N)=\sum _{0\le n<N} f(n)$ in terms of the matrices $A_r$, $B_r$ and *C*.

#### Lemma 12.2

Let *N* be an integer with *q*-ary expansion $r_{\ell -1}\ldots r_0$. Then

$$\begin{aligned} F(N)=\sum _{0\le j<\ell } C^j B_{r_j} A_{r_{j+1}}\cdots A_{r_{\ell -1}} + \sum _{0\le j<\ell } C^j (I-A_0). \end{aligned}$$

#### Proof

We claim that

12.1 $$\begin{aligned} F(qN+r)=C F(N) + B_r f(N) + (I-A_0)[ qN+r > 0 ] \end{aligned}$$

holds for non-negative integers *N* and *r* with $0\le r<q$.

We now prove (12.1): Using (6.1) and $f(0)=I$ yields

$$\begin{aligned} F(qN+r)&= f(0)\, [ qN+r> 0 ] + \sum _{\begin{array}{c} 0<qn+r'<qN+r\\ 0\le n\\ 0\le r'<q \end{array}} f(qn+r')\\&= f(0)\, [ qN+r> 0 ] + \sum _{\begin{array}{c} 0<qn+r'<qN+r\\ 0\le n\\ 0\le r'<q \end{array}} A_{r'}f(n)\\&= \bigl (f(0)-A_{0}f(0)\bigr ) [ qN+r> 0 ] + \sum _{\begin{array}{c} 0\le qn+r'<qN+r\\ 0\le n\\ 0\le r'<q \end{array}} A_{r'}f(n)\\&= (I-A_0) [ qN+r> 0 ] + \sum _{0\le n<N}\sum _{0\le r'<q} A_{r'}f(n) + \sum _{0\le r'<r} A_{r'}f(N)\\&= (I-A_0) [ qN+r > 0 ] + CF(N)+B_{r}f(N). \end{aligned}$$

This concludes the proof of (12.1).

Iteration of (12.1) and using (6.2) yield the assertion of the lemma; cf. [31, Lemma 3.6]. $\square$

### Proof of Theorem B

#### Proof of Theorem B

For readability, this proof is split into several steps.

*Setting.* Before starting the actual proof, we introduce the setting using an infinite product space which will be used to define the fluctuations $\Phi _k$. We also introduce the maps linking the infinite product space to the unit interval.

**Fig. 5:**
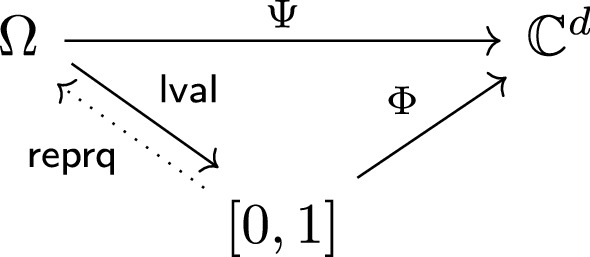
Maps in the proof of Theorem B

We will first introduce functions $\Psi _k$ defined on the infinite product spaceWe equip it with the metric such that two elements ${\mathbf {x}}\ne {\mathbf {x}}'$ with a common prefix of length *j* and $x_j\ne x'_j$ have distance $q^{-j}$. We consider the map ${\mathsf {lval}}:\Omega \rightarrow [0, 1]$ with

$$\begin{aligned} {\mathsf {lval}}({\mathbf {x}}) :=\log _q\sum _{j\ge 0}x_jq^{-j}; \end{aligned}$$

see Fig. 5. By using the assumption that the zeroth component of elements of $\Omega$ is assumed to be non-zero, we easily check that ${\mathsf {lval}}$ is Lipschitz-continuous; i.e.,12.2for ${\mathbf {x}}\ne {\mathbf {x}}'$ with a common prefix of length *j*.

For $y\in [0, 1)$, let ${\mathsf {reprq}}(y)$ be the unique ${\mathbf {x}}\in \Omega$ with ${\mathsf {lval}}({\mathbf {x}})=y$ such that ${\mathbf {x}}$ does not end on infinitely many digits $q-1$, i.e., ${\mathsf {reprq}}(y)$ represents a *q*-ary expansion of $q^y$. This means that ${\mathsf {lval}}\circ {\mathsf {reprq}}$ is the identity on [0, 1).

From the definition of the metric on $\Omega$, recall that a function $\Psi :\Omega \rightarrow {\mathbb {C}}^d$ is continuous if and only if for each $\varepsilon >0$, there is a *j* such that $||\Psi ({\mathbf {x}}')-\Psi ({\mathbf {x}}) ||<\varepsilon$ holds for all ${\mathbf {x}}$ and ${\mathbf {x}}'$ that have a common prefix of length *j*. Further recall from the universal property of quotients that if such a continuous function $\Psi$ satisfies $\Psi ({\mathbf {x}})=\Psi ({\mathbf {x}}')$ whenever ${\mathsf {lval}}({\mathbf {x}})={\mathsf {lval}}({\mathbf {x}}')$, then there is a unique continuous function $\Phi :[0, 1]\rightarrow {\mathbb {C}}^d$ such that $\Phi \circ {\mathsf {lval}}=\Psi$. This will be used in the “Descent”-step of the proof.

*Notation.* We will deal with the two sums in Lemma 12.2 separately. We will first introduce notations corresponding to this split and to the eigenvector structure.

Let *N* have the *q*-ary expansion $r_{\ell -1}\ldots r_0$ and set

$$\begin{aligned} F_1(N) :=\sum _{0\le j<\ell } C^j B_{r_j} A_{r_{j+1}}\ldots A_{r_{\ell -1}}, \qquad F_2(N) :=\sum _{0\le j<\ell } C^j(I-A_0) \end{aligned}$$

so that $F(N)=F_1(N)+F_2(N)$ by Lemma 12.2.

We consider the Jordan chain $w=v_{0}', \ldots , v_{m-1}'$ generated by *w*, i.e., $v_k'=w(C-\lambda I)^k$ for $0\le k<m$ and $v_{m-1}'$ is a left eigenvector of *C*. Thus we have $$wC^j=\sum _{0\le k<m}\left( {\begin{array}{c}j\\ k\end{array}}\right) \lambda ^{j-k}v_k'$$ for all $j\ge 0$. If $\lambda \ne 0$, choose vectors $v_0, \ldots , v_{m-1} \in {\mathbb {C}}^d$ such that

12.3 $$\begin{aligned} wC^j=\lambda ^j\sum _{0\le k<m}j^kv_k \end{aligned}$$

holds for all $j\ge 0$. These vectors are suitable linear combinations of the vectors $v_0', \ldots , v_{m-1}'$. We note that we have

12.4 $$\begin{aligned} v_{m-1}=\frac{1}{\lambda ^{m-1}(m-1)!}v_{m-1}'. \end{aligned}$$

*Second Summand.* We will now rewrite $wF_2(N)$ by evaluating the geometric sum and rewriting it in terms of a fluctuation.

We claim that12.5for suitable continuously differentiable functions $\Phi ^{(2)}_{ k}$ on ${\mathbb {R}}$, $0\le k<m$. If $R=0$, then  shall mean that the error vanishes for almost all *N*.

Consider first the case that $\lambda \ne 1$. Because of $wC^j=w{C'}^j$ and $wT^{-1}DT=w$ (see Sect. 7.3) we have

$$\begin{aligned} wF_2(N)&=\sum _{0\le j<\ell }w{C'}^j \bigl (I-A_0\bigr )\\&=w\bigl (I-{C'}^\ell \bigr )\bigl (I-C'\bigr )^{-1}\bigl (I-A_0\bigr ) = wK - wC^\ell \bigl (I-C'\bigr )^{-1}\bigl (I-A_0\bigr ). \end{aligned}$$

If $\lambda =0$, then $wC^\ell =0$ for almost all $\ell$. We may set $\Phi ^{(2)}_k=0$ for $0\le k<m$ and (12.5) is shown. Otherwise, as we have $\ell -1=\lfloor \log _q N \rfloor =\log _q N - \{ \log _q N \}$ and by (12.3), we can rewrite $wC^\ell$ as

$$\begin{aligned} wC^\ell =\lambda ^{\ell }\sum _{0\le k'<m}\ell ^{k'} v_{k'}=\lambda ^{1+\log _q N-\{ \log _q N \}}\sum _{0\le k'<m}(\log _q N+1-\{ \log _q N \})^{k'} v_{k'}. \end{aligned}$$

Let

$$\begin{aligned} G_2(L, \nu ):=-\lambda ^{1-\nu }\sum _{0\le k'<m}(L+1-\nu )^{k'} v_{k'}(I-C')^{-1}(I-A_0) \end{aligned}$$

for reals *L* and $\nu$, i.e.,

$$\begin{aligned} wF_2(N)=wK + \lambda ^{\log _q N} G_2(\log _q N, \{ \log _q N \}). \end{aligned}$$

By the binomial theorem, we have

$$\begin{aligned} G_2(L, \nu )=-\lambda ^{1-\nu }\sum _{0\le k<m}L^k\sum _{\begin{array}{c} 0\le r \\ k+r<m \end{array}}\left( {\begin{array}{c}k+r\\ k, r\end{array}}\right) (1-\nu )^r v_{k+r}(I-C')^{-1}(I-A_0). \end{aligned}$$

This leads to a representation $G_2(L, \nu )=\sum _{0\le k<m}L^k\Phi ^{(2)}_{ k}(\nu )$ for continuously differentiable functions

$$\begin{aligned} \Phi _k^{(2)}(\nu )=-\lambda ^{1-\nu }\sum _{\begin{array}{c} 0\le r <m-k \end{array}}\left( {\begin{array}{c}k+r\\ k, r\end{array}}\right) (1-\nu )^r v_{k+r}(I-C')^{-1}(I-A_0) \end{aligned}$$

for $0\le k<m$. As the functions $\Phi ^{(2)}_{k}$ are continuously differentiable, they are Lipschitz continuous on compact subsets of ${\mathbb {R}}$. We note that in the case $k=m-1$, the only occurring summand is for $r=0$, which implies that

12.6 $$\begin{aligned} \Phi _{m-1}^{(2)}(\nu ) = -\lambda ^{1-\nu }v_{m-1}(I-C')^{-1}(I-A_0). \end{aligned}$$

Rewriting $\lambda ^{\log _q N}$ as $N^{\log _q \lambda }$ and recalling that $w\vartheta _m=0$ yields (12.5) for $\lambda \ne 1$.

We now turn to the case $\lambda =1$. We use $$wC^j=\sum _{0\le k<m}\left( {\begin{array}{c}j\\ k\end{array}}\right) v_k'$$ for $j\ge 0$ as above. Thus

$$\begin{aligned} wF_2(N)= & {} \sum _{0\le j<\ell }\sum _{0\le k<m}\left( {\begin{array}{c}j\\ k\end{array}}\right) v'_{k}(I-A_0)\\= & {} \sum _{0\le k<m}v'_k(I-A_0)\sum _{0\le j<\ell }\left( {\begin{array}{c}j\\ k\end{array}}\right) \\= & {} \sum _{0\le k<m}v'_k(I-A_0) \left( {\begin{array}{c}\ell \\ k+1\end{array}}\right) , \end{aligned}$$

where the identity [26, (5.10)] (“summation on the upper index”) has been used in the last step.

Thus $wF_2(N)$ is a polynomial in $\ell$ of degree *m*. By writing $\ell =1+\log _qN-\{ \log _q N \}$, we can again rewrite this as a polynomial in $\log _q N$ whose coefficients depend on $\{ \log _q N \}$. The coefficient of $(\log _q N)^m$ comes from $$v_{m-1}'(I-A_0)\left( {\begin{array}{c}\ell \\ m\end{array}}\right) $$, therefore, this coefficient is

$$\begin{aligned} \frac{1}{m!}v_{m-1}'(I-A_0)=\frac{1}{m!}w(C-I)^{m-1}(I-A_0)=w\vartheta _m. \end{aligned}$$

The additional factor $T^{-1}(I-D)T$ in $\vartheta _m$ has been introduced in order to annihilate generalised eigenvectors to other eigenvalues. By construction of *K*, we have $wK=0$. Thus we have shown (12.5) for $\lambda =1$, too.

*Lifting the Second Summand.* For later use—at this point, this may seem to be quite artificial—we set $\Psi ^{(2)}_{k}=\Phi ^{(2)}_{k}\circ {\mathsf {lval}}$. As $\Phi ^{(2)}_{k}$ is continuously differentiable, it is Lipschitz continuous on [0, 1]. As ${\mathsf {lval}}$ is also Lipschitz continuous, so is $\Psi _k^{(2)}$.

*First Summand* We now turn to $wF_1(N)$. To explain our plan, assume that *w* is in fact an eigenvector. Then $wF_1(N)=\sum _{0\le j<\ell }\lambda ^j wB_{r_j}A_{r_{j+1}}\ldots A_{r_{\ell -1}}$. For $|\lambda |\le R$, it will be rather easy to see that the result holds. Otherwise, we will factor out $\lambda ^\ell$ and write the sum as $wF_1(N)=\lambda ^{\ell } \sum _{0\le j<\ell }\lambda ^{-(\ell -j)}wB_{r_j}A_{r_{j+1}}\ldots A_{r_{\ell -1}}$. We will then reverse the order of summation and extend the sum to an infinite sum, which will be represented by periodic fluctuations. The difference between the finite and the infinite sums will be absorbed by the error term. The periodic fluctuations will be defined on the infinite product space $\Omega$.

We now return to the general case of a generalised eigenvector *w* and the actual proof. If $\lambda =0$, we certainly have $|\lambda |\le R$ and we are in one of the first two cases of this theorem. Furthermore, we have $wC^j=0$ for $j\ge m$, thusby using (7.1). Together with (12.5), the result follows.

From now on, we may assume that $\lambda \ne 0$. By using (12.3), we have

12.7 $$\begin{aligned} wF_1(N)=\sum _{0\le j<\ell } \lambda ^j\biggl (\sum _{0\le k<m}j^kv_k \biggr )B_{r_j} A_{r_{j+1}}\ldots A_{r_{\ell -1}}. \end{aligned}$$

We first consider the case that $|\lambda |<R$ [corresponding to Theorem B, (1)]. We getwhere (7.1) was used. Together with (12.5), the result follows.

Next, we consider the case where $|\lambda |=R$ [Theorem B, (2)]. In that case, we getAgain, the result follows.

From now on, we may assume that $|\lambda |>R$. We set $Q:=|\lambda |/R$ and note that $1<Q\le q$ by assumption and Lemma 12.1. We claim that there are continuous functions $\Psi ^{(1)}_{k}$ on $\Omega$ for $0\le k<m$ such that12.8and such that12.9when the first *j* entries of ${\mathbf {x}}$ and ${\mathbf {x}}'\in \Omega$ coincide.

Write $N=q^{\ell -1+\{ \log _q N \}}$ and let ${\mathbf {x}}={\mathsf {reprq}}(\{ \log _q N \})$, i.e., ${\mathbf {x}}$ is the *q*-ary expansion of $q^{\{ \log _q N \}}=N/q^{\ell -1}\in [1, q)$ ending on infinitely many zeros. This means that $x_j=r_{\ell -1-j}$ for $0\le j<\ell$ and $x_j=0$ for $j\ge \ell$. Reversing the order of summation in (12.7) yields

$$\begin{aligned} wF_1(N)=\lambda ^{\ell -1}\sum _{0\le j<\ell }\lambda ^{-j}\biggl (\sum _{0\le k<m}(\ell -1-j)^kv_k \biggr )B_{x_j} A_{x_{j-1}}\ldots A_{x_0}. \end{aligned}$$

For $j\ge \ell$, we have $x_j=0$ and therefore $B_{x_j}=0$. Thus we may extend the sum to run over all $j\ge 0$, i.e.,

$$\begin{aligned} wF_1(N)=\lambda ^{\ell -1}\sum _{j\ge 0}\lambda ^{-j}\biggl (\sum _{0\le k<m}(\ell -1-j)^kv_k \biggr )B_{x_j} A_{x_{j-1}}\ldots A_{x_0}. \end{aligned}$$

We insert $\ell -1=\log _q N - \{ \log _q N \}$ and obtainwhere

$$\begin{aligned} G_1(L, {\mathbf {x}})&=\lambda ^{-{\mathsf {lval}}({\mathbf {x}})}\sum _{j\ge 0}\lambda ^{-j}\biggl (\sum _{0\le k<m}(L-{\mathsf {lval}}({\mathbf {x}}) - j)^kv_k \biggr )B_{x_j} A_{x_{j-1}}\ldots A_{x_0}\\&=\lambda ^{-{\mathsf {lval}}({\mathbf {x}})}\sum _{j\ge 0}\lambda ^{-j}\biggl (\sum _{\begin{array}{c} 0\le a,\ 0\le r,\ 0\le s\\ a+r+s<m \end{array}}L^a (-\,j)^r \left( {\begin{array}{c}a+r+s\\ a, r, s\end{array}}\right) \\&\quad \times \bigl (-\,{\mathsf {lval}}({\mathbf {x}})\bigr )^{s}v_{a+r+s} \biggr )B_{x_j} A_{x_{j-1}}\ldots A_{x_0} \end{aligned}$$

for $L\in {\mathbb {R}}$ and ${\mathbf {x}}\in \Omega$. Note that in contrast to $G_2$, the second argument of $G_1$ is an element of $\Omega$ instead of ${\mathbb {R}}$. Collecting $G_1(L, {\mathbf {x}})$ by powers of *L*, we get

$$\begin{aligned} G_1(L, {\mathbf {x}}) = \sum _{0\le k<m} L^k \Psi ^{(1)}_{ k}({\mathbf {x}}) \end{aligned}$$

wherefor functions

$$\begin{aligned} \psi _{kr}(\nu )=\lambda ^{-\nu } (-\,1)^r\sum _{0\le s<m-k-r} \left( {\begin{array}{c}k+r+s\\ k, r, s\end{array}}\right) (-\,\nu )^{s}v_{k+r+s} \end{aligned}$$

which are continuously differentiable and therefore Lipschitz continuous on the unit interval. This shows (12.8). For $k=m-1$, only summands with $r=s=0$ occur, thus

12.10 $$\begin{aligned} \Psi _{m-1}^{(1)}({\mathbf {x}})=\sum _{j\ge 0}\lambda ^{-j-{\mathsf {lval}}({\mathbf {x}})}v_{m-1}B_{x_j}A_{x_{j-1}}\ldots A_{x_0}. \end{aligned}$$

Note that $\Psi ^{(1)}_{ k}({\mathbf {x}})$ is majorised byaccording to (7.1). We now prove (12.9). So let ${\mathbf {x}}$ and ${\mathbf {x}}'$ have a common prefix of length *i*. Consider the summand of $\Psi ^{(1)}_k({\mathbf {x}})$ with index *j*. First consider the case that $j<i$. For all *r*, we havedue to Lipschitz continuity of $\psi _{kr}\circ {\mathsf {lval}}$. As the matrix product $A_{x_{j-1}} \ldots A_{x_0}$ is the same for ${\mathbf {x}}$ and ${\mathbf {x}}'$, the difference with respect to this summand is bounded byThus the total contribution of all summands with $j<i$ is . Any summand with $j \ge i$ is bounded by , which leads to a total contribution of . Adding the two bounds leads to a bound of , as requested.

*Descent.* As we have defined the periodic fluctuations $\Psi ^{(1)}_k$ on the infinite product space $\Omega$, we now need to prove that the periodic fluctuation descends to a periodic fluctuation on the unit interval. To do so, we will verify that the values of the fluctuation coincide whenever sequences in the infinite product space correspond to the same real number in the interval.

By setting $\Psi _k({\mathbf {x}})=\Psi ^{(1)}_{k}({\mathbf {x}})+\Psi ^{(2)}_{k}({\mathbf {x}})$, we obtain12.11and12.12whenever ${\mathbf {x}}$ and ${\mathbf {x}}'\in \Omega$ have a common prefix of length *j*.

It remains to show that $\Psi _k({\mathbf {x}})=\Psi _k({\mathbf {x}}')$ holds whenever ${\mathsf {lval}}({\mathbf {x}})={\mathsf {lval}}({\mathbf {x}}')$ or ${\mathsf {lval}}({\mathbf {x}})=0$ and ${\mathsf {lval}}({\mathbf {x}}')=1$.

Choose ${\mathbf {x}}$ and ${\mathbf {x}}'$ such that one of the above two conditions on ${\mathsf {lval}}$ holds and such that $x_j=0$ for $j\ge j_0$ and $x'_j=q-1$ for $j\ge j_0$. Be aware that now the prefixes of ${\mathbf {x}}$ and ${\mathbf {x}}'$ of length $j_0$ do not coincide except for the trivial case $j_0=0$.

Fix some $j\ge j_0$ and set ${\mathbf {x}}''$ to be the prefix of ${\mathbf {x}}'$ of length *j*, followed by infinitely many zeros. Note that we have $q^{{\mathsf {lval}}({\mathbf {x}}'')}=q^{{\mathsf {lval}}({\mathbf {x}}')}-q^{-(j-1)}$. Set $n=q^{j-1+{\mathsf {lval}}({\mathbf {x}}'')}$. By construction, we have $n+1=q^{j-1+{\mathsf {lval}}({\mathbf {x}})+[ {\mathsf {lval}}({\mathbf {x}})=0 ]}$. This implies ${\mathsf {reprq}}(\{ \log _q n \})={\mathbf {x}}''$ and ${\mathsf {reprq}}(\{ \log _q(n+1) \})={\mathbf {x}}$. Taking the difference of (12.11) for $n+1$ and *n* yields

$$\begin{aligned} wf(n)= & {} (n+1)^{\log _q \lambda } \sum _{0\le k<m}\bigl (\log _q (n+1)\bigr )^k \Psi _k({\mathbf {x}}) - n^{\log _q \lambda } \sum _{0\le k<m} (\log _q n)^k \Psi _k({\mathbf {x}}'') \\&+\big ((\log _q(n+1))^m-(\log _q n)^m\big )w\vartheta _m. \end{aligned}$$

We estimate $n+1$ as  and get12.13We have  by (6.2) and (7.1). By (12.12),which is used below to replace ${\mathbf {x}}''$ by ${\mathbf {x}}'$. Inserting these estimates in (12.13) and dividing by $n^{\log _q \lambda }$ yields12.14Note that $\Psi _k({\mathbf {x}}')-\Psi _k({\mathbf {x}})$ does not depend on *j*. Now we let *j* (and therefore *n*) tend to infinity. We see that (12.14) can only remain true if $\Psi _k({\mathbf {x}}')=\Psi _k({\mathbf {x}})$ for $0\le k<m$, which we had set out to show.

Therefore, $\Psi _k$ descends to a continuous function $\Phi _k$ on [0, 1] with $\Phi _k(0)=\Phi _k(1)$; thus $\Phi _k$ can be extended to a 1-periodic continuous function.

*Hölder Continuity.* We will now prove Hölder continuity. As the fluctuations have been defined on the infinite product space $\Omega$, we will basically have to prove Hölder continuity there. The difficulty will be that points in the unit interval which are close to each other there may have drastically different *q*-ary expansions, thus correspond to drastically different points in the infinite product space $\Omega$. To circumvent this problem, the interval between the two points will be split into two parts.

We first claim that for $0\le y<y'''<1$, we have12.15as $y'''\rightarrow y$. To prove this, let ${\mathbf {x}}:={\mathsf {reprq}}(y)$ and ${\mathbf {x}}''':={\mathsf {reprq}}(y''')$. Let $\ell$ be the length of the longest common prefix of ${\mathbf {x}}$ and ${\mathbf {x}}'''$ and choose $j\ge 0$ such that $q^{-j}\le q^{y'''}-q^y< q^{-j+1}$. We define ${\mathbf {x}}'$ and ${\mathbf {x}}''\in \Omega$ such thatand set $y':={\mathsf {lval}}({\mathbf {x}}')$ and $y'':={\mathsf {lval}}({\mathbf {x}}'')$. As ${\mathsf {lval}}({\mathbf {x}})=y<y'''={\mathsf {lval}}({\mathbf {x}}''')$, we have $x'''_\ell >x_\ell$. We conclude that $y\le y'=y''\le y'''$. Therefore,

$$\begin{aligned} q^{y'}-q^{y} \le q^{y'''}-q^{y}< q^{-j+1}, \end{aligned}$$

so in view of the fact that each entry of ${\mathbf {x}}'$ is greater or equal than the corresponding entry of ${\mathbf {x}}$, the expansions ${\mathbf {x}}$ and ${\mathbf {x}}'$ must have a common prefix of length *j*. Similarly, the expansions ${\mathbf {x}}''$ and ${\mathbf {x}}'''$ must have a common prefix of length *j*. Thus (12.12) implies thatNoting that  leads to (12.15).

In order to prove Hölder continuity with exponent $\alpha <\log _q Q$, we first note that Lipschitz-continuity of $y\mapsto q^y$ on the interval [0, 1] shows that (12.15) impliesThis can then easily be extended to arbitrary reals $y<y'''$ by periodicity of $\Phi _k$ because it is sufficient to consider small $y'''-y$ and the interval may be subdivided at an integer between *y* and $y'''$.

*Constant Dominant Fluctuation.* To finally prove the final assertion on constant fluctuations, we will have to inspect the explicit expression for the fluctuations using the additional assumption.

Under the additional assumption that the vector $w(C-I)^{m-1}=v_{m-1}'$ is a left eigenvector to all matrices $A_0, \ldots , A_{q-1}$ associated with the eigenvalue 1, the same holds for $v_{m-1}$ by (12.4). Then $v_{m-1}$ is also a left eigenvector of *C* associated with the eigenvalue *q*. In particular, $\lambda =q\ne 1$.

We can compute $\Phi _{m-1}^{(2)}(\nu )$ using (12.6). As $v_{m-1}\in W_{q}$, we have $v_{m-1}C=v_{m-1}C'$ by definition of $C'$ (see Sect. 7.3) which implies that $v_{m-1}(I-C')^{-1}=\frac{1}{1-q}v_{m-1}$. As $v_{m-1}(I-A_0)=0$ by assumption, we conclude that $\Phi _{m-1}^{(2)}(\nu )=0$ in this case.

We use (12.10) to compute $\Psi _{m-1}^{(1)}({\mathbf {x}})$. By assumption, $v_{m-1}B_{x_j}=x_j v_{m-1}$ which implies that

$$\begin{aligned} \Psi _{m-1}^{(1)}({\mathbf {x}}) = q^{-{\mathsf {lval}}({\mathbf {x}})} \biggl (\sum _{j\ge 0}q^{-j}x_j\biggr ) v_{m-1} =q^{-{\mathsf {lval}}({\mathbf {x}})}q^{{\mathsf {lval}}({\mathbf {x}})}v_{m-1}=v_{m-1} \end{aligned}$$

by definition of ${\mathsf {lval}}$.

Together with (12.4), we obtain the assertion. $\square$

### Proof of Theorem C

#### Proof of Theorem C

We denote the rows of *T* as $w_1, \ldots , w_d$ and the columns of $T^{-1}$ by $t_1, \ldots , t_d$. Thus $\sum _{1 \le j \le d} t_jw_j=I$ and $w_j$ is a generalised left eigenvector of *C* of some rank $m_j$ corresponding to some eigenvalue $\lambda _j\in \sigma (C)$. Theorem B and the fact that there are no eigenvalues of *C* of absolute value between $\rho$ and *R* then immediately imply thatfor some 1-periodic Hölder continuous functions $\Psi _{jk}$ with exponent less than $\log _q|\lambda _j |/R$. The first summand *K* as well as the error term already coincide with the result stated in the theorem. From Sect. 7.3 we recall that $w_j\vartheta _{m_j}=0$ for $\lambda _j\ne 1$.

We set

$$\begin{aligned} \Phi _{\lambda k}(u):=\sum _{\begin{array}{c} 1\le j\le d\\ \lambda _j=\lambda \\ k<m_j \end{array}} \bigl (t_j\Psi _{jk}(u) +[ \lambda =1 ][ m_j=k ]t_jw_j\vartheta _{m_j}\bigr ) \end{aligned}$$

for $\lambda \in \sigma (C)$ with $|\lambda |>\rho$ and $0\le k<m(\lambda )$.

Then we still have to account for

12.16 $$\begin{aligned} (\log _q N)^{m(1)}\sum _{\begin{array}{c} 1\le j\le d\\ \lambda _j=1\\ m_j=m(1) \end{array}}t_jw_j\vartheta _{m(1)}. \end{aligned}$$

The factor $(C-I)^{m(1)-1}$ in the definition of $\vartheta _{m(1)}$ implies that $w_j\vartheta _{m(1)}$ vanishes unless $\lambda _j=1$ and $m_j=m(1)$. Therefore, the sum in (12.16) equals $\vartheta$. $\square$

## Meromorphic Continuation of the Dirichlet Series: Proof of Theorem D

For future use, we state an estimate for the binomial coefficient. Unsurprisingly, it is a consequence of a suitable version of Stirling’s formula. Alternatively, it can be seen as the most basic case of Flajolet and Odlyzko’s singularity analysis [19, Proposition 1], where uniformity in *s* is easily checked.

### Lemma 13.1

Let $$k\in {\mathbb {Z}}$$, $$k\ge 0$$. Then

13.1 $$\begin{aligned} \left| {\left( {\begin{array}{c}-s\\ k\end{array}}\right) }\right| \sim \frac{1}{|\Gamma (s) |}k^{\mathfrak {R}s-1} \end{aligned}$$

uniformly for *s* in a compact subset of ${\mathbb {C}}$ and $k\rightarrow \infty$.

### Proof

By [26, (5.14)] (“negating the upper index”), we rewrite the binomial coefficient as

$$\begin{aligned} \left( {\begin{array}{c}-s\\ k\end{array}}\right) =(-\,1)^{k}\left( {\begin{array}{c}s+k-1\\ k\end{array}}\right) =\frac{(-\,1)^k}{\Gamma (s)}\frac{\Gamma (s+k)}{\Gamma (k+1)}. \end{aligned}$$

Thus (13.1) follows by [36, 5.11.12] (which is an easy consequence of Stirling’s formula for the Gamma function). $\square$

### Proof of Lemma 6.3

We have13.2for $\mathfrak {R}s>\log _q R'+ 1$. We note thatTherefore,and the series converges for $\mathfrak {R}s>\log _q R'$. As this holds for all $R'>\rho$, we obtain  as $|\mathfrak {I}s |\rightarrow \infty$ uniformly for $\log _q \rho + \delta \le \mathfrak {R}s \le \log _q \rho +\delta +1$. In the language of [27, § 3.3],  has order at most 1 for $\log _q \rho + \delta \le \mathfrak {R}s \le \log _q \rho +\delta +1$. As $\log _q \rho +\delta +1$ is larger than the abscissa of absolute convergence of , it is clear that  for $\mathfrak {R}s=\log _q \rho +\delta +1$, i.e.,  has order at most 0 for $\mathfrak {R}s=\log _q \rho +\delta +1$. By Lindelöf’s theorem (see [27, Theorem 14]), we conclude that  for $\log _q \rho + \delta \le \mathfrak {R}s\le \log _q \rho +\delta +1$.

For $\mathfrak {R}s > \log _q R' + 1$, we may rewrite (13.2) using the binomial series as13.3Switching the order of summation was legitimate becausefor $\mathfrak {R}s+k>\log _q R'+1$ and Lemma 13.1 imply absolute and uniform convergence for *s* in a compact set. Noting that the previous arguments hold again for all $R'>\rho$ and that the inner sum in (13.3) is ${\mathcal {D}}(s+k)$ completes the proof. $\square$

### Proof of Theorem D

As  by (6.2) and (7.1), the Dirichlet series ${\mathcal {F}}_{n_0}(s) = \sum _{n \ge n_0} n^{-s} f(n)$ (see Sect. 6.2) converges absolutely and uniformly on compact sets for $\mathfrak {R}s>\log _q R+1$. As this holds for all $R>\rho$, i.e., does not depend on our particular (cf.  Sect. 6.2) choice of $R>\rho$, this convergence result holds for $\mathfrak {R}s>\log _q \rho +1$.

We use (6.1) and Lemma 6.3 (including its notation) to rewrite ${\mathcal {F}}_{n_0}$ as

$$\begin{aligned} {\mathcal {F}}_{n_0}(s)&= \sum _{n_0 \le n< qn_0}n^{-s}f(n) + \sum _{0 \le r< q} \sum _{n\ge n_0} (qn+r)^{-s} f(qn+r)\\&= \sum _{n_0 \le n< qn_0} n^{-s}f(n) + q^{-s} \sum _{0 \le r< q} A_r \sum _{n\ge n_0} \Bigl (n+\frac{r}{q}\Bigr )^{-s} f(n)\\&= \sum _{n_0 \le n < qn_0} n^{-s}f(n) + q^{-s}C{\mathcal {F}}_{n_0}(s) + {\mathcal {H}}_{n_0}(s) \end{aligned}$$

withfor $$\mathfrak {R}s>\log _q R+ 1$$. Thus

13.4 $$\begin{aligned} \bigl (I-q^{-s}C\bigr ){\mathcal {F}}_{n_0}(s) = \sum _{n_0 \le n < qn_0}n^{-s}f(n)+{\mathcal {H}}_{n_0}(s) \end{aligned}$$

for $\mathfrak {R}s>\log _q R+ 1$. By Lemma 6.3 we have  for $\log _q \rho + \delta \le \mathfrak {R}s\le \log _q \rho +\delta +1$. Rewriting the expression for ${\mathcal {H}}_{n_0}(s)$ using the binomial series (see Lemma 6.3 again) yields

$$\begin{aligned} {\mathcal {H}}_{n_0}(s) = q^{-s}\sum _{0 \le r < q} A_r \sum _{k\ge 1}\left( {\begin{array}{c}-s\\ k\end{array}}\right) \Bigl (\frac{r}{q}\Bigr )^k {\mathcal {F}}_{n_0}(s+k). \end{aligned}$$

Combining this with (13.4) yields the expression (6.4) for ${\mathcal {G}}_{n_0}$.

Solving (6.3) for ${\mathcal {F}}_{n_0}$ yields the meromorphic continuation of ${\mathcal {F}}_{n_0}(s)$ to $\mathfrak {R}s>\log _q R$ (and thus to $\mathfrak {R}s>\log _q \rho$) with possible poles where $q^s$ is an eigenvalue of *C*. As long as $q^s$ keeps a fixed positive distance $\delta$ from the eigenvalues, the bound for ${\mathcal {G}}_{n_0}$ (coming from the bound for ${\mathcal {H}}_{n_0}$) carries over to a bound for ${\mathcal {F}}_{n_0}$, i.e., (6.5).

To estimate the order of the poles, let *w* be generalised left eigenvector of rank *m* of *C* corresponding to an eigenvalue $\lambda$ with $|\lambda |>R$. We claim that $w{\mathcal {F}}_{n_0}(s)$ has a pole of order at most *m* at $s=\log _q \lambda +\chi _k$ and no other poles for $\mathfrak {R}s>\log _q R$. We prove this by induction on *m*.

Set $v:=w(C-\lambda I)$. By definition, $v=0$ or *v* is a generalised eigenvector of rank $m-1$ of *C*. By induction hypothesis, $v{\mathcal {F}}_{n_0}(s)$ has a pole of order at most $m-1$ at $s=\log _q \lambda +\chi _k$ for $k\in {\mathbb {Z}}$ and no other poles for $\mathfrak {R}s>\log _q R$.

Multiplying (6.3) by *w*, inserting the definition of *v* and reordering the summands yields

$$\begin{aligned} \bigl (1 - q^{-s}\lambda \bigr )w{\mathcal {F}}_{n_0}(s) = q^{-s}v {\mathcal {F}}_{n_0}(s) + w{\mathcal {G}}_{n_0}(s). \end{aligned}$$

The right-hand side has a pole of order at most $m-1$ at $\log _q \lambda +\chi _k$ for $k\in {\mathbb {Z}}$ and $1-q^{-s}\lambda$ has a simple zero at the same places. This proves the claim. $\square$

## Fourier Coefficients: Proof of Theorem E

In contrast to the rest of this paper, this section does not directly relate to a regular sequence but gives a general method to derive Fourier coefficients of fluctuations.

### Pseudo-Tauberian Theorem

In this section, we generalise the pseudo-Tauberian argument by Flajolet, Grabner, Kirschenhofer, Prodinger and Tichy [18, Proposition 6.4]. The basic idea is that for a 1-periodic Hölder-continuous function $\Phi$ and $\gamma \in {\mathbb {C}}$, there is a 1-periodic continuously differentiable function $\Psi$ such thatand there is a straight-forward relation between the Fourier coefficients of $\Phi$ and the Fourier coefficients of $\Psi$. This relation exactly corresponds to the additional factor $s+1$ when transitioning from the zeroth order Mellin–Perron formula to the first order Mellin–Perron formula.

In contrast to [18, Proposition 6.4], we allow for an additional logarithmic factor, have weaker growth conditions on the Dirichlet series and quantify the error. We also extend the result to all complex $\gamma$. The generalisation from $q=2$ there to our real $q>1$ is trivial.

#### Proposition 14.1

Let $\gamma \in {\mathbb {C}}$ and $q>1$ be a real number, *m* be a positive integer, $\Phi _0, \ldots , \Phi _{m-1}$ be 1-periodic Hölder continuous functions with exponent $\alpha >0$, and $0<\beta <\alpha$. Then there exist continuously differentiable functions $\Psi _{-1}$, $\Psi _{0}, \ldots , \Psi _{m-1}$, periodic with period 1, and a constant *c* such that14.1for integers $N\rightarrow \infty$.

Denote the Fourier coefficients of $\Phi _j$ and $\Psi _j$ by $\varphi _{j\ell }:=\int _0^1\Phi _j(u)\exp (-\,2\ell \pi i u)\, {\mathrm {d}}u$ and $\psi _{j\ell }:=\int _0^1\Psi _j(u)\exp (-\,2\ell \pi i u)\, {\mathrm {d}}u$, respectively. Then the corresponding generating functions fulfil14.2for $\ell \in {\mathbb {Z}}$ and $Z\rightarrow 0$.

If $q^{\gamma +1}\ne 1$, then $\Psi _{-1}$ vanishes.

#### Remark 14.2

Note that the constant *c* is absorbed by the error term if $\mathfrak {R}\gamma +1>\alpha$, in particular if $\mathfrak {R}\gamma >0$. Therefore, this constant does not occur in the article [18].

#### Remark 14.3

The factor $\gamma +1+\frac{2\ell \pi i}{\log q} + Z$ in (14.2) will turn out to correspond exactly to the additional factor $s+1$ in the first order Mellin–Perron summation formula with the substitution $s=\gamma +\frac{2\ell \pi i}{\log q}+ Z$ such that the local expansion around the pole in $s=\gamma +\frac{2\ell \pi i}{\log q}$ of the Dirichlet generating function is conveniently written as a Laurent series in *Z*. See the proof of Theorem E for details.

Before actually proving Proposition 14.1, we give an outline.

#### Overview of the Proof of Proposition 14.1

We start with the left-hand side of (14.1) and split the range of summation according to $\lfloor \log _q n \rfloor$, thereby, in terms of our periodic functions, split after each period. We then use periodicity of the $\Phi _j$ and collect terms. This results in Riemann sums which converge to the corresponding integrals. Therefore, we can approximate these sums by the integrals.

More rewriting constructs and reveals the functions $\Psi _j$ [of the right-hand side of (14.1)]: these functions are basically defined via the above mentioned integral. We then show that these functions are indeed periodic and that their Fourier coefficients relate to the Fourier coefficients of the $\Phi _j$. The latter is done by a direct computation of the integrals defining these coefficients.

For this proof, we use an approach via exponential generating functions. This reduces the overhead for dealing with the logarithmic factors $(\log n)^k$ in (14.1) such that we can essentially focus on the case $m=1$. The resulting formula (14.1) follows by extracting a suitable coefficient of this power series.

There is another benefit of the generating function approach: this formulation allows to easily translate the relation between the Fourier coefficients here to the additional factors occurring when transitioning to higher order Mellin–Perron summation formulæ, in particular the factor $s+1$ in the first order Mellin–Perron summation. $\square$

#### Proof of Proposition 14.1

We split the proof into six parts.

*Notations.* We start by defining quantities that are used through the whole proof.

Without loss of generality, we assume that $q^{\mathfrak {R}\gamma +1}\ne q^{\alpha }$: otherwise, we slightly decrease $\alpha$ keeping the inequality $\beta <\alpha$ intact. We use the abbreviations $\Lambda :=\lfloor \log _q N \rfloor$, $\nu :=\{ \log _q N \}$, i.e., $N=q^{\Lambda +\nu }$. We use the generating functionsfor $0\le u\le 1$ and $0<|Z |<2r$ where $r>0$ is chosen such that $r<(\alpha -\beta )/2$ and such that $Q(Z)\ne 1$ and $|Q(Z) |\ne q^{\alpha }$ for these *Z*. (The condition $Z\ne 0$ is only needed for the case $q^{1+\gamma }=1$.) We will stick to the above choice of *r* and restrictions for *Z* throughout the proof.

It is easily seen that the left-hand side of (14.1) equals $[Z^{m-1}]L(N, Z)$, where $[Z^{m-1}]$ denotes extraction of the coefficient of $Z^{m-1}$.

*Approximation of the Sum by an Integral.* We will now rewrite *L*(*N*, *Z*) so that its shape is that of a Riemann sum, therefore enabling us to approximate it by an integral.

Splitting the range of summation with respect to powers of *q* yieldsWe write $n=q^px$ (or $n=q^\Lambda x$ for the second sum), use the periodicity of $\Phi$ in *u* and getThe inner sums are Riemann sums converging to the corresponding integrals for $p\rightarrow \infty$. We setIt will be convenient to change variables $x=q^w$ in *I*(*u*, *Z*) to get14.3We define the error $\varepsilon _p(u, Z)$ byAs the sum and the integral are both analytic in *Z*, their difference $\varepsilon _p(u, Z)$ is analytic in *Z*, too. We bound $\varepsilon _{p}(u, Z)$ by the difference of upper and lower Darboux sums (step size $q^{-p}$) corresponding to the integral *I*(*u*, *Z*): On each interval of length $q^{-p}$, the maximum and minimum of a Hölder continuous function can differ by at most . As the integration interval as well as the range for *u* and *Z* are finite, this translates to the bound  as $p\rightarrow \infty$ uniformly in $0\le u\le 1$ and $|Z |<2r$. This results in

$$\begin{aligned} L(N, Z)= & {} I(1, Z)\sum _{0\le p<\Lambda }Q(Z)^p + \sum _{0\le p<\Lambda }Q(Z)^p \varepsilon _{p}(1, Z) \\&+ I(\nu , Z)\,Q(Z)^{\Lambda } + Q(Z)^\Lambda \varepsilon _{\Lambda }(\nu , Z). \end{aligned}$$

If $|Q(Z) |/q^\alpha =q^{\mathfrak {R}\gamma +1 + \mathfrak {R}Z -\alpha }<1$, i.e., $\mathfrak {R}\gamma +\mathfrak {R}Z<\alpha -1$, the second sum involving the integration error converges absolutely and uniformly in *Z* for $\Lambda \rightarrow \infty$ to some analytic function $c'(Z)$; therefore, we can replace the second sum by  in this case. If $\mathfrak {R}\gamma + \mathfrak {R}Z>\alpha -1$, then the second sum is . By our choice of *r*, the case $\mathfrak {R}\gamma +\mathfrak {R}Z=\alpha -1$ cannot occur. So in any case, we may write the second sum as  by our choice of *r*. The last summand involving $\varepsilon _{\Lambda }(\nu , Z)$ is absorbed by the error term of the second summand. Note that the error term is uniform in *Z* and, by its construction, analytic in *Z*.

Thus we end up with14.4where

14.5 $$\begin{aligned} S(N, Z):=I(1, Z)\sum _{0\le p<\Lambda }Q(Z)^p+I(\nu , Z)Q(Z)^\Lambda . \end{aligned}$$

It remains to rewrite *S*(*N*, *Z*) in the form required by (14.1). We emphasise that we will compute *S*(*N*, *Z*) exactly, i.e., no more asymptotics for $N\rightarrow \infty$ will play any rôle.

*Construction of*$\Psi$. We will now rewrite the expression *S*(*N*, *Z*) such that the generating function $\Psi$ [i.e., the fluctuations of the right-hand side of (14.1)] appears. After this, we will gather properties of $\Psi$ including properties of its Fourier coefficients.

We rewrite (14.5) asWe replace $\Lambda$ by $\log _q N - \nu$ and useto get14.6with14.7*Periodic Extension of*$\Psi$. A priori, it is not clear that the function $\Psi (u, Z)$ defined above can be extended to a periodic function (and therefore Fourier coefficients can be computed later on). The aim now is to show that it is possible to do so.

It is obvious that  is continuously differentiable in $u\in [0, 1]$. We havebecause $I(0, Z)=0$ by (14.3). The derivative of  with respect to *u* iswhich implies thatWe can therefore extend  to a 1-periodic continuously differentiable function in *u* on ${\mathbb {R}}$.

*Fourier Coefficients of*$\Psi$ Knowing that $\Psi$ is a periodic function, we can now head for its Fourier coefficients and relate them to those of $\Phi$.

By using equations (14.7) and (14.3), $Q(Z)=q^{\gamma +1+Z}$, and $\exp (-\,2\ell \pi iu)=q^{-\chi _\ell u}$ with $\chi _\ell =\frac{2\pi i\ell }{\log q}$, we now express the Fourier coefficients of  in terms of those of  byThe second and third summands cancel, and we get14.8*Extracting Coefficients.* So far, we have proven everything in terms of generating functions. We now extract the coefficients of these power series which will give us the result claimed in Proposition 14.1.

By (14.7),  is analytic in *Z* for $0<|Z |<2r$. If $q^{\gamma +1}\ne 1$, then it is analytic in $Z=0$, too. If $q^{\gamma +1}=1$, then (14.7) implies that  might have a simple pole in $Z=0$. Note that all other possible poles have been excluded by our choice of *r*. For $j\ge -1$, we writeand use Cauchy’s formula to obtainThis and the properties of  established above imply that $\Psi _j$ is a 1-periodic continuously differentiable function.

Inserting (14.6) in (14.4) and extracting the coefficient of $Z^{m-1}$ using Cauchy’s theorem and the analyticity of the error in *Z* yields (14.1) with . Rewriting (14.8) in terms of $\Psi _j$ and $\Phi _j$ leads to (14.2). Note that we have to add  in (14.2) to compensate the fact that we do not include $\psi _{j\ell }$ for $j\ge m$.

$$\square $$

We prove a uniqueness result.

#### Lemma 14.4

Let *m* be a positive integer, $q>1$ be a real number, $\gamma \in {\mathbb {C}}$ such that $\gamma \notin \frac{2\pi i}{\log q}{\mathbb {Z}}$, $c\in {\mathbb {C}}$, and $\Psi _0, \ldots , \Psi _{m-1}$ and $\Xi _0, \ldots , \Xi _{m-1}$ be 1-periodic continuous functions such that14.9for integers $N\rightarrow \infty$. Then $\Psi _k=\Xi _k$ for $0\le k<m$.

#### Proof

If $\mathfrak {R}\gamma <0$ and $c\ne 0$, then (14.9) is impossible as the growth of the right-hand side of the equation is larger than that on the left-hand side. So we can exclude this case from further consideration. We proceed indirectly and choose *k* maximally such that $\Xi _k\ne \Psi _k$. Dividing (14.9) by $(\log _q N)^k$ yields14.10for $N\rightarrow \infty$. Let $0< u<1$ and set $N_j=\lfloor q^{j+u} \rfloor$. We clearly have $\lim _{j\rightarrow \infty } N_j=\infty$. Then

$$\begin{aligned} j+u + \log _q(1-q^{-j-u}) = \log _q(q^{j+u}-1)\le \log _q N_j \le j+u. \end{aligned}$$

We define $\nu _j:=\log _q N_j-j-u$ and see that  for $j\rightarrow \infty$, i.e., $\lim _{j\rightarrow \infty } \nu _j = 0$. This implies that $\lim _{j\rightarrow \infty }\{ \log _q N_j \}=u$ and therefore

$$\begin{aligned} \lim _{j\rightarrow \infty }(\Xi _k-\Psi _k)(\log _q N_j)=\lim _{j\rightarrow \infty }(\Xi _k-\Psi _k)(\{ \log _q N_j \})=\Xi _k(u)-\Psi _k(u). \end{aligned}$$

Setting $N=N_j$ in (14.10) and letting $j\rightarrow \infty$ shows that

14.11 $$\begin{aligned} \Xi _k(u)-\Psi _k(u) = \lim _{j\rightarrow \infty }cN_j^{-\gamma }[ k=0 ]. \end{aligned}$$

If $k\ne 0$ or $\mathfrak {R}\gamma >0$, we immediately conclude that $\Xi _k(u)-\Psi _k(u)=0$. If $\mathfrak {R}\gamma <0$ we have $c=0$, which again implies that $\Xi _k(u)-\Psi _k(u)=0$.

Now we assume that $\mathfrak {R}\gamma =0$ and $k=0$. We set $\beta :=-\frac{\log q}{2\pi i}\gamma$, which implies that $N^{-\gamma }=\exp (2\pi i \beta \log _q N)$. We choose sequences $(r_\ell )_{\ell \ge 1}$ and $(s_\ell )_{\ell \ge 1}$ such that $\lim _{\ell \rightarrow \infty }s_\ell =\infty$ and $\lim _{\ell \rightarrow \infty }|s_\ell \beta - r_\ell |=0$: For rational $\beta =r/s$, we simply take $r_\ell =\ell r$ and $s_\ell =\ell s$, and for irrational $\beta$, we consider the sequence of convergents $(r_\ell /s_\ell )_{\ell \ge 1}$ of the continued fraction of $\beta$ and the required properties follow from the theory of continued fractions; see for example [28, Theorems 155 and 164]. By using $\log _q N_j = j+u+\nu _j$, we getThese two limits are distinct as $\beta \notin {\mathbb {Z}}$ by assumption. Thus $\lim _{j\rightarrow \infty }N_j^{-\gamma }$ does not exist. Therefore, (14.11) implies that $c=0$ and therefore $\Xi _k(u)-\Psi _k(u)=0$.

We proved that $\Xi _k(u)=\Psi _k(u)$ for $u\notin {\mathbb {Z}}$. By continuity, this also follows for all $u \in {\mathbb {R}}$; contradiction. $\square$

### Proof of Theorem E

We again start with an outline of the proof.

#### Overview of the Proof of Theorem E

The idea is to compute the repeated summatory function of *F* twice: On the one hand, we use the pseudo-Tauberian Proposition 14.1 to rewrite the right-hand side of (6.6) in terms of periodic functions $\Psi _{aj}$. On the other hand, we compute it using a higher order Mellin–Perron summation formula, relating it to the singularities of ${\mathcal {F}}$. More specifically, the expansions at the singularities of ${\mathcal {F}}$ give the Fourier coefficients of $\Psi _{aj}$. The Fourier coefficients of the functions $\Psi _{aj}$ are related to those of the functions $\Phi _j$ via (14.2). $\square$

And up next comes the actual proof.

#### Proof of Theorem E

*Initial observations and notations.* As $\Phi _j$ is Hölder continuous, its Fourier series converges by Dini’s criterion; see, for example, [40, p. 52].

For any sequence *g* on ${\mathbb {Z}}_{>0}$, we set $({\mathcal {S}}g)(N):=\sum _{1\le n< N}g(n)$. We set $A=1 + \max \{ \lfloor \eta \rfloor , 0 \}$. In particular, *A* is a positive integer with $A>\eta$.

*Asymptotic Summation.* We first compute the *A*th repeated summatory function ${\mathcal {S}}^A F$ of *F* (i.e., the $(A+1)$th repeated summatory function ${\mathcal {S}}^{A+1} f$ of the function *f*) by applying Proposition 14.1*A* times. This results in an asymptotic expansion involving new periodic fluctuations while keeping track of the relation between the Fourier coefficients of the original fluctuations and those of the new fluctuations.

A simple induction based on (6.6) and using Proposition 14.1 shows that there exist 1-periodic continuous functions $\Psi _{aj}$ for $a\ge 0$ and $-\,1\le j<m$ and some constants $c_{ab}$ for $0\le b<a$ such that14.12for integers $N\rightarrow \infty$. In fact, $\Psi _{0j}=\Phi _j$ for $0\le j<m$. For $a\ge 1$ and $-\,1\le j<m$, $\Psi _{aj}$ is continuously differentiable. Note that the case that $q^{\gamma +a+1}=1$ occurs for at most one $0\le a<A$, which implies that the number of non-vanishing fluctuations increases at most once in the application of Proposition 14.1. Also note that the assumption $\alpha >\mathfrak {R}\gamma -\gamma _0$ implies that the error terms arising in the application of Proposition 14.1 are absorbed by the error term stemming from (6.6).

We denote the corresponding Fourier coefficients by

$$\begin{aligned} \psi _{aj\ell }:=\int _{0}^1 \Psi _{aj}(u)\exp (-\,2\ell \pi i u)\,{\mathrm {d}}u \end{aligned}$$

for $0\le a\le A$, $-\,1\le j<m$, $\ell \in {\mathbb {Z}}$. By (14.2) the generating functions of the Fourier coefficients fulfilfor $0\le a<A$, $\ell \in {\mathbb {Z}}$ and $Z\rightarrow 0$. Iterating this recurrence yields14.13for $\ell \in {\mathbb {Z}}$ and $Z\rightarrow 0$.

*Explicit Summation.* We now compute ${\mathcal {S}}^{A+1} f$ explicitly with the aim of decomposing it into one part which can be computed by the *A*th order Mellin–Perron summation formula and another part which is smaller and can be absorbed by an error term.

Explicitly, we have

$$\begin{aligned} ({\mathcal {S}}^{a+1}f)(N) = \sum _{1\le n_1<n_2<\cdots<n_{a+1}<N}f(n_1) = \sum _{1\le n<N}f(n)\sum _{n<n_2<\cdots<n_{a+1}<N}1 \end{aligned}$$

for $0\le a \le A$. Note that we formally write the outer sum over the range $1\le n<N$ although the inner sum is empty (i.e., equals 0) for $n\ge N-a$; this will be useful later on. The inner sum counts the number of selections of *a* elements out of $\{ n+1,\ldots , N-1 \}$, thus we have

14.14 $$\begin{aligned} ({\mathcal {S}}^{a+1}f)(N) = \sum _{1\le n< N}\left( {\begin{array}{c}N-n-1\\ a\end{array}}\right) f(n)=\sum _{1\le n< N}\frac{1}{a!}(N-n-1)^{\underline{a}}f(n)\nonumber \\ \end{aligned}$$

for $0\le a\le A$ and falling factorials $z^{\underline{a}}:=z(z-1)\cdots (z-a+1)$.

The polynomials $\frac{1}{a!}(U-1)^{{\underline{a}}}$, $0\le a\le A$, are clearly a basis of the space of polynomials in *U* of degree at most *A*. Thus, there exist rational numbers $b_0, \ldots , b_A$ such that

$$\begin{aligned} \frac{U^A}{A!}=\sum _{0 \le a \le A} \frac{b_a}{a!} (U-1)^{\underline{a}}. \end{aligned}$$

Comparing the coefficients of $U^A$ shows that $b_A=1$. Substitution of *U* by $N-n$, multiplication by *f*(*n*) and summation over $1\le n<N$ yield

$$\begin{aligned} \frac{1}{A!}\sum _{1\le n<N}(N-n)^A f(n) = \sum _{0 \le a \le A} b_a ({\mathcal {S}}^{a+1}f)(N) \end{aligned}$$

by (14.14). When inserting the asymptotic expressions from (14.12), the summands involving fluctuations for $0\le a< A$ are absorbed by the error term  of the summand for $a=A$ because $\mathfrak {R}\gamma - \gamma _0 < 1$. Thus there are some constants $c_b$ for $0\le b<A$ such that14.15for integers $N\rightarrow \infty$.

If $\gamma +A=b+\chi _{\ell '}$ for some $0\le b<A$ and $\ell '\in {\mathbb {Z}}$, then we assume without loss of generality that $c_{b}=0$: Otherwise, we replace $\Psi _{A(m-1)}(u)$ by $\Psi _{A(m-1)}(u) + c_{b}\exp (-\,2\ell '\pi i u)$ and $c_{b}$ by 0. Both (14.15) and (14.13) remain intact: the former trivially, the latter because the factor for $a=A-b$ in (14.13) equals $\gamma +A-b-\chi _{\ell '} + Z=Z$ which compensates the fact that the Fourier coefficient $\psi _{A(m-1)(-\,\ell ')}$ is modified.

*Mellin–Perron summation.* We use the *A*th order Mellin–Perron summation formula to write the main contribution of ${\mathcal {S}}^{A+1} f$ as determined above in terms of new periodic fluctuations $\Xi _j$ whose Fourier coefficients are expressed in terms of residues of a suitably modified version of the Dirichlet generating function ${\mathcal {F}}$.

Without loss of generality, we assume that $\sigma _{\mathrm {abs}}>0$: the growth condition (6.8) trivially holds with $\eta =0$ on the right of the abscissa of absolute convergence of the Dirichlet series. By the *A*th order Mellin–Perron summation formula (see [18, Theorem 2.1]), we have

$$\begin{aligned} \frac{1}{A!}\sum _{1\le n<N}(N-n)^A f(n) = \frac{1}{2\pi i}\int _{\sigma _{\mathrm {abs}}+1-i\infty }^{\sigma _{\mathrm {abs}}+1+i\infty } \frac{{\mathcal {F}}(s) N^{s+A}}{s(s+1)\cdots (s+A)}\,{\mathrm {d}}s \end{aligned}$$

with the arbitrary choice $\sigma _{\mathrm {abs}}+1>\sigma _{\mathrm {abs}}$ for the real part of the line of integration.

The growth condition (6.8) allows us to shift the line of integration to the left such thatThe summand for *a* in the second term corresponds to a possible pole at $s=-a$ which is not taken care of in the first sum; note that ${\mathcal {F}}(s)$ is analytic at $s=-a$ in this case by assumption because of $\gamma _0<-a$.

We now compute the residue at $s=\gamma +\chi _\ell$. We use

$$\begin{aligned} N^{s+A} = N^{\gamma +A+\chi _\ell }\sum _{k\ge 0}\frac{(\log N)^k}{k!} (s-\gamma -\chi _\ell )^k \end{aligned}$$

to split up the residue aswith14.16for $j\ge -1$. Note that we allow $j=-1$ for the case of $\gamma \in -a+\frac{2\pi i}{\log q}{\mathbb {Z}}$ for some $1\le a\le A$ when ${\mathcal {F}}(s)/\bigl (s\cdots (s+A)\bigr )$ might have a pole of order $m+1$ at $s=-a$. Using the growth condition (6.8) and the choice of *A* yields14.17for $|\mathfrak {I}s |\rightarrow \infty$ and *s* which are at least a distance $\delta$ away from the poles $\gamma +\chi _\ell$. By writing the residue in (14.16) in terms of an integral over a rectangle around $s=\gamma +\chi _\ell$ (distance again at least $\delta$ away from $\gamma +\chi _\ell$), we see that (14.17) implies14.18for $|\ell |\rightarrow \infty$. Moreover, by (14.17), we see thatThus we proved that14.19for

14.20 $$\begin{aligned} \Xi _j(u) =\sum _{\ell \in {\mathbb {Z}}}\xi _{j\ell } \exp (2\ell \pi i u) \end{aligned}$$

where the $\xi _{j\ell }$ are given in (14.16). By (14.18), the Fourier series (14.20) converges uniformly and absolutely. This implies that $\Xi _j$ is a 1-periodic continuous function.

*Fourier Coefficients.* We will now compare the two asymptotic expressions for ${\mathcal {S}}^{A+1} f$ obtained so far to see that the fluctations coincide. We know explicit expressions for the Fourier coefficients of the $\Xi _j$ in terms of residues, and we know how the Fourier coefficients of the fluctuations of the repeated summatory function are related to the Fourier coefficients of the fluctuations of *F*. Therefore, we are able to compute the latter.

By (14.15), (14.19), elementary asymptotic considerations for the terms $N^b$ with $b>\mathfrak {R}\gamma +A$, Lemma 14.4 and the fact that $c_{b}=0$ if $b\in \gamma +A+\frac{2\pi i}{\log q}{\mathbb {Z}}$ for some $0\le b<A$, we see that $\Xi _j=\Psi _{Aj}$ for $-\,1\le j<m$. This immediately implies that ${\mathcal {F}}(0)=0$ if $\gamma _0<0$ and $\gamma \notin \frac{2\pi i}{\log q}{\mathbb {Z}}$.

To compute the Fourier coefficients $\psi _{Aj\ell }=\xi _{j\ell }$, we set $Z:=s-\gamma -\chi _\ell$ to rewrite (14.16) using (6.7) as

$$\begin{aligned} \psi _{Aj\ell }=[Z^{-1}] \frac{\sum _{b\ge 0}\varphi _{b\ell }Z^{b-j-1}}{\prod _{1 \le a \le A} (\gamma +a+\chi _\ell +Z)} =[Z^{j}] \frac{\sum _{b\ge 0}\varphi _{b\ell } Z^{b}}{\prod _{1 \le a \le A} (\gamma +a+\chi _\ell +Z)} \end{aligned}$$

for $-\,1\le j<m$ and $\ell \in {\mathbb {Z}}$. This is equivalent tofor $\ell \in {\mathbb {Z}}$ and $Z\rightarrow 0$. Clearing the denominator and using (14.13) as announced in Remark 14.3 lead tofor $\ell \in {\mathbb {Z}}$ and $Z\rightarrow 0$. Comparing coefficients shows that $\psi _{0j\ell }=\varphi _{j\ell }$ for $0\le j<m$ and $\ell \in {\mathbb {Z}}$. This proves (6.9). $\square$

## Proof of Theorem A

### Proof of Theorem A

By Remark 3.2, we have $x(n)=e_1 f(n)v(0)$. If $v(0)=0$, there is nothing to show. Otherwise, as observed in Sect. 7.1, *v*(0) is a right eigenvector of $A_0$ associated to the eigenvalue 1. As a consequence, *Kv*(0), $\vartheta _m v(0)$ and $\vartheta v(0)$ all vanish. Therefore, (3.3) follows from Theorem C by multiplication by $e_1$ and *v*(0) from left and right, respectively. Note that the notation is somewhat different: Instead of powers $(\log _q N)^k$ in Theorem C we write $(\log N)^k/k!$ here.

The functional equation (3.4) follows from Theorem D for $n_0=1$ by multiplication from right by *v*(0).

For computing the Fourier coefficients, we denote the rows of *T* by $w_1, \ldots , w_d$. Thus $w_a$ is a generalised left eigenvector of *C* of some order $m_a$ associated to some eigenvalue $\lambda _a$ of *C*. We can write $e_1=\sum _{1 \le a \le d} c_a w_a$ for some suitable constants $c_1, \ldots , c_d$. For $1\le a\le d$, we consider the sequence $h_a$ on ${\mathbb {Z}}_{>0}$ with

$$\begin{aligned} h_a(n)=w_a\bigl (v(n)+v(0)[ n=1 ]\bigr ). \end{aligned}$$

The reason for incorporating *v*(0) into the value for $n=1$ is that the corresponding Dirichlet series ${\mathcal {H}}^{(a)}(s):=\sum _{n\ge 1}n^{-s}h_a(n)$ only takes values at $n\ge 1$ into account. By definition, we have ${\mathcal {H}}^{(a)}(s)=w_av(0) + w_a{\mathcal {V}}(s)$. Taking the linear combination yields $\sum _{1 \le a \le d} c_a{\mathcal {H}}^{(a)}(s)=x(0) + {\mathcal {X}}(s)$. We choose $\gamma _0> \log _q R$ such that there are no eigenvalues $\lambda \in \sigma (C)$ with $\log _q R<\log _q\lambda \le \gamma _0$ and such that $\gamma _0\notin {\mathbb {Z}}_{\le 0}$.

By Theorem B, we have15.1for $N\rightarrow \infty$ for suitable 1-periodic Hölder continuous functions $\Psi _{ak}$ (which vanish if $|\lambda _a |\le R$). By Theorem D, the Dirichlet series ${\mathcal {H}}^{(a)}(s)$ is meromorphic for $\mathfrak {R}s>\gamma _0$ with possible poles at $s=\log _q \lambda _a + \chi _\ell$ for $\ell \in {\mathbb {Z}}$.

The sequence $h_a$ satisfies the prerequisites of Theorem E, either with $\gamma =\log _q \lambda _a$ if $\mathfrak {R}\log _q \lambda _a>\gamma _0$ or with arbitrary real $\gamma >\gamma _0$ and $\Phi _j=0$ for all *j* if $\mathfrak {R}\log _q \lambda _a \le \gamma _0$. The theorem then implies that

15.2 $$\begin{aligned} {\mathcal {H}}^{(a)}(0) = 0 \end{aligned}$$

if $$\gamma _0<0$$ and $$\lambda _a\ne 1$$.

If $|\lambda _a |>R$, Theorem E also yields

$$\begin{aligned} \Psi _{ak}(u)=\sum _{\ell \in {\mathbb {Z}}}\psi _{ak\ell }\exp (2\pi i\ell u) \end{aligned}$$

where the $\psi _{ak\ell }$ are given by the singular expansion

15.3 $$\begin{aligned} \frac{{\mathcal {H}}^{(a)}(s)}{s}\asymp \sum _{\ell \in {\mathbb {Z}}}\sum _{0\le k<m_a}\frac{\psi _{ak\ell }}{(s-\log _q\lambda _a-\chi _\ell )^{k+1}} \end{aligned}$$

for $\mathfrak {R}s>\gamma _0$. Note that (15.2) ensures that there is no additional pole at $s=0$ when $\gamma _0<0$ and $\lambda _a\ne 1$. Also note that in comparison to Theorem E, $\Phi _{m_a-1-k}$ there corresponds to $\Psi _{ak}$ here.

We now have to relate the results obtained for the sequences $h_a$ with the results claimed for the original sequence *f*. For $\lambda \in \sigma (C)$ with $|\lambda |>R$, we have

$$\begin{aligned} \Phi _{\lambda k}(u)=\sum _{\begin{array}{c} 1\le a\le d\\ \lambda _a=\lambda \end{array}}c_a\Psi _{ak}(u). \end{aligned}$$

We denote the Fourier coefficients of $\Phi _{\lambda k}$ by $\varphi _{\lambda k\ell }$ for $\ell \in {\mathbb {Z}}$ and will show that these Fourier coefficients actually fulfil (3.5). Taking linear combinations of (15.3) shows that

$$\begin{aligned} \sum _{\begin{array}{c} 1\le a\le d\\ \lambda _a=\lambda \end{array}}\frac{c_a{\mathcal {H}}^{(a)}(s)}{s} \asymp \sum _{\ell \in {\mathbb {Z}}}\sum _{0\le k<m(\lambda )}\frac{\varphi _{\lambda k\ell }}{(s-\log _q\lambda -\chi _\ell )^{k+1}} \end{aligned}$$

for $$\mathfrak {R}s>\gamma _0$$.

Summing over all $\lambda \in \sigma (C)$ yields (3.5) because summands $\lambda$ with $|\lambda |\le R$ are analytic for $\mathfrak {R}s>\gamma _0$ and do therefore not contribute to the right-hand side. $\square$

It might seem to be somewhat artificial that Theorem E is used to prove that ${\mathcal {H}}^{(j)}(0)=0$ in some of the cases above. In fact, this can also be shown directly using the linear representation; we formulate and prove this in the following remark.

### Remark 15.1

With the notations of the proof of Theorem A, ${\mathcal {H}}^{(j)}(0)=0$ if $\lambda _j\ne 1$ and $R<1$ can also be shown using the functional equation (3.4).

### Proof

We prove this by induction on $m_j$. By definition of *T*, we have $w_j(C-\lambda _j I)=[ m_j>1 ]w_{j+1}$. (We have $m_d=1$ thus $w_{d+1}$ does not actually occur.) If $m_j>1$, then ${\mathcal {H}}^{(j+1)}(0)=0$ by induction hypothesis.

We add $(I-q^{-s})\,v(0)$ to (3.4) and get

$$\begin{aligned}&\bigl (I-q^{-s}C\bigr )\bigl (v(0)+{\mathcal {V}}(s)\bigr ) = \bigl (I-q^{-s}C\bigr )v(0) + \sum _{1 \le n< q} n^{-s}v(n) \\&\quad + q^{-s}\sum _{0 \le r < q} A_r \sum _{k\ge 1}\left( {\begin{array}{c}-s\\ k\end{array}}\right) \Bigl (\frac{r}{q}\Bigr )^k {\mathcal {V}}(s+k). \end{aligned}$$

Multiplication by $w_j$ from the left yields

$$\begin{aligned} \bigl (1-q^{-s}\lambda \bigr ){\mathcal {H}}^{(j)}(s)&= [ m_j>1 ]\,q^{-s}{\mathcal {H}}^{(j+1)}(s) \\&\quad + w_j \bigl (I - q^{-s}C\bigr )v(0) + w_j\sum _{1 \le n< q} n^{-s}v(n) \\&\quad + w_jq^{-s}\sum _{0 \le r < q} A_r \sum _{k\ge 1}\left( {\begin{array}{c}-s\\ k\end{array}}\right) \Bigl (\frac{r}{q}\Bigr )^k {\mathcal {V}}(s+k). \end{aligned}$$

As $R<1$ and $\lambda _j\ne 1$, the Dirichlet series ${\mathcal {H}}^{(j)}(s)$ is analytic in $s=0$ by Theorem D. It is therefore legitimate to set $s=0$ in the above equation. We use the induction hypothesis that ${\mathcal {H}}^{(j+1)}(0)=0$ as well as the fact that $v(n)=A_nv(0)$ (note that *v*(0) is a right eigenvector of $A_0$ to the eigenvalue 1; see Sect. 7.1) for $0\le n<q$ to get

$$\begin{aligned} (1-\lambda ){\mathcal {H}}^{(j)}(0)=w_j\sum _{0 \le n < q} A_n v(0) -w_jCv(0) = 0 \end{aligned}$$

because all binomial coefficients $$\left( {\begin{array}{c}0\\ k\end{array}}\right) $$ vanish. $\square$

## Proof of Proposition 6.4

### Proof of Proposition 6.4

We set

$$\begin{aligned} j_0:=\left\lfloor {-\frac{p\bigl (\pi +\arg (\lambda )\bigr )}{2\pi }}\right\rfloor +1 \end{aligned}$$

with the motive that

$$\begin{aligned} -\pi <\arg (\lambda ) + \frac{2j\pi }{p}\le \pi \end{aligned}$$

holds for $j_0\le j<j_0+p$. This implies that for $j_0\le j<j_0+p$, the *p*th root of unity $\zeta _j:=\exp (2j\pi i/p)$ runs through the elements of $U_p$ such that $\log _q(\lambda \zeta _j)=\log _q(\lambda ) + 2j\pi i/(p\log q)$. Then

$$\begin{aligned} N^{\log _q(\zeta _j\lambda )}&= N^{\log _q \lambda } \exp \Bigl (\frac{2j\pi i}{p}\log _q N\Bigr )\\&= N^{\log _q \lambda } \exp (2j\pi i\log _{q^p} N) = N^{\log _q \lambda } \exp (2j\pi i\{ \log _{q^p} N \}). \end{aligned}$$

We set

$$\begin{aligned} \Phi (u):=\sum _{j_0\le j<j_0+p} \exp \Bigl (\frac{2j\pi i}{p}u\Bigr )\Phi _{(\zeta _j\lambda )}(u), \end{aligned}$$

thus $\Phi$ is a *p*-periodic function.

For the Fourier series expansion, we getReplacing $\ell p+j$ by $\ell$ leads to the Fourier series claimed in the proposition. $\square$

## Part IV: Computational Aspects

The basic idea for computing the Fourier coefficients is to use the functional equation in Theorem D. This part describes in detail how this is done. We basically follow an approach found in Grabner and Hwang [25] and Grabner and Heuberger [23], but provide error bounds.

An actual implementation is also available; SageMath [38] code can be found at https://gitlab.com/dakrenn/regular-sequence-fluctuations . We use the Arb library [33] (more precisely, its SageMath bindings) for ball arithmetic which keeps track of rounding errors such that we can be sure about the precision and accuracy of our results.

We use the results of this part to compute Fourier coefficients for our examples, in particular for esthetic numbers (Sect. 9) and Pascal’s rhombus (Sect. 10).

## Strategy for Computing the Fourier Coefficients

The computation of the Fourier coefficients relies on the evaluation of Dirichlet series at certain points $s=s_0$. It turns out to be numerically preferable to split up the sum as

$$\begin{aligned} {\mathcal {F}}_{1}(s_0) = \sum _{1 \le n < n_0} n^{-s_0} f(n) + {\mathcal {F}}_{n_0}(s_0) \end{aligned}$$

for some suitable $n_0$ (see Sect. 18.2), compute the sum of the first $n_0-1$ summands directly and evaluate ${\mathcal {F}}_{n_0}(s_0)$ as it is described in the following.

For actually computing the Fourier coefficients, we use a formulation in terms of a residue; for instance, see (3.6) where this is formulated explicitly in the set-up of Theorem A. As said, we will make use of the functional equation (6.3) for the matrix-valued Dirichlet series ${\mathcal {F}}_{n_0}(s)$ with its right-hand side, the matrix-valued Dirichlet series ${\mathcal {G}}_{n_0}(s)$.

Let us make this explicit for a simple eigenvalue $\lambda \ne 1$ of *C* and a corresponding eigenvector *w*. Then $w (I - q^{-s} C) = w (1 - q^{-s}\lambda )$ and (6.3) can be rewritten as

$$\begin{aligned} w\, {\mathcal {F}}_{1}(s) = \frac{1}{1 - q^{-s}\lambda } w\, {\mathcal {G}}_{1}(s). \end{aligned}$$

Thus, $w\, {\mathcal {F}}_{1}(s)$ has simple poles at $s=\log _q\lambda +\chi _\ell$ for all $\ell \in {\mathbb {Z}}$, where $\chi _\ell =\frac{2\ell \pi i}{\log q}$. By (6.7) and (6.9) of Theorem E (with $\kappa =\log _q\lambda$ and $m=1$), the $\ell$th Fourier coefficient is given by the residueNote that $\log q$ is the derivative of $1 - q^{-s}\lambda$ with respect to *s* evaluated at the pole $s=\log _q\lambda$.

By (6.4), ${\mathcal {G}}_{n_0}(\log _q\lambda +\chi _\ell )$ is expressed in terms of an infinite sum containing ${\mathcal {F}}_{n_0}(\log _q\lambda +\chi _\ell +k)$ for $k\ge 1$. We truncate this sum and bound the error; this is the aim of Sect. 18.1 and in particular Lemma 18.2. We can iterate the above idea for the shifted Dirichlet series ${\mathcal {F}}_{n_0}(\log _q\lambda +\chi _\ell +k)$ which leads to a recursive evaluation scheme. Note that once we have computed ${\mathcal {G}}_{n_0}(\log _q\lambda +\chi _\ell +k)$, we get ${\mathcal {F}}_{n_0}(\log _q \lambda +\chi _\ell +k)$ by solving a system of linear equations.

## Details on the Numerical Computation

### Bounding the Error

We need to estimate the approximation error which arises if the infinite sum over $k\ge 1$ in (6.4) is replaced by a finite sum. It is clear that for large $\mathfrak {R}s$ and $n_0$, the value ${\mathcal {F}}_{n_0}(s)$ will approximately be of the size of its first summand $n_0^{-s} f(n_0)$. In view of , this will be rather small. We give a precise estimate in a first lemma.

#### Lemma 18.1

Let $n_0> 1$ and let $M:=\max _{0\le r<q} ||A_r ||$. For $\mathfrak {R}s>\log _q M + 1$, we have

$$\begin{aligned} \sum _{n\ge n_0}\frac{||f(n) ||}{n^{\mathfrak {R}s}}\le \frac{M}{(\mathfrak {R}s-\log _q M -1)(n_0-1)^{\mathfrak {R}s-\log _q M -1}}. \end{aligned}$$

#### Proof

By definition of *M*, we have $||f(n) ||\le M^{1+\log _q n}=M n^{\log _q M}$. Therefore, we have

$$\begin{aligned} \sum _{n\ge n_0}\frac{||f(n) ||}{n^{\mathfrak {R}s}}&\le M\sum _{n\ge n_0}\frac{1}{n^{\mathfrak {R}s - \log _q M}}\le M\int _{n_0-1}^\infty \frac{{\mathrm {d}}n}{n^{\mathfrak {R}s - \log _q M}}\\ {}&=\frac{M}{(\mathfrak {R}s-\log _q M -1)(n_0-1)^{\mathfrak {R}s-\log _q M -1}} \end{aligned}$$

where we interpret the sum as a lower Riemann sum of the integral. $\square$

We now give a bound for the approximation error in (6.4).

#### Lemma 18.2

Let $n_0>1$ and *M* as in Lemma 18.1. Let $K\ge 1$ and $s\in {\mathbb {C}}$ be such that $\mathfrak {R}s+K>\max (\log _q M + 1, 0)$.

Then

$$\begin{aligned}&\Bigl \Vert {{\mathcal {G}}_{n_0}(s) - \sum _{n_0\le n<qn_0} n^{-s}f(n) - q^{-s}\sum _{0\le r<q}A_r\sum _{1\le k<K}\left( {\begin{array}{c}-s\\ k\end{array}}\right) \Bigl (\frac{r}{q}\Bigr )^k {\mathcal {F}}_{n_0}(s+k)}\Bigr \Vert \\&\quad \le q^{-\mathfrak {R}s}\left| {\left( {\begin{array}{c}-s\\ K\end{array}}\right) }\right| \frac{M}{(\mathfrak {R}s+K-\log _q M -1)(n_0-1)^{\mathfrak {R}s+K-\log _q M -1}}\sum _{0\le r<q}||A_r ||\Bigl (\frac{r}{q}\Bigr )^K. \end{aligned}$$

#### Proof

We set

$$\begin{aligned} D:={\mathcal {G}}_{n_0}(s) - \sum _{n_0\le n<qn_0} n^{-s}f(n) - q^{-s}\sum _{0\le r<q}A_r\sum _{1\le k<K}\left( {\begin{array}{c}-s\\ k\end{array}}\right) \Bigl (\frac{r}{q}\Bigr )^k {\mathcal {F}}_{n_0}(s+k) \end{aligned}$$

and need to estimate $||D ||$.

By definition of ${\mathcal {G}}_{n_0}(s)$, we have

$$\begin{aligned} {\mathcal {G}}_{n_0}(s)&= (1-q^{-s}C){\mathcal {F}}_{n_0}(s)\\&=\sum _{n_0\le n<qn_0} n^{-s}f(n) + {\mathcal {F}}_{qn_0}(s) - q^{-s}C{\mathcal {F}}_{n_0}(s)\\&=\sum _{n_0\le n<qn_0} n^{-s}f(n) +\sum _{0\le r<q}\sum _{n\ge n_0}\frac{A_r f(n)}{(qn+r)^s}- q^{-s}C{\mathcal {F}}_{n_0}(s)\\&=\sum _{n_0\le n<qn_0} n^{-s}f(n) +q^{-s}\sum _{0\le r<q}A_r \sum _{n\ge n_0}\frac{f(n)}{n^s}\Bigl (\Bigl (1+\frac{r}{qn}\Bigr )^{-s}- 1\Bigr ). \end{aligned}$$

Thus we have

$$\begin{aligned} D = q^{-s}\sum _{0\le r<q}A_r \sum _{n\ge n_0}\frac{f(n)}{n^s}\biggl (\Bigl (1+\frac{r}{qn}\Bigr )^{-s}- \sum _{0\le k<K}\left( {\begin{array}{c}-s\\ k\end{array}}\right) \Bigl (\frac{r}{qn}\Bigr )^k\biggr ). \end{aligned}$$

For $0\le x<1$, Taylor’s theorem (or induction on $K\ge 1$ using integration by parts) implies that

$$\begin{aligned} (1+x)^{-s}-\sum _{0\le k<K}\left( {\begin{array}{c}-s\\ k\end{array}}\right) x^k = K\int _{0}^x \left( {\begin{array}{c}-s\\ K\end{array}}\right) (1+t)^{-s-K}(x-t)^{K-1}\,{\mathrm {d}}t. \end{aligned}$$

For $0\le t\le x<1$, we can bound $|(1+t)^{-s-K} |$ from above by 1 since we have assumed that $\mathfrak {R}s + K>0$. Thus

$$\begin{aligned} \left| {(1+x)^{-s}-\sum _{0\le k<K}\left( {\begin{array}{c}-s\\ k\end{array}}\right) x^k}\right| \le K\left| {\left( {\begin{array}{c}-s\\ K\end{array}}\right) }\right| \int _{0}^x (x-t)^{K-1}\,{\mathrm {d}}t = \left| {\left( {\begin{array}{c}-s\\ K\end{array}}\right) }\right| x^K. \end{aligned}$$

Thus we obtain the bound

$$\begin{aligned} ||D || \le q^{-\mathfrak {R}s}\left| {\left( {\begin{array}{c}-s\\ K\end{array}}\right) }\right| \sum _{0\le r<q}||A_r ||\Bigl (\frac{r}{q}\Bigr )^K\sum _{n\ge n_0}\frac{||f(n) ||}{n^{\mathfrak {R}\sigma +K}}. \end{aligned}$$

Bounding the remaining Dirichlet series by Lemma 18.1 yields the result. $\square$

### Choices of Parameters

As mentioned at the beginning of this part, we choose the Arb library [33] for reliable numerical ball arithmetic. In our examples (esthetic numbers in Sect. 9 and Pascal’s rhombus in Sect. 10), we choose $n_0=1024$ and recursively compute ${\mathcal {F}}_{n_0}(\log _q\lambda + \chi _\ell +k)$ for $k\ge 1$ by (6.4). In each step, we keep adding summands for $k\ge 1$ until the bound of the approximation error in Lemma 18.2 is smaller than the smallest increment which can still be represented with the chosen number of bits. For plotting the graphs, we simply took machine precision; for the larger number of significant digits in Table 2, we used 128 bits precision.

## Non-vanishing Coefficients

Using reliable numerical arithmetic for the computations (see above) yields small balls in which the true value of the Fourier coefficients is. If such a ball does not contain zero, we know that the Fourier coefficient does not vanish. If the ball contains zero, however, we cannot decide whether the Fourier coefficient vanishes. We can only repeat the computation with higher precision and hope that this will lead to a decision that the coefficient does not vanish, or we can try to find a direct argument why the Fourier coefficient does indeed vanish, for instance using the final statement of Theorem B (3).

Vanishing Fourier coefficients appear in our introductory Example 3.1: In its continuation (Example 3.3) an alternative approach is used to compute these coefficients explicitly symbolically. In this way a decision for them being zero is possible. The same is true for the example of transducers in Sect. 8.

It should also be noted that in the analysis of esthetic numbers (example in Sect. 9) we could have modelled the problem by a complete transducer (by just introducing a sink) and then applied the results of Sect. 8. This would have led to an asymptotic expansion where the fluctuations of the main term (corresponding to the eigenvalue *q*) would in fact have vanished, but an argument would have been needed. So we chose a different approach in Sect. 9 to avoid this problem. There the eigenvalue *q* does no longer occur. This implies that the fluctuations for *q* of the transducer approach vanish. Note also that half of the remaining fluctuations still turn out to vanish: this is shown in the proof of Corollary G.

## Footnotes

1 In the standard literature [2, 3] these sequences are called *k*-regular sequences (instead of *q*-regular sequences).

2 The code accompanying this article can be found at https://gitlab.com/dakrenn/regular-sequence-fluctuations . It is meant to be used with the open source mathematics software SageMath [38].

3 We use a functional notation for sequences, i.e., a sequence *x* on ${\mathbb {Z}}_{\ge 0}$ is seen as function $x:{\mathbb {Z}}_{\ge 0} \rightarrow {\mathbb {C}}$.

4 Whenever we write that $r_{\ell -1}\ldots r_0$ is the *q*-ary digit expansion of *n*, we mean that $r_j\in \{ 0,\ldots , q-1 \}$ for $0\le j<\ell$, $r_{\ell -1}\ne 0$ and $n=\sum _{0 \le j < \ell } r_j q^j$. In particular, the *q*-ary expansion of zero is the empty word.

5 Note that the summatory function *X*(*N*) contains the summand *x*(0) but the Dirichlet series cannot. This is because the choice of including *x*(0) into *X*(*N*) will lead to more consistent results.

6 We use the notion of singular expansion as defined by Flajolet, Gourdon and Dumas [17, Definition 2]: it is the formal sum of the principal parts of a meromorphic function over all poles in the domain given.

7 If $1\in \sigma (C)$, then the matrix $C-I$ is singular. In that case, $\vartheta _0$ will never be used.

8 Chebyshev polynomials are frequently occurring phenomena in lattice path analysis, see for instance [7, 16]. We have such a lattice path here, so their appearance is not surprising.

9 Up to replacing 2*X* by *X*, the polynomials $U_n$ used here correspond to the polynomials $p_n$ used in [8].

10 Note that $v(0) = A_0 v(0)$ and $v(1) = A_1 v(0)$ are indeed true.

## References

[1] Allouche J-P, Mendès France M, Peyrière J (2000). Automatic Dirichlet series. J. Number Theory.

[2] Allouche J-P, Shallit J (1992). The ring of $$k$$-regular sequences. Theor. Comput. Sci..

[3] Allouche J-P, Shallit J (2003). Automatic Sequences: Theory, Applications, Generalizations.

[4] Allouche J-P, Shallit J (2003). The ring of $$k$$-regular sequences, II. Theor. Comput. Sci..

[5] Berthé V, Lhote L, Vallée B (2016). Probabilistic analyses of the plain multiple gcd algorithm. J. Symb. Comput..

[6] Berthé V, Rigo M (2010). Combinatorics, Automata and Number Theory. Encyclopedia of Mathematics and Its Applications.

[7] de Bruijn NG, Knuth DE, Rice SO (1972). The Average Height of Planted Plane Trees, Graph Theory and Computing.

[8] De Koninck J-M, Doyon N (2009). Esthetic numbers. Ann. Sci. Math. Québec.

[9] Delange H (1975). Sur la fonction sommatoire de la fonction “somme des chiffres”. Enseign. Math. (2).

[10] Drmota, M., Grabner, P.J.: Analysis of digital functions and applications, Combinatorics, automata and number theory (Valérie Berthé and Michel Rigo (eds.), Encyclopedia Math. Appl., vol. 135, pp. 452–504, Cambridge University Press, Cambridge (2010)

[11] Drmota M, Szpankowski W (2013). A master theorem for discrete divide and conquer recurrences. J. ACM.

[12] Dumas P (2013). Joint spectral radius, dilation equations, and asymptotic behavior of radix-rational sequences. Linear Algebra Appl..

[13] Dumas P (2014). Asymptotic expansions for linear homogeneous divide-and-conquer recurrences: algebraic and analytic approaches collated. Theor. Comput. Sci..

[14] Dumas P, Lipmaa H, Wallén J (2007). Asymptotic behaviour of a non-commutative rational series with a nonnegative linear representation. Discrete Math. Theor. Comput. Sci..

[15] Fekete M (1923). Über die Verteilung der Wurzeln bei gewissen algebraischen Gleichungen mit ganzzahligen Koeffizienten. Math. Z..

[16] Flajolet P (1980). Combinatorial aspects of continued fractions. Discrete Math..

[17] Flajolet P, Gourdon X, Dumas P (1995). Mellin transforms and asymptotics: harmonic sums. Theor. Comput. Sci..

[18] Flajolet P, Grabner P, Kirschenhofer P, Prodinger H, Tichy RF (1994). Mellin transforms and asymptotics: digital sums. Theor. Comput. Sci..

[19] Flajolet P, Odlyzko A (1990). Singularity analysis of generating functions. SIAM J. Discrete Math..

[20] Godsil CD, Royle G (2001). Algebraic Graph Theory, Graduate Texts in Mathematics.

[21] Goldwasser J, Klostermeyer W, Mays M, Trapp G (1999). The density of ones in Pascal’s rhombus. Discrete Math..

[22] Goč Daniel, Mousavi Hamoon, Shallit Jeffrey (2013). On the Number of Unbordered Factors. Language and Automata Theory and Applications.

[23] Grabner PJ, Heuberger C (2006). On the number of optimal base 2 representations of integers. Des. Codes Cryptogr..

[24] Grabner PJ, Heuberger C, Prodinger H (2005). Counting optimal joint digit expansions. Integers.

[25] Grabner PJ, Hwang H-K (2005). Digital sums and divide-and-conquer recurrences: Fourier expansions and absolute convergence. Constr. Approx..

[26] Graham RL, Knuth DE, Patashnik O (1994). Concrete Mathematics. A Foundation for Computer Science.

[27] Hardy GH, Riesz M (1915). The General Theory of Dirichlet’s Series. Cambridge Tracts in Mathematics and Mathematical Physics.

[28] Hardy GH, Wright EM (1975). An Introduction to the Theory of Numbers.

[29] Heuberger Clemens, Krenn Daniel (2019). Esthetic Numbers and Lifting Restrictions on the Analysis of Summatory Functions of Regular Sequences. 2019 Proceedings of the Sixteenth Workshop on Analytic Algorithmics and Combinatorics (ANALCO).

[30] Heuberger, C., Krenn, D., Prodinger, H.: Analysis of summatory functions of regular sequences: transducer and Pascal’s rhombus. In: Fill, J.A., Ward, M.D. (eds.) Proceedings of the 29th International Conference on Probabilistic, Combinatorial and Asymptotic Methods for the Analysis of Algorithms, Leibniz International Proceedings in Informatics (LIPIcs), vol. 110, Schloss Dagstuhl–Leibniz-Zentrum fuer Informatik, pp. 27:1–27:18 (2018)

[31] Heuberger C, Kropf S, Prodinger H (2015). Output sum of transducers: limiting distribution and periodic fluctuation. Electron. J. Combin..

[32] Hwang H-K, Janson S, Tsai T-H (2017). Exact and asymptotic solutions of a divide-and-conquer recurrence dividing at half: theory and applications. ACM Trans. Algorithms.

[33] Johansson F (2017). Arb: efficient arbitrary-precision midpoint-radius interval arithmetic. IEEE Trans. Comput..

[34] Jungers R (2009). The Joint Spectral Radius. Theory and Applications. Lecture Notes in Control and Information Sciences.

[35] Lagarias JC, Wang Y (1995). The finiteness conjecture for the generalized spectral radius of a set of matrices. Linear Algebra Appl..

[36] NIST Digital Library of Mathematical Functions. http://dlmf.nist.gov/. Release 1.0.16 of 2017-09-18 (2017), Olver, F.W.J., Daalhuis, A.B.O., Lozier, D.W., Schneider, B.I., Boisvert, R.F., Clark, C.W., Miller, B.R., Saunders, B.V. (eds.)

[37] Rota G-C, Strang G (1960). A note on the joint spectral radius. Indag. Math..

[38] SageMath Developers: SageMath Mathematics Software (Version 8.3) (2018). http://www.sagemath.org

[39] The On-Line Encyclopedia of Integer Sequences (2018). http://oeis.org

[40] Zygmund A (2002). Trigonometric Series.

